# Mapping the global, regional, and national burden of diarrheal diseases attributable to unsafe water

**DOI:** 10.3389/fpubh.2023.1302748

**Published:** 2023-12-06

**Authors:** Ling Chen, Jinghua Jiao, Shunming Liu, Lei Liu, Pengliang Liu

**Affiliations:** ^1^Department of Pharmacy, General Hospital of Benxi Iron and Steel Industry Group of Liaoning Health Industry Group, Benxi, China; ^2^Department of Anesthesiology, Guangzhou Eighth People's Hospital, Guangzhou Medical University, Guangzhou, Guangdong Province, China; ^3^Guangdong Eye Institute, Department of Ophthalmology, Guangdong Provincial People's Hospital (Guangdong Academy of Medical Sciences), Southern Medical University, Guangzhou, China; ^4^Department of Gastroenterology and Endoscopy, The Fourth Affiliated Hospital of China Medical University, Shenyang, China

**Keywords:** diarrheal diseases, unsafe water, global, disability-adjusted life years, death

## Abstract

**Background:**

Diarrheal diseases are major contributors to deaths. Data on global and country-specific levels and trends of diarrheal diseases resulting from unsafe water are essential for policymakers to allocate resources.

**Aims:**

This study aimed to describe the global, regional, and national spatiotemporal burden of diarrheal diseases resulting from unsafe water exposure.

**Methods:**

According to the Global Burden of Disease (GBD) 2019 dataset, deaths, disability-adjusted life years (DALYs) of diarrheal diseases, and their age-standardized rates (ASRs) were analyzed by age and sex in 204 countries and territories. Moreover, the average annual percentage change (AAPC) was estimated by a log-linear regression model to reflect the time trend. The association between ASR of diarrheal diseases due to unsafe water and socio-demographic index (SDI) levels was also analyzed.

**Results:**

From 1990 to 2019, the number of deaths and DALYs of diarrheal diseases resulting from unsafe water decreased by 50 and 59%, respectively. Moreover, the ASR of deaths and DALYs also decreased during the study period, with AAPCs of −3.69 (95% CI [95% confidence interval]: −3.91 to −3.47) and − 3.66 (95% CI: −3.8 to −3.52), respectively. High diarrheal diseases resulting from unsafe water occurred mainly in low SDI regions and Africa. Males exhibited greater diarrheal deaths attributable to unsafe water than females, which was contrary to the condition in terms of DALYs. The age-specific burden of diarrheal deaths attributable to unsafe water is concentrated in children younger than 5 years. The AAPCs of the ASR of both deaths and DALYs showed a strong negative correlation with the SDI levels.

**Conclusion:**

The current study indicated that the global burden of unsafe water exposure-related diarrheal diseases decreased from 1990 to 2019 and varied significantly according to age, sex, and geographical location. Effective health promotion and health communication strategies and policies should be adopted to prevent and control diarrheal diseases resulting from unsafe water exposure.

## Highlights

- Research background: Diarrheal diseases are major contributors to deaths. Data on global and country-specific levels and trends of diarrheal diseases resulting from unsafe water are essential for policymakers to allocate resources.
- Research motivation: The global, regional, and national spatiotemporal burden of diarrheal diseases resulting from unsafe water exposure is still unclear.
- Research objectives: To investigate the global, regional, and national spatiotemporal burden of diarrheal diseases resulting from unsafe water exposure.
- Research methods: Deaths and disability-adjusted life years (DALYs) of diarrheal diseases resulting from unsafe water were obtained from the Global Burden of Disease (GBD) 2019, and the data were stratified by the socio-demographic index (SDI) level.
- Research results: From 1990 to 2019, the number of deaths and DALYs of diarrheal diseases resulting from unsafe water decreased by 50 and 59%, respectively. High diarrheal diseases resulting from unsafe water occurred mainly in low SDI regions and Africa. The global diarrheal deaths attributable to unsafe water were greater in females than in males, which was contrary to the condition in terms of DALYs. Age-specific burden of diarrheal deaths attributable to unsafe water concentrated in children younger than 5 years. The AAPCs of the ASR of deaths and DALYs showed a strong negative correlation with the SDI levels.
- Research conclusion: Socioeconomic development status significantly affects the burden of diarrheal diseases resulting from unsafe water.
- Research perspectives: Our findings are valuable for implementing tailored prevention strategies for diarrheal diseases resulting from unsafe water exposure.

## Introduction

Diarrheal disease is one of the main concerns for public health worldwide. In terms of etiology, it can be divided into infectious diarrhea, including cholera, bacillary dysentery, amoebic dysentery, and rotavirus, and non-infectious diarrhea, such as dietary diarrhea, allergic diarrhea, and symptomatic diarrhea. According to data from the Global Burden of Diseases, Injuries, and Risk Factors Study (GBD) 2016, it provides an estimate of the burden of diarrhea in 195 countries. Diarrhea is the eighth leading cause of death among all ages and the fifth leading cause of death among children younger than 5 years worldwide (1). There are many factors related to diarrheal disease, including exposure to unsafe sources of water, unsafely managed sanitation, and animals (2).

Safe water is closely related to people’s daily lives and plays an important role in promoting human health. In 1854, British physician John Snow identified a public water pump as the outbreak’s source for an outbreak of cholera in London. After that, the role of unsafe water in the spread of the disease was fully recognized (3). According to GBD 2016, unsafe water was the second leading cause of diarrhea, responsible for 72.1% of diarrhea deaths in children younger than 5 years (1). However, there is no report on the global burden of diarrhea in detail.

To date, the GBD 2019 estimates over 369 diseases and injuries and 87 risk factors in 204 countries and territories from 1990 to 2019 (4, 5). In this study, up-to-date estimates of diarrhea deaths and burdens attributable to unsafe water are presented based on the date in GBD 2019. We describe unsafe water-related, spatial–temporal variations of diarrhea burden at global, regional, and national levels, by age, sex, and socio-demographic index (SDI).

## Methods

### Data resource

The data were originated from the GBD 2019 database released by the Institute for Health Metrics and Evaluation (IHME) of the University of Washington,^1^ aiming to assess the burden of 369 diseases/injuries, and 87 risk factors in 204 countries and territories from 1990 to 2019. The burden of diarrheal diseases attributable to unsafe water was measured by death, disability-adjusted life years (DALYs), years of life lost due to premature death (YLL), and years of life lost with disability (YLD). Total DALYs were estimated by adding YLL + YLD. Its measurements include absolute number (×1,000), rate, and age-standardized rate (ASR, ×1,000, per 100,000 population) according to the global standard population of GBD2019.

### Definition of diarrheal diseases

Diarrheal diseases were defined as three or more loose stools in a 24 h period. In the diarrhea models, self-reported prevalence is the reference category for all data adjustments. Hospital input data use ICD-9 (001–001.9, 003.8–006.9, 007.4–007.8, 008.2–009.9) and ICD-10 (A00–A00.9, A02–A02.0, A02.8–A07, A07.2–A07.4, A08–A09.9, K52.1, R19.7), and its mortality was estimated in the Cause of Death Ensemble modeling platform (CODEm). GBD 2019 excluded gastroenteritis as a case definition, as this is often syndromic (vomiting or diarrhea).

### Unsafe water

For GBD 2019, exposure to unsafe water was defined based on: (1) the reported primary water source used by the household and (2) the use of household water treatment (HWT) to improve the quality of drinking water before consumption. Water sources were defined based on the WHO/UNICEF Joint Monitoring Programme for Water Supply, Sanitation, and Hygiene (JMP, https://washdata.org/monitoring/drinking-water).

### SDI definition

The SDI value ranges from 0 to 1, where 0 indicates that the development level related to health outcomes in the region is theoretically the lowest. When the SDI value is 1, it indicates that the region has the highest theoretical level of development related to health outcomes. According to the SDI values of each country, it is divided into five levels: low, medium (middle low, middle, and middle high), and high SDI regions.

### Statistical analysis

Joinpoint regression was used to analyze the changes in disease burden and calculate the average annual percentage change (AAPC) as well as its 95% confidence interval (CI). Of which, all AAPC and its upper 95%CI greater than 0 indicate an upward trend, whereas AAPC and its lower 95%CI less than 0 indicate a downward trend. In addition, the associations between the burden of diarrheal diseases attributable to unsafe water and SDI were examined using Pearson’s correlation analysis. All statistics and analyses were performed using R software (version 5.0.4). A *p*-value of less than 0.05 was considered statistically significant.

## Results

### Diarrheal disease deaths attributable to unsafe water

**Figure 1:**
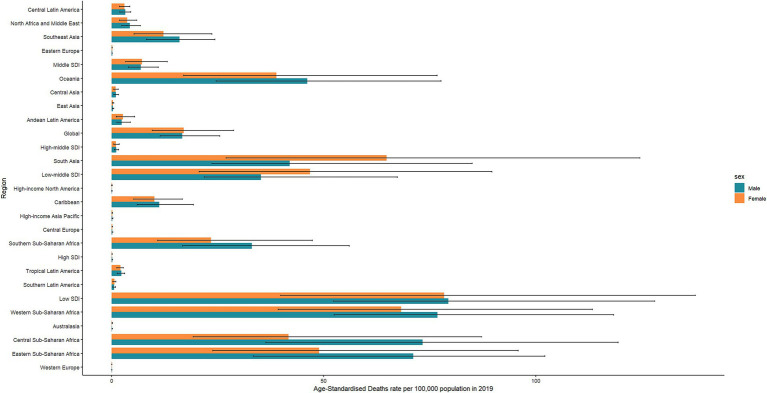
Sex-specific rates of deaths of diarrheal diseases attributable to unsafe water in 2019 for global and regions.

During the past three decades, the global deaths of diarrheal diseases attributable to unsafe water have decreased from 2442.1 (95%UI: 1765 to 3,147) in 1990 to 1230.2 (95%UI: 817.8 to 1788.9) in 2019, a 50% decrease. Additionally, the ASR of deaths decreased from 49.1 (95%UI: 34.5 to 65) to 16.8 (95%UI: 11.3 to 24.1), with an AAPC of −3.69 (95%CI: −3.91 to −3.47; Table 1). In terms of gender, the ASR of diarrheal disease-related deaths attributable to unsafe water was higher for females than males (Figure 1).

**Table 1:** Global diarrheal deaths attributable to unsafe water in 1990 and 2019 and the temporal trend from 1990 to 2019.

Regions	1990 Counts (thousand)	Age standardized rate (per 100,000 population), 1990	Crude rate (per 100,000 population), 1990	2019 Counts (thousand)	Age standardized rate (per 100,000 population), 2019	Crude rate (per 100,000 population), 2019	Average annual percent change	% change in number between 1990 and 2019
Global	2442.1 (1765 to 3,147)	49.1 (34.5 to 65)	45.6 (33 to 58.8)	1230.2 (817.8 to 1788.9)	16.8 (11.3 to 24.1)	15.9 (10.6 to 23.1)	−3.69 (−3.91 to −3.47)	−0.5 (−0.62 to −0.3)
High SDI	2.8 (1.5 to 4.4)	0.4 (0.2 to 0.6)	0.3 (0.2 to 0.5)	1.9 (0.7 to 3.6)	0.1 (0 to 0.2)	0.2 (0.1 to 0.4)	−4.84 (−5.03 to −4.64)	−0.33 (−0.65 to 0.05)
High-middle SDI	52.2 (36 to 67.8)	5.2 (3.6 to 6.8)	4.5 (3.1 to 5.9)	16.5 (8.9 to 26.3)	1 (0.6 to 1.6)	1.2 (0.6 to 1.8)	−5.51 (−5.68 to −5.34)	−0.68 (−0.77 to −0.57)
Middle SDI	382.5 (273.7 to 485.1)	29.6 (19.9 to 41.4)	22.3 (15.9 to 28.3)	137.7 (78 to 218.1)	7 (4 to 11.1)	5.7 (3.3 to 9.1)	−4.88 (−5.04 to −4.72)	−0.64 (−0.74 to −0.52)
Low-middle SDI	1126.9 (803.7 to 1490.6)	148 (97.4 to 205.4)	99.8 (71.1 to 132)	500.8 (303.9 to 790.7)	41.6 (24.5 to 67.2)	28.4 (17.2 to 44.8)	−4.38 (−4.85 to −3.9)	−0.56 (−0.7 to −0.34)
Low SDI	876.6 (635.1 to 1132.6)	211.4 (131.1 to 295.2)	166 (120.3 to 214.5)	572.6 (404.9 to 778.3)	79 (51.9 to 116.9)	50.7 (35.9 to 69)	−3.36 (−3.51 to −3.2)	−0.35 (−0.5 to −0.07)
Andean Latin America	8.8 (5.8 to 11.7)	20.9 (13.3 to 29.6)	23 (15.1 to 30.6)	1.4 (0.7 to 2.4)	2.5 (1.2 to 4.3)	2.3 (1.1 to 3.8)	−7.5 (−7.87 to −7.13)	−0.84 (−0.9 to −0.75)
Australasia	0 (0 to 0)	0.1 (0 to 0.1)	0.1 (0 to 0.1)	0.1 (0 to 0.1)	0.1 (0 to 0.2)	0.2 (0.1 to 0.4)	2.07 (1.61 to 2.53)	3.79 (2.21 to 5.93)
Caribbean	12.5 (9.2 to 15.9)	32.5 (23.8 to 42)	35.4 (26.1 to 45)	4.5 (2.6 to 6.8)	10.6 (5.9 to 16.5)	9.6 (5.5 to 14.5)	−4.54 (−5.89 to −3.16)	−0.64 (−0.77 to −0.47)
Central Asia	9.9 (6.2 to 12.9)	10.7 (6.7 to 13.9)	14.3 (9 to 18.6)	0.9 (0.5 to 1.3)	1 (0.5 to 1.4)	0.9 (0.5 to 1.4)	−8.13 (−8.56 to −7.7)	−0.91 (−0.94 to −0.88)
Central Europe	0.5 (0.3 to 0.7)	0.6 (0.4 to 0.8)	0.4 (0.3 to 0.6)	0.3 (0.1 to 0.5)	0.1 (0.1 to 0.3)	0.2 (0.1 to 0.4)	−4.48 (−5.09 to −3.87)	−0.47 (−0.66 to −0.25)
Central Latin America	38.7 (27.3 to 47.5)	24.5 (17.3 to 29.8)	23.6 (16.6 to 28.9)	7 (4.2 to 9.8)	3.1 (1.9 to 4.4)	2.8 (1.7 to 3.9)	−6.95 (−7.11 to −6.79)	−0.82 (−0.86 to −0.77)
Central Sub-Saharan Africa	74.2 (46.5 to 108)	143.9 (92.8 to 210.9)	133.6 (83.8 to 194.6)	49.5 (29.8 to 74.7)	55.9 (34.8 to 89.8)	37.6 (22.7 to 56.8)	−3.14 (−3.29 to −2.98)	−0.33 (−0.54 to −0.05)
East Asia	76.5 (57 to 96.7)	7.1 (5.2 to 9)	6.2 (4.6 to 7.9)	3.4 (1.8 to 5.6)	0.3 (0.1 to 0.4)	0.2 (0.1 to 0.4)	−10.69 (−10.98 to −10.4)	−0.96 (−0.97 to −0.93)
Eastern Europe	1.1 (0.7 to 1.5)	0.6 (0.4 to 0.9)	0.5 (0.3 to 0.7)	0.2 (0.1 to 0.3)	0.1 (0.1 to 0.2)	0.1 (0.1 to 0.2)	−6.05 (−6.7 to −5.39)	−0.8 (−0.85 to −0.75)
Eastern Sub-Saharan Africa	278.9 (203.7 to 359.1)	172 (105.1 to 255.1)	146.6 (107.1 to 188.8)	152.5 (103.6 to 207.1)	59 (34.9 to 88.8)	37 (25.2 to 50.3)	−3.57 (−3.84 to −3.29)	−0.45 (−0.61 to −0.21)
High-income Asia Pacific	0.4 (0.1 to 0.7)	0.3 (0.1 to 0.5)	0.2 (0.1 to 0.4)	0.7 (0.3 to 1.4)	0.1 (0 to 0.2)	0.4 (0.1 to 0.7)	−2.53 (−2.89 to −2.17)	0.75 (0.12 to 1.59)
High-income North America	0.1 (0 to 0.2)	0 (0 to 0.1)	0 (0 to 0.1)	0.4 (0.2 to 0.9)	0.1 (0 to 0.1)	0.1 (0 to 0.3)	2.93 (2.19 to 3.67)	3.8 (2.13 to 6.31)
North Africa and Middle East	87.9 (59.8 to 125.2)	18.9 (12.8 to 26.8)	25.5 (17.3 to 36.3)	21 (12.1 to 32.1)	4 (2.3 to 5.9)	3.4 (2 to 5.3)	−5.14 (−5.4 to −4.88)	−0.76 (−0.83 to −0.68)
Oceania	2.7 (1.9 to 3.6)	79.2 (50.9 to 118.9)	41.1 (28.6 to 55.6)	3.1 (2 to 4.6)	42.3 (25.2 to 66.6)	23.6 (15.1 to 34.3)	−2.15 (−2.22 to −2.08)	0.18 (−0.14 to 0.57)
South Asia	1150.3 (792.4 to 1548.2)	201.8 (127.1 to 286)	104.8 (72.2 to 141)	606.7 (336 to 1006.9)	54.1 (29.6 to 89.3)	33.6 (18.6 to 55.8)	−4.58 (−4.82 to −4.33)	−0.47 (−0.64 to −0.21)
Southeast Asia	198.7 (133.1 to 279.1)	59.3 (37.3 to 86.8)	42.6 (28.5 to 59.8)	68.2 (39.7 to 105.2)	13.8 (7.9 to 21.4)	10.1 (5.9 to 15.6)	−4.87 (−4.99 to −4.75)	−0.66 (−0.78 to −0.5)
Southern Latin America	1.1 (0.8 to 1.4)	2.4 (1.7 to 3)	2.3 (1.6 to 2.8)	0.5 (0.2 to 0.8)	0.6 (0.3 to 1)	0.7 (0.3 to 1.1)	−4.52 (−5.06 to −3.98)	−0.57 (−0.73 to −0.42)
Southern Sub-Saharan Africa	24.7 (17.5 to 32.9)	56.6 (37.9 to 81.5)	47 (33.4 to 62.7)	16 (9.6 to 24.8)	27.7 (16.3 to 45.1)	20.3 (12.3 to 31.5)	−2.47 (−2.73 to −2.21)	−0.35 (−0.51 to −0.19)
Tropical Latin America	33.7 (24.4 to 43.2)	23 (16.7 to 28.8)	22 (15.9 to 28.3)	4.4 (2.6 to 6)	2.2 (1.3 to 2.9)	2 (1.2 to 2.7)	−7.85 (−8.15 to −7.56)	−0.87 (−0.91 to −0.83)
Western Europe	0.2 (0.1 to 0.5)	0 (0 to 0.1)	0.1 (0 to 0.1)	0.5 (0.2 to 1)	0 (0 to 0.1)	0.1 (0 to 0.2)	−0.56 (−1.03 to −0.08)	0.97 (0.46 to 1.51)
Western Sub-Saharan Africa	441.3 (293.4 to 593)	206.6 (132.8 to 285.5)	229.1 (152.4 to 307.9)	288.7 (204.7 to 381.8)	72.4 (49.6 to 103.2)	63.3 (44.9 to 83.7)	−3.58 (−3.84 to −3.32)	−0.35 (−0.55 to −0.01)

At the SDI regional level, deaths from diarrheal diseases attributable to unsafe water were mainly observed in the low SDI region, with an ASR of 79 (95%UI: 51.9 to 116.9) in 2019, followed by low-middle and middle SDI regions. All SDI regions showed a downward trend in ASR from deaths from diarrheal diseases attributable to unsafe water, and the highest reduction was observed in the high-middle SDI region, with an AAPC of −5.51 (95%CI: −5.68 to −5.34, Table 1).

At the regional level, Western Sub-Saharan Africa, Eastern Sub-Saharan Africa, and Central Sub-Saharan Africa were the top three regions with the highest deaths in 2019, reaching an ASR of 72.4 (95%UI: 49.6 to 103.2), 59 (95%UI: 34.9 to 88.8), and 55.9 (95%UI: 34.8 to 89.8), respectively. Most regions experienced a declining trend in ASR of deaths, with East Asia being the fastest, with AAPC being −10.69 (95%CI: −10.98 to −10.4). However, there was only the Australasia region, where the ASR was escalating during the past 30 years, with an AAPC of 2.07 (95%CI: 1.61 to 2.53; Table 1). Females had a higher ASR of death in most Africa, Oceania, and Southeast Asia regions (Figure 1).

**Figure 2:**
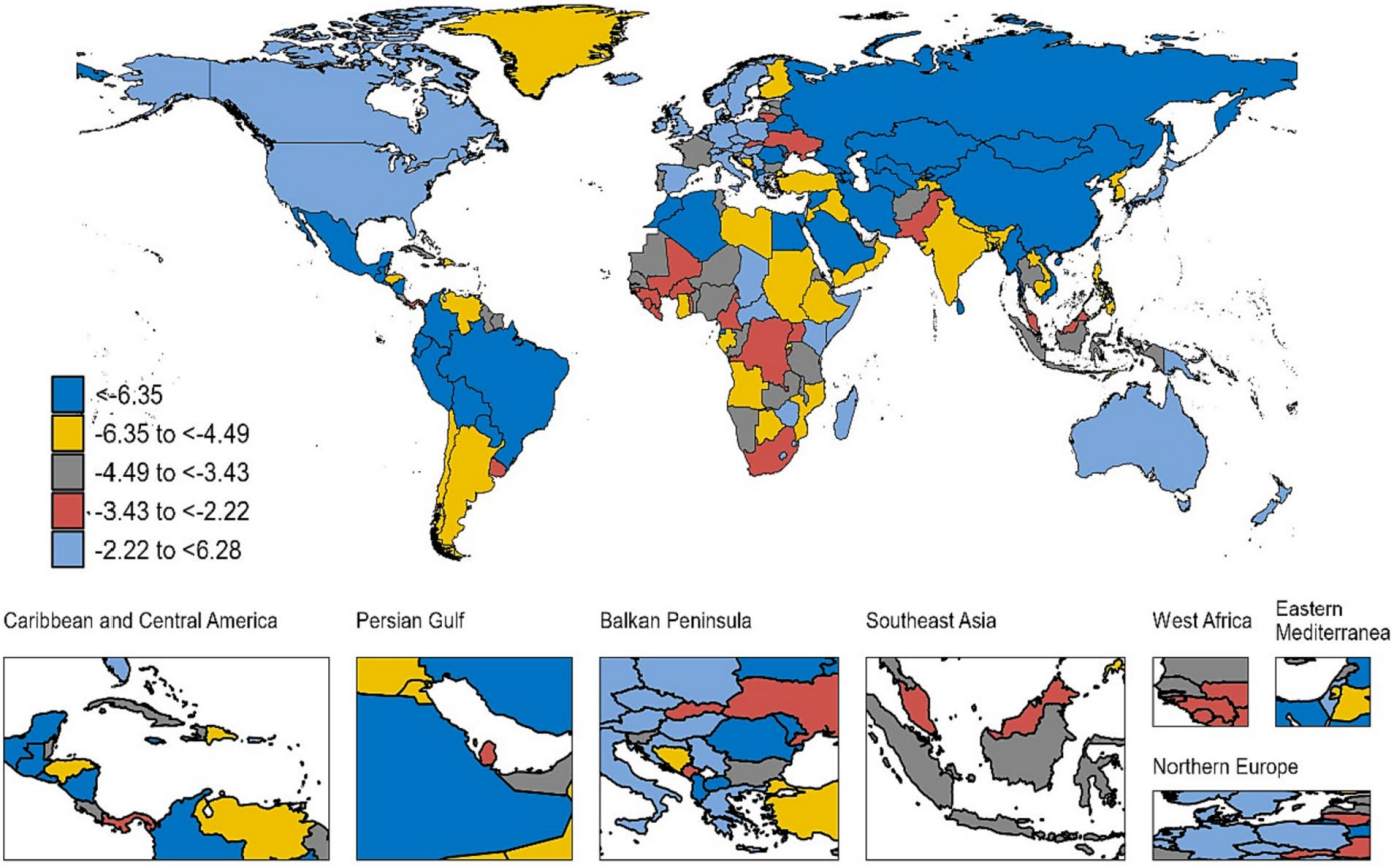
Average annual percent change (AAPC) of deaths of diarrheal diseases resulting from unsafe water by country for male and female sexes combined and all ages from 1990 to 2019.

**Figure 3:**
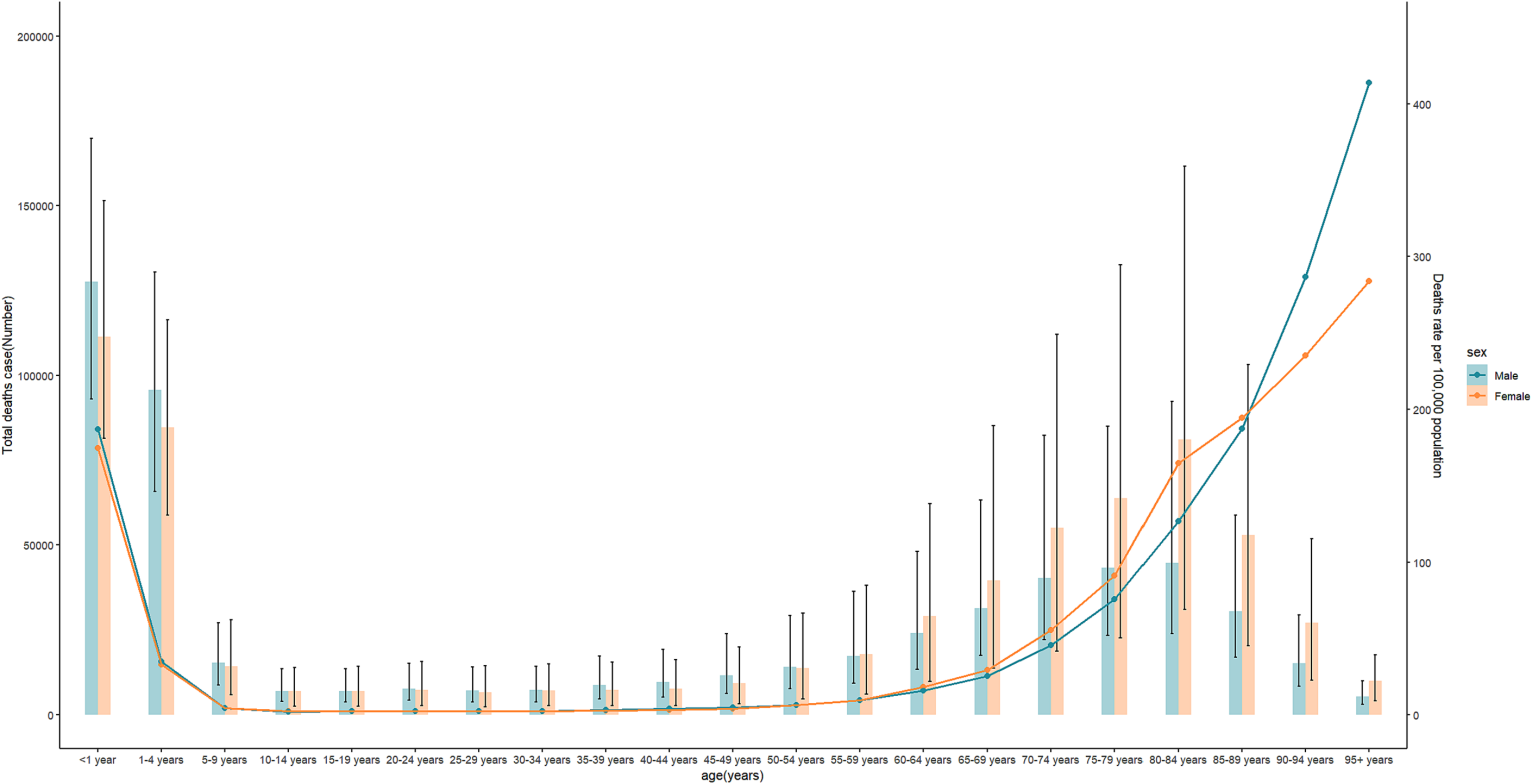
Age-specific rates of deaths of diarrheal diseases attributable to unsafe water by sex in 2019 for global and regions.

At the national level, India accounted for the largest deaths of diarrheal diseases attributable to unsafe water in 2019, at 508.3 (95%UI: 271 to 847.2), a decrease of 45% compared with 1990. A total of 29 countries experienced an increase in the number of deaths, and Hungary had the largest increase, with an 809% increment over 30 years. The highest ASR of deaths was found in the Central African Republic (197.6, 95%UI: 108.9 to 308.3), followed by Chad and Somalia. There were 18 countries (or territories) exhibiting a gradual increase in ASR of deaths, with Hungary (AAPC = 6.28, 95%CI: 5.28 to 7.3) being the fastest. Meanwhile, a total of 182 countries (or territories) observed an annually decreasing ASR of deaths, and the most significant rapid decrease was in Armenia (AAPC = −12.41, 95CI: −13.05 to −11.76) (Supplementary Table S1; Figure 2). In 2019, global deaths due to diarrheal diseases attributable to unsafe water peaked mainly in children younger than 5 years old. Moreover, deaths increased principally with age from 70 years old (Figure 3).

### Diarrheal disease burden attributable to unsafe water

**Figure 4:**
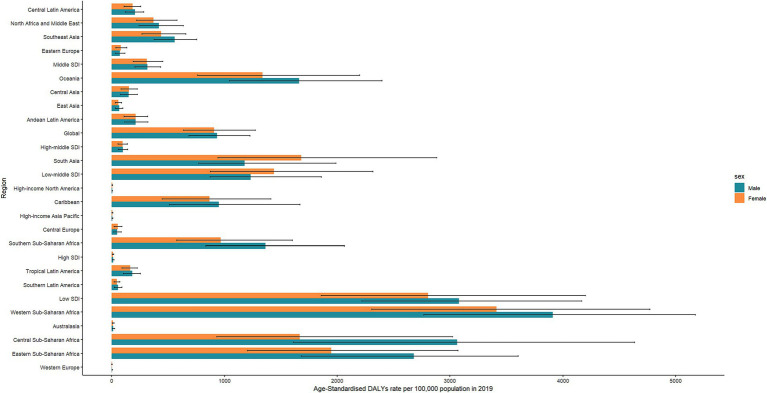
Sex-specific rates of DALYs of diarrheal diseases attributable to unsafe water in 2019 for global and regions.

**Figure 5:**
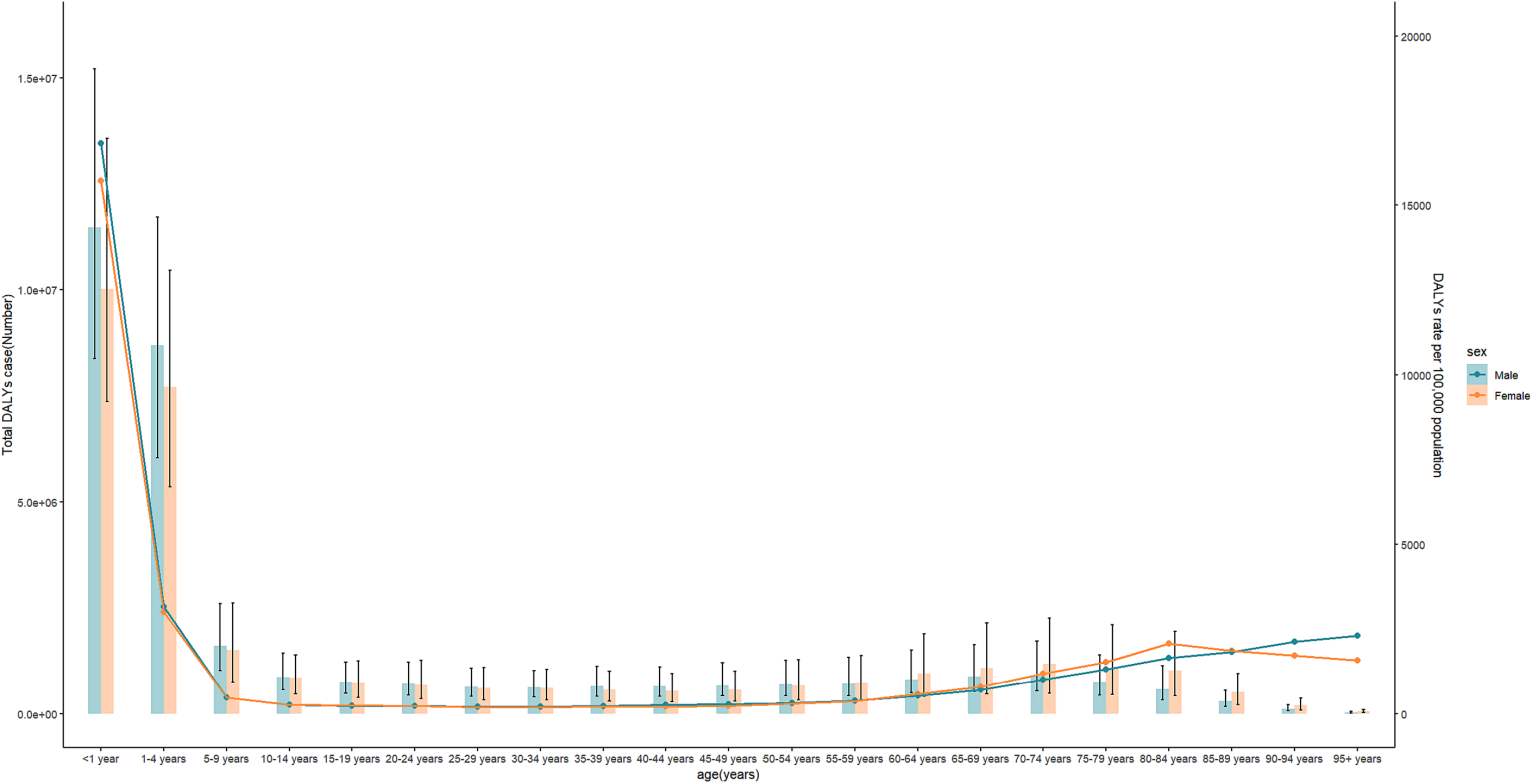
Age-specific rates of DALYs of diarrheal diseases attributable to unsafe water by sex in 2019 for global and regions.

Globally, DALYs for diarrheal diseases attributable to unsafe water decreased by 59% from 159959.8 person years (95%UI: 120917.3 to 197264.8) in 1990 to 65096.5 person years (95%UI: 47674.9 to 83747.3) in 2019 (Supplementary Table S2). Similarly, the ASR of DALYs decreased from 2707.9 (95% UI: 2032.9 to 3376.1) to 922 (95%UI: 675.8 to 1178.5), with an AAPC of −3.66 (95%CI: −3.8 to −3.52) during the study period. In 2019, the ASR of DALYs remained higher in males than in females (Figure 4). In 2019, global DALYs for diarrheal diseases attributable to unsafe water peaked in children younger than 5 years (Figure 5).

In terms of SDI, the ASR of DALYs for diarrheal diseases attributable to unsafe water showed a decreasing tendency in all SDI regions, where the AAPC ranged from −3.55 to −5.17 (Supplementary Table S2), and the highest changes were observed in the middle SDI region (AAPC: -5.17, 95%CI: −5.37 to −4.97). Additionally, the highest DALYs for diarrheal diseases attributable to unsafe water were observed in the low SDI region, with an ASR of DALYs of 2945.4 (95%UI: 2138.4 to 3867.4) in 2019. In the low-middle SDI region, DALYs were higher in males than females (Figure 4).

At the GBD regional level, Western Sub-Saharan Africa had the highest ASR of DALYs in 2019 (3,660, 95%UI: 2618.2 to 4784.9). All GBD regions showed a downward ASR of DALYs, with the highest changes in the Tropical Latin America region (AAPC: -7.78, 95%CI: −7.99 to −7.57) in the last 30 years (Supplementary Table S2). In South Asia, females had a higher burden of disease than males (Figure 4).

At the national level, the top three countries with the highest ASR of DALYs for diarrheal diseases attributable to unsafe water in 2019 continued to be the Central African Republic (8,139, 95%UI: 4781.7 to 11,786), Chad (7667.6, 95%UI: 4987.3 to 10862.2), and Niger (5213.4, 95%UI: 3234.4 to 7830.5) (Supplementary Table S3). The lowest ASR of DALYs was consistently found in Monaco in 2019 (1.4, 95%UI: 0.4 to 3.2). Since 1990, most countries (or territories) have experienced an annual decrease in the ASR of DALYs, with the fastest decrease being in Equatorial Guinea (AAPC = −9.97, 95%CI: −10.46 to −9.47). In addition, only two countries (or territories) achieved an increasing ASR of DALYs, including Puerto Rico (AAPC: 1.52, 95%CI: 1.39 to 1.65), and the Northern Mariana Islands (AAPC: 0.33, 95%CI: 0.19 to 0.47) (Supplementary Figure S1).

Additionally, the regional-level diarrheal disease-related ASR of burden attributable to unsafe water varied greatly with SDI. There was an approximately linear decreasing association between both the ASR of deaths and DALYs and SDI (Supplementary Figure S2; Figure 3); more specifically, the ASR of deaths and DALYs attributable to unsafe water gradually decreased with SDI when SDI was less than approximately 0.8 and then approached to almost zero in high SDI regions. Similar trends were observed for ASR of death and DALYs at the national level (Supplementary Figure S4; Figure 5).

## Discussion

Our study provides comprehensive estimates of the global spatial and temporal trends of the unsafe water-caused diarrheal disease burden, covering 204 countries and territories worldwide during the past 30 years. Our findings indicated that in 2019, the global number of deaths and DALYs from diarrheal diseases attributable to unsafe water nearly halved compared to 1990. Similarly, the ASR of deaths and DALYs showed only significantly decreasing trends. Additionally, the global diarrheal deaths attributable to unsafe water were greater in females than in males, which was contrary to the condition in terms of DALYs. The burden of diseases is higher in children, followed by the older adult. Furthermore, the spatiotemporal trends of the burden of diarrheal diseases attributable to unsafe water were not homogeneous, showing a negative correlation with SDI levels worldwide.

Safe drinking water remains a major global challenge, especially for underdeveloped countries and regions. According to the 2020 World Gallup Poll datasets, water insecurity should be considered when developing food and nutrition policies and interventions in low- and middle-income countries (6). Billions of people do not use safe water, and hundreds of millions do not access basic WASH services. Unsafe WASH increases the risk of diarrhea (7). A recent study using the WHO and UNICEF Joint Monitoring Programme for Water Supply, Sanitation, and Hygiene public database indicated that the proportion of diarrhea that is attributable to unsafe WASH is 0.69 (0.65–0.72), accounting for over 1 million deaths and approximately 55 million DALYs (8). Moreover, previous GBD 2019 estimated 1.7 million deaths and 88 million DALYs attributable to unsafe WASH (4). As the main risk factor for diarrheal diseases, there are no comprehensive estimates of its attributable burden. Our estimates indicated that there were 1.2 million deaths and 65 million DALYs worldwide in 2019. Interestingly, the downward trends in the absolute number of diarrheal deaths and DALYs attributable to unsafe water can be partly attributed to improved water quality, sanitation, and hygiene (“WASH”) interventions on diseases (9).

Notably, we found that global diarrheal deaths were higher in females, while the burden of diarrheal diseases attributable to unsafe water was higher in males. Diarrheal diseases are the main cause of malnutrition, and females were more likely to be malnourished or at risk of malnutrition (10). In Africa, with a higher disease burden, males experienced higher deaths and DALYs than females. Diarrhea-related deaths and DALYs attributable to unsafe water among children under 5 years were higher than any other age group, which is consistent with other reports (11–13). However, diarrhea-related deaths were also higher in the elder population aged over 60 years. More education and health promotion strategies should be conducted with both children and elders.

The disease burden varied across regions and countries and had a negative association with SDI levels. In low- and middle-income countries (LMICs), health awareness programs and safe water are lacking (14), particularly in regions with tropical climates and involving predominantly rural agricultural communities. Therefore, improvement of life and working conditions and the provision of safe water intake will play an important role in diarrheal disease prevention in this region.

Currently, fresh produce continues to be the main source of diarrheal disease outbreaks, particularly in low- and middle-income countries where water stress results in the use of surface wastewater all year round for the irrigation of vegetables (15). Local outbreaks of diarrheal disease can turn into international emergencies due to the speed and range of product distribution (16). Moreover, untreated wastewater for irrigation is another major factor that results in the contamination of food products with pathogens (17). Therefore, in order to safeguard public health and sustain livelihoods, there is a need to evaluate the microbial safety of wastewater-irrigated lettuce at locations in low- and middle-income countries.

There were some limitations in the current study. High-quality piped water that is boiled or filtered was defined in GBD 2019 as the minimum risk-exposure level for drinking water, which could lead to incorrectly estimated health effects from reporting bias (4). Second, the current findings were mainly estimated to be derived from data modeled using a Bayesian meta-regression analytical tool (DisMod-MR 2.1) in the GBD dataset rather than real observational studies, inferring that the data may be distorted. Third, we only described the burden of diarrheal disease attributable to unsafe water worldwide, which is one of the main causes of diarrheal diseases. Further comprehensive studies adjusted with multidimensional factors such as family and social assessments should be performed and provide more accurate findings.

## Conclusion

On a global scale, both the ASR of deaths and the DALYs of diarrheal disease burden attributable to unsafe water have decreased. In addition, the global burden of unsafe water exposure-related diarrheal diseases decreased from 1990 to 2019 and varied significantly according to age, sex, and region. Of note, high diarrheal diseases resulting from unsafe water occurred mainly in low SDI regions and Africa. Continued improvement in safe water intake is an urgent priority for governments, which should encourage stricter measures to provide safe water. More effort to address the current knowledge gaps and to develop effective and sustainable strategies should be tailored to the increased susceptibility of children and the older adult, as well as the particularity of LMICs, to reduce the global burden of diarrheal diseases.

## Data availability statement

The original contributions presented in the study are included in the article/Supplementary material, further inquiries can be directed to the corresponding author.

## Ethics statement

The studies involving humans were approved by Guangdong Provincial People’s Hospital. The studies were conducted in accordance with the local legislation and institutional requirements. The ethics committee/institutional review board waived the requirement of written informed consent for participation from the participants or the participants’ legal guardians/next of kin because the data was originated from public dataset.

## Author contributions

LC: Conceptualization, Data curation, Formal analysis, Software, Visualization, Writing – original draft, Writing – review & editing. JJ: Formal analysis, Methodology, Visualization, Writing – original draft, Writing – review & editing. SL: Data curation, Formal analysis, Methodology, Software, Writing – original draft. LL: Conceptualization, Data curation, Formal analysis, Software, Writing – original draft, Writing – review & editing. PL: Conceptualization, Formal analysis, Investigation, Methodology, Software, Supervision, Visualization, Writing – original draft, Writing – review & editing.

## Footnotes

^1^ https://vizhub.healthdata.org/gbd-results/

## References

[1] GBD 2016 Diarrhoeal Disease Collaborators. Estimates of the global, regional, and national morbidity, mortality, and aetiologies of diarrhoea in 195 countries: a systematic analysis for the global burden of disease study 2016. Lancet Infect Dis. (2018) 18:1211–28. doi: 10.1016/S1473-3099(18)30362-1, PMID: 30243583 PMC6202444

[2] Berendes D Fagerli K Kim S Nasrin D Powell H Kasumba I. Survey-based assessment of water, sanitation, and animal-associated risk factors for moderate-to-severe diarrhea in the vaccine impact on diarrhea in Africa (VIDA) study: the Gambia, Mali, and Kenya, 2015–2018. Clin Infect Dis. (2023) 76:S132–9. doi: 10.1093/cid/ciac911, PMID: 37074438 PMC10116493

[3] Koch T Denike K. Crediting his critics' concerns: remaking John Snow's map of broad street cholera, 1854. Soc Sci Med. (2009) 69:1246–51. doi: 10.1016/j.socscimed.2009.07.046, PMID: 19716638

[4] GBD 2019 Risk Factors Collaborators. Global burden of 87 risk factors in 204 countries and territories, 1990–2019: a systematic analysis for the global burden of disease study 2019. Lancet. (2020) 396:1223–49. doi: 10.1016/s0140-6736(20)30752-2, PMID: 33069327 PMC7566194

[5] GBD 2019 Diseases and Injuries Collaborators. Global burden of 369 diseases and injuries in 204 countries and territories, 1990–2019: a systematic analysis for the global burden of disease study 2019. Lancet. (2020) 396:1204–22. doi: 10.1016/s0140-6736(20)30925-9, PMID: 33069326 PMC7567026

[6] Young S Bethancourt H Frongillo E Viviani S Cafiero C. Concurrence of water and food insecurities, 25 low- and middle-income countries. Bull World Health Organ. (2023) 101:90–101. doi: 10.2471/blt.22.288771, PMID: 36733622 PMC9874369

[7] Wolf J Hubbard S Brauer M Ambelu A Arnold B Bain R. Effectiveness of interventions to improve drinking water, sanitation, and handwashing with soap on risk of diarrhoeal disease in children in low-income and middle-income settings: a systematic review and meta-analysis. Lancet. (2022) 400:48–59. doi: 10.1016/s0140-6736(22)00937-0, PMID: 35780792 PMC9251635

[8] Wolf J Johnston R Ambelu A Arnold B Bain R Brauer M. Burden of disease attributable to unsafe drinking water, sanitation, and hygiene in domestic settings: a global analysis for selected adverse health outcomes. Lancet. (2023) 401:2060–71. doi: 10.1016/s0140-6736(23)00458-0, PMID: 37290458 PMC10290941

[9] Clasen T Pruss-Ustun A Mathers C Cumming O Cairncross S Colford J. Estimating the impact of unsafe water, sanitation and hygiene on the global burden of disease: evolving and alternative methods. Tropical Med Int Health. (2014) 19:884–93. doi: 10.1111/tmi.12330, PMID: 24909205

[10] Carrasco C Reis G Sim-Sim M Parraca J Fernandes O Tomas-Carus P. Sex, cognitive state and falls as factors associated with malnutrition: a cross-sectional study of institutionalized older adults living in a rural area of Portugal. BMC Public Health. (2023) 21:2337. doi: 10.1186/s12889-023-15601-2, PMID: 37131189 PMC10152580

[11] Behera D Mishra S. The burden of diarrhea, etiologies, and risk factors in India from 1990 to 2019: evidence from the global burden of disease study. BMC Public Health. (2022) 22:92. doi: 10.1186/s12889-022-12515-3, PMID: 35027031 PMC8759196

[12] Local Burden of Disease Diarrhoea Collaborators. Mapping geographical inequalities in childhood diarrhoeal morbidity and mortality in low-income and middle-income countries, 2000–17: analysis for the global burden of disease study 2017. Lancet. (2020) 395:1779–801. doi: 10.1016/s0140-6736(20)30114-8, PMID: 32513411 PMC7314599

[13] GBD 2017 Diarrhoeal Disease Collaborators. Quantifying risks and interventions that have affected the burden of diarrhoea among children younger than 5 years: an analysis of the global burden of disease study 2017. Lancet Infect Dis. (2020) 20:37–59. doi: 10.1016/s1473-3099(19)30401-3, PMID: 31678029 PMC7340495

[14] Venkataramanan V Geere J Thomae B Stoler J Hunter P Young S. In pursuit of 'safe' water: the burden of personal injury from water fetching in 21 low-income and middle-income countries. BMJ Glob Health. (2020) 5:e003328. doi: 10.1136/bmjgh-2020-003328, PMID: 33115862 PMC7592242

[15] Mahami T Odai BT Nettey SNA Asamoah A Adjei I Offei B. Microbial food safety of lettuce produced under irrigated wastewater from Onyasia River in Ghana. Heliyon. (2023) 9:e19273. doi: 10.1016/j.heliyon.2023.e19273 37662740 PMC10474412

[16] Fung F Wang HS Menon S. Food safety in the 21st century. Biom J. (2018) 41:88–95. doi: 10.1016/j.bj.2018.03.003, PMID: 29866604 PMC6138766

[17] Allende A Monaghan J. Irrigation water quality for leafy crops: a perspective of risks and potential solutions. Int J Environ Res Public Health. (2015) 12:7457–77. doi: 10.3390/ijerph120707457 26151764 PMC4515668

