# Supplementary material for: Mapping the global, regional, and national burden of diarrheal diseases attributable to unsafe water
Source: Front Public Health. 2023 Dec 6;11:1302748. doi: 10.3389/fpubh.2023.1302748 (PMC10731288; doi:10.3389/fpubh.2023.1302748)

**Supplementary table 1. Deaths, age-standardised rates of Deaths per 100,000 population in 2019, and percentage change between 1990 and 2019 for diarrheal diseases resulting from unsafe water by nations**

| **location** | **1990 Counts**  **(thousand)** | **Age-standardised rate (per 100 000 population), 1990** | **Crude rate (per 100 000 population), 1990** | **2019 Counts**  **(thousand)** | **Age-standardised rate (per 100 000 population), 2019** | **Crude rate (per 100 000 population), 2019** | **Average annual percent change** | **% change in number between 1990 and 2019** |
| --- | --- | --- | --- | --- | --- | --- | --- | --- |
| Afghanistan | 3.7 (0.8 to 7.8) | 19.7 (6.4 to 38.3) | 32.4 (7 to 68.7) | 3.8 (2.1 to 6.6) | 6.6 (3.8 to 11) | 9.8 (5.4 to 17.3) | -3.44 (-4.08 to -2.81) | 0.01 (-0.55 to 2.98) |
| Albania | 0.1 (0 to 0.1) | 1.4 (0.9 to 2) | 1.6 (1 to 2.3) | 0 (0 to 0) | 0.1 (0.1 to 0.2) | 0.1 (0.1 to 0.2) | -8 (-8.71 to -7.29) | -0.93 (-0.96 to -0.88) |
| Algeria | 1.9 (0.7 to 3.8) | 6.3 (2.8 to 12.1) | 7.3 (2.8 to 14.9) | 0.3 (0.1 to 0.5) | 0.9 (0.4 to 1.5) | 0.7 (0.3 to 1.1) | -6.5 (-6.76 to -6.24) | -0.85 (-0.94 to -0.59) |
| American Samoa | 0 (0 to 0) | 11.4 (6.1 to 20.1) | 4.3 (2.5 to 7) | 0 (0 to 0) | 5.2 (2.5 to 9.3) | 3.6 (1.7 to 6.4) | -2.68 (-2.92 to -2.44) | -0.04 (-0.31 to 0.3) |
| Andorra | 0 (0 to 0) | 0 (0 to 0.1) | 0 (0 to 0) | 0 (0 to 0) | 0 (0 to 0) | 0 (0 to 0.1) | -2.23 (-2.43 to -2.03) | 1.21 (0.09 to 3.37) |
| Angola | 31.3 (12.1 to 54.6) | 299.4 (133.6 to 509) | 303.8 (117.4 to 529.2) | 11.1 (6.6 to 16.3) | 61.4 (36.9 to 97.4) | 37 (22 to 54.1) | -5.35 (-5.58 to -5.11) | -0.64 (-0.85 to 0.12) |
| Antigua and Barbuda | 0 (0 to 0) | 3.5 (2.7 to 4.3) | 3.4 (2.6 to 4.2) | 0 (0 to 0) | 1.4 (1 to 1.9) | 1.3 (0.9 to 1.6) | -3.01 (-3.29 to -2.73) | -0.47 (-0.58 to -0.34) |
| Argentina | 0.8 (0.6 to 1) | 2.6 (1.9 to 3.2) | 2.5 (1.8 to 3) | 0.3 (0.1 to 0.5) | 0.6 (0.3 to 0.9) | 0.7 (0.3 to 1.1) | -4.7 (-5.16 to -4.24) | -0.62 (-0.78 to -0.47) |
| Armenia | 0.2 (0.1 to 0.3) | 4.9 (2.2 to 7.7) | 5.3 (2.3 to 8.3) | 0 (0 to 0) | 0.1 (0 to 0.2) | 0.1 (0 to 0.2) | -12.41 (-13.05 to -11.76) | -0.98 (-0.99 to -0.98) |
| Australia | 0 (0 to 0) | 0 (0 to 0.1) | 0.1 (0 to 0.1) | 0 (0 to 0.1) | 0.1 (0 to 0.2) | 0.2 (0.1 to 0.3) | 1.87 (1.42 to 2.33) | 3.65 (1.79 to 6.45) |
| Austria | 0 (0 to 0) | 0 (0 to 0) | 0 (0 to 0) | 0 (0 to 0) | 0 (0 to 0) | 0 (0 to 0.1) | 2.66 (1.65 to 3.68) | 3.87 (1.87 to 6.7) |
| Azerbaijan | 1.1 (0.6 to 1.5) | 12.2 (7.1 to 17.1) | 14.9 (8.6 to 21) | 0.1 (0 to 0.1) | 0.8 (0.2 to 1.7) | 0.6 (0.2 to 1.2) | -8.99 (-9.36 to -8.61) | -0.94 (-0.98 to -0.89) |
| Bahamas | 0 (0 to 0) | 2.6 (1.7 to 3.4) | 1.9 (1.3 to 2.6) | 0 (0 to 0) | 0.9 (0.6 to 1.3) | 0.8 (0.5 to 1.1) | -3.52 (-4.15 to -2.87) | -0.4 (-0.56 to -0.2) |
| Bahrain | 0 (0 to 0) | 3 (1.4 to 5.5) | 0.9 (0.4 to 1.4) | 0 (0 to 0) | 1.3 (0.5 to 2.3) | 0.4 (0.2 to 0.7) | -2.91 (-3.55 to -2.26) | 0.32 (-0.18 to 1.09) |
| Bangladesh | 99.5 (71.8 to 126.9) | 144.1 (94.6 to 205.6) | 91.2 (65.9 to 116.4) | 28.2 (12.4 to 62.6) | 26.6 (11.7 to 59.2) | 17.7 (7.8 to 39.3) | -5.62 (-5.85 to -5.38) | -0.72 (-0.87 to -0.4) |
| Barbados | 0 (0 to 0) | 2 (1.3 to 2.7) | 2.2 (1.4 to 2.9) | 0 (0 to 0) | 0.8 (0.4 to 1.1) | 1 (0.6 to 1.4) | -3.33 (-3.68 to -2.98) | -0.44 (-0.59 to -0.29) |
| Belarus | 0 (0 to 0) | 0.4 (0.3 to 0.6) | 0.3 (0.2 to 0.5) | 0 (0 to 0) | 0 (0 to 0.1) | 0 (0 to 0.1) | -8.15 (-8.7 to -7.6) | -0.88 (-0.93 to -0.82) |
| Belgium | 0 (0 to 0) | 0 (0 to 0.1) | 0.1 (0 to 0.1) | 0 (0 to 0.1) | 0.1 (0 to 0.2) | 0.2 (0.1 to 0.6) | 2.18 (1.8 to 2.56) | 2.98 (1.27 to 5.67) |
| Belize | 0 (0 to 0) | 11.2 (8.9 to 13.5) | 12.2 (9.4 to 15.1) | 0 (0 to 0) | 3.1 (2.4 to 3.9) | 2.3 (1.8 to 2.9) | -4.47 (-5.18 to -3.74) | -0.58 (-0.69 to -0.44) |
| Benin | 6.5 (4.4 to 9) | 138.7 (93.5 to 196.3) | 134.6 (91.1 to 184.4) | 4.4 (2 to 8.4) | 50 (26.8 to 87.1) | 34.8 (16.1 to 66.3) | -3.52 (-3.72 to -3.31) | -0.32 (-0.66 to 0.23) |
| Bermuda | 0 (0 to 0) | 1.2 (0.9 to 1.6) | 1.1 (0.8 to 1.4) | 0 (0 to 0) | 0.3 (0.2 to 0.4) | 0.5 (0.3 to 0.7) | -5.08 (-5.22 to -4.93) | -0.52 (-0.63 to -0.37) |
| Bhutan | 0.3 (0.2 to 0.5) | 132.8 (67.3 to 217.9) | 52.1 (24.9 to 83.7) | 0.1 (0 to 0.2) | 16.4 (6.1 to 33.6) | 10 (3.7 to 19.9) | -7.01 (-7.27 to -6.74) | -0.76 (-0.89 to -0.53) |
| Bolivia (Plurinational State of) | 2.1 (1 to 3.8) | 31.3 (15.2 to 54.2) | 33.4 (15.5 to 58.5) | 0.4 (0.2 to 0.8) | 4.5 (2.2 to 8.1) | 3.7 (1.8 to 6.4) | -6.65 (-6.98 to -6.32) | -0.8 (-0.88 to -0.64) |
| Bosnia and Herzegovina | 0 (0 to 0) | 0.4 (0.2 to 0.6) | 0.3 (0.2 to 0.5) | 0 (0 to 0) | 0.1 (0 to 0.2) | 0.1 (0 to 0.2) | -5.22 (-6.05 to -4.39) | -0.81 (-0.91 to -0.66) |
| Botswana | 0.9 (0.5 to 1.3) | 101.2 (54.8 to 177.3) | 65.5 (41.2 to 99.7) | 0.4 (0.2 to 0.7) | 24.7 (11.9 to 44.4) | 17 (8.4 to 29) | -5.01 (-5.76 to -4.26) | -0.53 (-0.72 to -0.29) |
| Brazil | 33.1 (23.9 to 42.6) | 23.3 (16.9 to 29.3) | 22.2 (16 to 28.6) | 4.3 (2.6 to 5.8) | 2.2 (1.3 to 3) | 2 (1.2 to 2.7) | -7.89 (-8.15 to -7.63) | -0.87 (-0.91 to -0.83) |
| Brunei Darussalam | 0 (0 to 0) | 0.6 (0.2 to 1.3) | 0.2 (0.1 to 0.3) | 0 (0 to 0) | 0.3 (0.1 to 0.8) | 0.1 (0 to 0.3) | -2.02 (-2.61 to -1.43) | 0.24 (-0.34 to 1.16) |
| Bulgaria | 0 (0 to 0) | 0.4 (0.2 to 0.5) | 0.2 (0.1 to 0.4) | 0 (0 to 0) | 0.1 (0.1 to 0.2) | 0.1 (0.1 to 0.2) | -3.64 (-4.31 to -2.97) | -0.58 (-0.76 to -0.38) |
| Burkina Faso | 20.7 (13.7 to 28.7) | 201 (134 to 283) | 216.9 (143.1 to 299.8) | 12.9 (7.9 to 19.4) | 75.1 (45.2 to 119.6) | 57.1 (34.9 to 85.6) | -3.25 (-3.49 to -3) | -0.38 (-0.64 to 0.02) |
| Burundi | 7.8 (5 to 11.5) | 174.8 (91.6 to 287.2) | 140.6 (89.7 to 206.5) | 8.1 (3.8 to 13.6) | 89.3 (50.6 to 137.8) | 67.8 (31.7 to 114.1) | -2.32 (-2.5 to -2.14) | 0.03 (-0.52 to 0.93) |
| Cabo Verde | 0.3 (0.2 to 0.3) | 60.6 (41.6 to 82.7) | 72.2 (49.9 to 95.6) | 0 (0 to 0.1) | 8.1 (4.8 to 14.2) | 6.4 (3.8 to 11.1) | -6.4 (-7.1 to -5.69) | -0.86 (-0.91 to -0.76) |
| Cambodia | 6.3 (4 to 9.5) | 81.4 (47.1 to 134.5) | 60.3 (38.6 to 91.5) | 1.3 (0.7 to 2.3) | 13.4 (7 to 23.7) | 8 (4.4 to 13.8) | -6.07 (-6.26 to -5.89) | -0.79 (-0.88 to -0.61) |
| Cameroon | 17.8 (9.2 to 25.4) | 148.7 (88.8 to 209.5) | 171.6 (88.5 to 244.7) | 15.1 (7.4 to 24.1) | 57.8 (32 to 87) | 52 (25.3 to 82.9) | -3.2 (-3.59 to -2.82) | -0.15 (-0.49 to 0.36) |
| Canada | 0 (0 to 0) | 0 (0 to 0.1) | 0 (0 to 0.1) | 0.1 (0 to 0.1) | 0.1 (0 to 0.2) | 0.2 (0.1 to 0.4) | 3.84 (3.04 to 4.66) | 6.5 (3.4 to 11.33) |
| Central African Republic | 6.4 (3.2 to 10.7) | 261.8 (137.9 to 424.7) | 231.9 (116.3 to 391.5) | 7.4 (4.2 to 10.7) | 197.6 (108.9 to 308.3) | 139 (79.9 to 202.5) | -0.95 (-1.22 to -0.68) | 0.16 (-0.28 to 1.09) |
| Chad | 15.4 (9.9 to 21.6) | 223.3 (145 to 316.9) | 255.8 (165.1 to 358) | 26.5 (16.9 to 38.7) | 141 (93.3 to 198.7) | 161.3 (102.9 to 235.7) | -1.56 (-1.76 to -1.35) | 0.72 (0.17 to 1.51) |
| Chile | 0.2 (0.1 to 0.3) | 2.4 (1.4 to 3.3) | 1.7 (1 to 2.3) | 0.1 (0 to 0.2) | 0.5 (0.2 to 0.9) | 0.6 (0.2 to 1.1) | -5.36 (-5.7 to -5.03) | -0.5 (-0.7 to -0.28) |
| China | 75.8 (56.6 to 95.6) | 7.3 (5.4 to 9.3) | 6.4 (4.8 to 8.1) | 3.3 (1.7 to 5.4) | 0.3 (0.1 to 0.4) | 0.2 (0.1 to 0.4) | -10.84 (-11.14 to -10.54) | -0.96 (-0.97 to -0.93) |
| Colombia | 3.3 (2.2 to 4.2) | 9.6 (6.5 to 12.1) | 10 (6.8 to 12.9) | 0.5 (0.3 to 0.8) | 1.1 (0.6 to 1.6) | 1.1 (0.6 to 1.6) | -7.51 (-8.05 to -6.97) | -0.84 (-0.89 to -0.78) |
| Comoros | 0.5 (0.2 to 0.8) | 122.5 (57.9 to 201.3) | 108.1 (51.7 to 169.9) | 0.2 (0.1 to 0.3) | 43 (24.5 to 67.3) | 31.6 (18.5 to 48.9) | -3.51 (-4.36 to -2.65) | -0.55 (-0.73 to -0.04) |
| Congo | 3 (1.4 to 4.9) | 141.2 (80.8 to 218.8) | 123.9 (58 to 200.3) | 1.6 (0.7 to 2.5) | 47.8 (24 to 81) | 29.6 (14 to 47.3) | -3.59 (-3.88 to -3.31) | -0.48 (-0.71 to -0.11) |
| Cook Islands | 0 (0 to 0) | 4.3 (2.1 to 8.3) | 2.3 (1.2 to 4.4) | 0 (0 to 0) | 1.7 (0.8 to 3.1) | 2.1 (0.9 to 3.9) | -3.08 (-3.17 to -2.98) | -0.14 (-0.42 to 0.27) |
| Costa Rica | 0.1 (0.1 to 0.1) | 3.7 (2.2 to 4.9) | 2.9 (1.7 to 4) | 0.1 (0 to 0.1) | 1.1 (0.5 to 1.8) | 1.2 (0.6 to 1.9) | -3.86 (-4.52 to -3.19) | -0.38 (-0.61 to -0.13) |
| Croatia | 0 (0 to 0) | 0.2 (0.1 to 0.3) | 0.1 (0.1 to 0.2) | 0 (0 to 0) | 0.1 (0 to 0.2) | 0.2 (0.1 to 0.4) | -1.92 (-3.02 to -0.82) | 0.12 (-0.37 to 0.76) |
| Cuba | 0.4 (0.3 to 0.5) | 4 (3 to 4.7) | 3.6 (2.7 to 4.2) | 0.2 (0.1 to 0.3) | 1.1 (0.7 to 1.5) | 1.7 (1.2 to 2.3) | -4.25 (-4.83 to -3.67) | -0.48 (-0.6 to -0.35) |
| Cyprus | 0 (0 to 0) | 0.2 (0 to 0.4) | 0.1 (0 to 0.3) | 0 (0 to 0) | 0.1 (0 to 0.2) | 0.1 (0 to 0.2) | -3.54 (-4.16 to -2.91) | 0.09 (-0.6 to 1.71) |
| Czechia | 0 (0 to 0) | 0.1 (0 to 0.1) | 0.1 (0 to 0.1) | 0.1 (0 to 0.1) | 0.3 (0.1 to 0.5) | 0.5 (0.2 to 1) | 5.76 (4.6 to 6.93) | 7.17 (3.56 to 12.06) |
| C么te d'Ivoire | 10.4 (7.1 to 14.5) | 95.4 (64.3 to 135.9) | 85.4 (57.7 to 118.3) | 6.2 (3.1 to 11) | 36.2 (19.5 to 56.9) | 23.7 (11.7 to 42) | -3.23 (-3.6 to -2.87) | -0.4 (-0.66 to -0.02) |
| Democratic People's Republic of Korea | 0.6 (0.3 to 1.2) | 2.3 (1.1 to 3.9) | 3.1 (1.4 to 5.6) | 0.1 (0 to 0.2) | 0.6 (0.2 to 1.2) | 0.5 (0.2 to 0.9) | -4.49 (-4.8 to -4.19) | -0.82 (-0.94 to -0.56) |
| Democratic Republic of the Congo | 31.7 (21.8 to 43.7) | 95.4 (65.1 to 138.1) | 82.1 (56.6 to 113.2) | 29.2 (14.3 to 50.4) | 49.2 (27.6 to 83.5) | 33.3 (16.3 to 57.5) | -2.23 (-2.55 to -1.91) | -0.08 (-0.57 to 0.64) |
| Denmark | 0 (0 to 0) | 0.1 (0 to 0.1) | 0.1 (0 to 0.2) | 0 (0 to 0) | 0.1 (0 to 0.2) | 0.2 (0.1 to 0.4) | 1.03 (-0.06 to 2.14) | 1.24 (0.37 to 2.69) |
| Djibouti | 0.5 (0.3 to 0.8) | 118.3 (69.9 to 176.4) | 112.4 (56.4 to 173.5) | 0.3 (0.1 to 0.4) | 36.6 (17.1 to 61.6) | 22 (10.5 to 36.3) | -3.85 (-4.12 to -3.57) | -0.52 (-0.79 to 0.03) |
| Dominica | 0 (0 to 0) | 4.6 (2.9 to 7) | 4.7 (3 to 7.1) | 0 (0 to 0) | 1.9 (1.1 to 3) | 2 (1.1 to 3.4) | -3.06 (-3.21 to -2.91) | -0.6 (-0.71 to -0.45) |
| Dominican Republic | 2.6 (1.8 to 3.4) | 27.8 (20 to 36.3) | 35.8 (25.6 to 47.3) | 0.5 (0.2 to 0.7) | 4.7 (2.6 to 7.5) | 4.1 (2.3 to 6.7) | -6.07 (-6.73 to -5.41) | -0.83 (-0.9 to -0.72) |
| Ecuador | 2 (1.3 to 2.7) | 19.3 (11.7 to 26.9) | 20.1 (12.7 to 26.9) | 0.3 (0.1 to 0.5) | 1.9 (0.8 to 3.7) | 1.6 (0.7 to 3) | -7.99 (-8.64 to -7.33) | -0.86 (-0.93 to -0.76) |
| Egypt | 25.7 (16.2 to 35.7) | 32 (20.6 to 44) | 46.1 (29.2 to 64.1) | 3.3 (1.4 to 6) | 3.5 (1.5 to 6.2) | 3.4 (1.4 to 6) | -7.42 (-7.74 to -7.1) | -0.87 (-0.93 to -0.77) |
| El Salvador | 2 (1.4 to 2.5) | 33.9 (23.4 to 44.4) | 38 (27 to 48.4) | 0.2 (0.1 to 0.4) | 3.6 (1.8 to 6.3) | 3.5 (1.8 to 6.3) | -7.64 (-8.02 to -7.27) | -0.89 (-0.94 to -0.81) |
| Equatorial Guinea | 1.2 (0.4 to 2.1) | 284.6 (112.3 to 516.6) | 267.6 (99.5 to 491.6) | 0.1 (0 to 0.2) | 16.6 (7.4 to 31.3) | 6.8 (3.1 to 12.6) | -9.44 (-9.67 to -9.21) | -0.92 (-0.97 to -0.72) |
| Eritrea | 6.4 (2.7 to 10.5) | 342 (103.8 to 586.5) | 213.7 (90 to 349.4) | 3.6 (2 to 7) | 91.7 (45.8 to 197.4) | 54 (29.7 to 104.2) | -4.46 (-4.62 to -4.31) | -0.44 (-0.72 to 0.34) |
| Estonia | 0 (0 to 0) | 0.1 (0.1 to 0.2) | 0.1 (0.1 to 0.2) | 0 (0 to 0) | 0 (0 to 0.1) | 0.1 (0 to 0.1) | -3.85 (-5 to -2.69) | -0.51 (-0.73 to -0.26) |
| Eswatini | 0.5 (0.3 to 0.8) | 93.7 (53.8 to 150.3) | 62.4 (35.9 to 98.2) | 0.4 (0.2 to 0.7) | 52.9 (28.2 to 92.2) | 34.7 (18.8 to 57.7) | -2.06 (-2.45 to -1.67) | -0.21 (-0.48 to 0.19) |
| Ethiopia | 96.4 (62 to 134.2) | 267.6 (124.6 to 430.2) | 187.6 (120.6 to 261.1) | 43.2 (27.4 to 62.5) | 64.8 (36.6 to 97.1) | 40.1 (25.5 to 58.1) | -4.75 (-4.99 to -4.5) | -0.55 (-0.72 to -0.19) |
| Fiji | 0.1 (0.1 to 0.2) | 34.3 (18.8 to 58) | 13.6 (8.2 to 21.6) | 0.1 (0 to 0.1) | 12.6 (6.3 to 21.4) | 7.4 (3.9 to 12.1) | -3.4 (-3.64 to -3.16) | -0.35 (-0.56 to -0.02) |
| Finland | 0 (0 to 0) | 0.1 (0 to 0.1) | 0.1 (0 to 0.2) | 0 (0 to 0) | 0 (0 to 0) | 0 (0 to 0.1) | -6.32 (-6.99 to -5.65) | -0.64 (-0.79 to -0.38) |
| France | 0.1 (0 to 0.2) | 0.1 (0 to 0.3) | 0.2 (0.1 to 0.4) | 0.1 (0 to 0.2) | 0 (0 to 0.1) | 0.1 (0 to 0.3) | -4.06 (-4.39 to -3.73) | -0.27 (-0.58 to 0.21) |
| Gabon | 0.6 (0.3 to 0.9) | 70.8 (38.7 to 114.7) | 60 (32.1 to 92.6) | 0.2 (0.1 to 0.4) | 17 (7.2 to 33.9) | 11 (4.6 to 21) | -4.79 (-4.92 to -4.66) | -0.68 (-0.82 to -0.5) |
| Gambia | 0.6 (0.4 to 0.9) | 84.3 (53.1 to 127.7) | 61.4 (40.5 to 86.6) | 0.5 (0.3 to 0.7) | 35.2 (19.1 to 58) | 20.5 (11.7 to 32.2) | -2.81 (-3.34 to -2.27) | -0.24 (-0.53 to 0.15) |
| Georgia | 0.1 (0 to 0.1) | 2.1 (1.1 to 3.2) | 1.7 (0.9 to 2.5) | 0 (0 to 0) | 0.2 (0.1 to 0.3) | 0.2 (0.1 to 0.3) | -7.18 (-8.8 to -5.52) | -0.92 (-0.94 to -0.89) |
| Germany | 0 (0 to 0.1) | 0 (0 to 0) | 0 (0 to 0.1) | 0.1 (0 to 0.2) | 0 (0 to 0.1) | 0.1 (0 to 0.3) | 2.54 (1.22 to 3.87) | 2.89 (1.23 to 5.56) |
| Ghana | 22.6 (11.6 to 35.4) | 128.6 (74.6 to 190.3) | 150.6 (77.1 to 235.6) | 5.6 (3.1 to 8.5) | 26.8 (15.7 to 43.8) | 17.8 (9.9 to 27) | -5.26 (-5.73 to -4.8) | -0.75 (-0.88 to -0.47) |
| Greece | 0 (0 to 0) | 0 (0 to 0) | 0 (0 to 0) | 0 (0 to 0) | 0 (0 to 0) | 0 (0 to 0) | 1.52 (0.47 to 2.59) | 2.63 (1.19 to 4.84) |
| Greenland | 0 (0 to 0) | 0.6 (0.2 to 1.6) | 0.3 (0.1 to 0.6) | 0 (0 to 0) | 0.1 (0 to 0.4) | 0.1 (0 to 0.3) | -5.33 (-6.03 to -4.62) | -0.53 (-0.8 to 0.06) |
| Grenada | 0 (0 to 0) | 4.1 (3.2 to 5.1) | 4.2 (3.2 to 5.2) | 0 (0 to 0) | 1.1 (0.8 to 1.4) | 1 (0.7 to 1.2) | -4.4 (-4.77 to -4.03) | -0.72 (-0.79 to -0.65) |
| Guam | 0 (0 to 0) | 4.3 (2.2 to 7.9) | 1.6 (0.9 to 2.6) | 0 (0 to 0) | 2 (0.9 to 3.4) | 2.1 (1 to 3.7) | -2.71 (-3.09 to -2.32) | 0.68 (0.21 to 1.28) |
| Guatemala | 8.8 (6.9 to 10.3) | 121.8 (95.7 to 141.1) | 110.3 (86.4 to 129.1) | 2 (1.3 to 2.8) | 15.6 (10.2 to 21.4) | 11.4 (7.4 to 16) | -6.93 (-7.68 to -6.17) | -0.77 (-0.84 to -0.69) |
| Guinea | 10.3 (6.9 to 14) | 177.9 (109.4 to 264.1) | 166.1 (111.5 to 226.3) | 5.6 (3.4 to 8.3) | 65.4 (38.7 to 107.5) | 44 (26.7 to 65.4) | -3.38 (-3.66 to -3.09) | -0.46 (-0.65 to -0.18) |
| Guinea-Bissau | 1.8 (1.2 to 2.6) | 226 (126.6 to 357.1) | 182.3 (117.9 to 260.5) | 1.1 (0.6 to 1.7) | 87.8 (51.7 to 138.5) | 55.4 (30.9 to 90.9) | -2.82 (-3.32 to -2.31) | -0.43 (-0.67 to 0.02) |
| Guyana | 0.2 (0.1 to 0.2) | 26.3 (20.1 to 31.4) | 23.8 (17.9 to 29.3) | 0 (0 to 0.1) | 8.3 (6 to 11) | 6.4 (4.5 to 8.5) | -3.81 (-4.27 to -3.35) | -0.73 (-0.81 to -0.63) |
| Haiti | 8.5 (6.1 to 11.4) | 114.6 (68.6 to 174.3) | 134.3 (95.5 to 179.6) | 3.5 (1.9 to 5.5) | 30.6 (17 to 46.3) | 28.2 (15 to 44.7) | -4.45 (-5.44 to -3.45) | -0.59 (-0.76 to -0.34) |
| Honduras | 2.6 (1.8 to 3.4) | 48 (31 to 68.9) | 54.5 (37.2 to 71.7) | 0.8 (0.4 to 1.4) | 11.4 (5.4 to 21.5) | 7.9 (4 to 14) | -4.85 (-5.12 to -4.58) | -0.7 (-0.82 to -0.51) |
| Hungary | 0 (0 to 0) | 0.1 (0 to 0.1) | 0.1 (0 to 0.1) | 0.1 (0 to 0.1) | 0.3 (0.1 to 0.6) | 0.6 (0.2 to 1.1) | 6.28 (5.28 to 7.3) | 8.09 (4.24 to 12.49) |
| Iceland | 0 (0 to 0) | 0 (0 to 0) | 0 (0 to 0.1) | 0 (0 to 0) | 0 (0 to 0) | 0 (0 to 0.1) | -0.67 (-1 to -0.34) | 0.92 (0.15 to 2.22) |
| India | 915.9 (608.5 to 1266.4) | 230.5 (143.7 to 323.4) | 107 (71.1 to 148) | 508.3 (271 to 847.2) | 57.2 (30.7 to 94.2) | 36.5 (19.5 to 60.9) | -4.86 (-5.16 to -4.57) | -0.45 (-0.63 to -0.17) |
| Indonesia | 107.3 (71.9 to 149.2) | 97.8 (55.6 to 150.3) | 57.9 (38.8 to 80.5) | 45.8 (25.1 to 71) | 30.5 (16 to 47.7) | 17.6 (9.7 to 27.4) | -3.94 (-4.01 to -3.86) | -0.57 (-0.7 to -0.42) |
| Iran (Islamic Republic of) | 3.3 (1.9 to 4.8) | 5.8 (3.3 to 8.7) | 5.6 (3.2 to 8.3) | 0.5 (0.2 to 0.9) | 0.8 (0.4 to 1.4) | 0.6 (0.3 to 1) | -6.46 (-7.04 to -5.89) | -0.84 (-0.91 to -0.75) |
| Iraq | 1.9 (0.5 to 3.9) | 8.2 (3.1 to 14.8) | 11.1 (3.1 to 22.4) | 0.4 (0.2 to 0.6) | 1.3 (0.7 to 2.2) | 0.9 (0.5 to 1.4) | -5.92 (-6.76 to -5.08) | -0.8 (-0.91 to -0.34) |
| Ireland | 0 (0 to 0) | 0 (0 to 0) | 0 (0 to 0) | 0 (0 to 0) | 0 (0 to 0) | 0 (0 to 0.1) | 0.79 (-1.02 to 2.64) | 1.71 (0.51 to 3.53) |
| Israel | 0 (0 to 0) | 0.1 (0 to 0.1) | 0.1 (0 to 0.1) | 0 (0 to 0) | 0.1 (0 to 0.2) | 0.1 (0 to 0.3) | 1.18 (0.17 to 2.2) | 3.49 (1.5 to 6.78) |
| Italy | 0 (0 to 0) | 0 (0 to 0) | 0 (0 to 0) | 0 (0 to 0.1) | 0 (0 to 0.1) | 0.1 (0 to 0.2) | 1.98 (1.12 to 2.85) | 3.64 (1.93 to 6.17) |
| Jamaica | 0.2 (0.1 to 0.2) | 8.2 (6.2 to 10) | 8.2 (6.1 to 10.1) | 0 (0 to 0) | 1.1 (0.8 to 1.5) | 1.2 (0.8 to 1.6) | -6.5 (-7.6 to -5.39) | -0.83 (-0.87 to -0.79) |
| Japan | 0.2 (0.1 to 0.4) | 0.1 (0.1 to 0.3) | 0.1 (0.1 to 0.3) | 0.5 (0.2 to 1.1) | 0.1 (0 to 0.2) | 0.4 (0.2 to 0.9) | -0.87 (-1.13 to -0.61) | 1.9 (0.74 to 3.58) |
| Jordan | 0.1 (0 to 0.1) | 2.2 (1.1 to 3.7) | 1.9 (1 to 3.1) | 0 (0 to 0.1) | 0.5 (0.2 to 0.8) | 0.2 (0.1 to 0.4) | -5.2 (-5.4 to -5.01) | -0.59 (-0.8 to -0.26) |
| Kazakhstan | 1.1 (0.6 to 1.5) | 6.2 (3.4 to 8.5) | 6.6 (3.7 to 9.1) | 0 (0 to 0) | 0.2 (0.1 to 0.3) | 0.2 (0.1 to 0.3) | -11.73 (-12.26 to -11.21) | -0.97 (-0.98 to -0.96) |
| Kenya | 16.4 (12.2 to 21.6) | 93.7 (60.9 to 137.5) | 70.9 (52.4 to 93.2) | 15.4 (9.9 to 23.3) | 52.8 (29.9 to 86) | 30.7 (19.7 to 46.5) | -1.97 (-2.12 to -1.82) | -0.06 (-0.32 to 0.22) |
| Kiribati | 0.1 (0 to 0.1) | 193.5 (101 to 309.1) | 88.3 (56.1 to 129.6) | 0 (0 to 0.1) | 72.6 (35 to 125.8) | 28.2 (15.2 to 45.7) | -3.34 (-3.48 to -3.19) | -0.49 (-0.63 to -0.33) |
| Kuwait | 0 (0 to 0) | 0.4 (0.2 to 0.6) | 0.3 (0.2 to 0.5) | 0 (0 to 0) | 0.1 (0 to 0.2) | 0.1 (0 to 0.1) | -4.93 (-6.19 to -3.64) | -0.61 (-0.77 to -0.38) |
| Kyrgyzstan | 0.4 (0.3 to 0.6) | 7.1 (4.4 to 9.5) | 9.5 (5.8 to 12.8) | 0.1 (0 to 0.1) | 0.8 (0.5 to 1.2) | 0.9 (0.5 to 1.3) | -7.09 (-7.67 to -6.51) | -0.87 (-0.91 to -0.81) |
| Lao People's Democratic Republic | 4.4 (2.6 to 6.5) | 138.7 (61.6 to 235.7) | 106.3 (62.8 to 156.9) | 0.9 (0.5 to 1.5) | 21.4 (12.1 to 35.8) | 12.8 (7.1 to 20.9) | -6.17 (-6.46 to -5.89) | -0.79 (-0.88 to -0.59) |
| Latvia | 0 (0 to 0) | 0.2 (0.1 to 0.3) | 0.2 (0.1 to 0.3) | 0 (0 to 0) | 0 (0 to 0.1) | 0.1 (0 to 0.1) | -4.44 (-6.73 to -2.1) | -0.69 (-0.82 to -0.53) |
| Lebanon | 0.1 (0 to 0.1) | 2.8 (1.4 to 4.5) | 2.4 (1.3 to 3.7) | 0 (0 to 0.1) | 0.8 (0.4 to 1.5) | 0.8 (0.4 to 1.5) | -4.46 (-5.01 to -3.91) | -0.49 (-0.74 to -0.07) |
| Lesotho | 2 (1.4 to 2.8) | 140.1 (88.1 to 206.5) | 111.3 (78.3 to 153) | 1.2 (0.7 to 1.9) | 92.8 (50.1 to 157.4) | 58.2 (34 to 92.7) | -1.37 (-1.55 to -1.2) | -0.39 (-0.58 to -0.17) |
| Liberia | 3.9 (2.7 to 5.3) | 171.3 (117.1 to 237.5) | 199.6 (135 to 270.9) | 2.1 (1.1 to 3.3) | 60.5 (34.4 to 106.1) | 43 (23.7 to 68.2) | -3.42 (-3.68 to -3.16) | -0.47 (-0.7 to -0.12) |
| Libya | 0.2 (0.1 to 0.4) | 4.8 (2.1 to 8.3) | 4.8 (2.2 to 9.7) | 0 (0 to 0.1) | 0.9 (0.4 to 1.5) | 0.5 (0.2 to 0.9) | -5.62 (-6.04 to -5.2) | -0.82 (-0.91 to -0.63) |
| Lithuania | 0 (0 to 0) | 0.3 (0.1 to 0.4) | 0.2 (0.1 to 0.3) | 0 (0 to 0) | 0.1 (0 to 0.2) | 0.2 (0.1 to 0.3) | -2.47 (-3.99 to -0.93) | -0.42 (-0.65 to -0.14) |
| Luxembourg | 0 (0 to 0) | 0 (0 to 0.1) | 0.1 (0 to 0.1) | 0 (0 to 0) | 0 (0 to 0.1) | 0.1 (0 to 0.2) | -0.63 (-0.87 to -0.39) | 1.14 (0.27 to 2.51) |
| Madagascar | 19.2 (14.5 to 23.9) | 141.5 (100.7 to 195.6) | 160.4 (121.6 to 200.3) | 15.7 (10 to 22.9) | 74.7 (44.4 to 119.8) | 58.7 (37.4 to 86) | -2.09 (-2.24 to -1.94) | -0.18 (-0.44 to 0.14) |
| Malawi | 19.3 (13.4 to 25.4) | 191.1 (128.1 to 269.7) | 201.9 (139.8 to 265.4) | 6.5 (4.1 to 9.4) | 63 (36.3 to 98.8) | 35.1 (22 to 50.8) | -3.75 (-3.97 to -3.53) | -0.66 (-0.78 to -0.49) |
| Malaysia | 0.7 (0.4 to 1.2) | 7.5 (3.7 to 13.3) | 3.9 (2.1 to 6.5) | 0.7 (0.3 to 1.3) | 3.4 (1.4 to 6.4) | 2.3 (1 to 4.3) | -2.65 (-3.76 to -1.53) | 0.06 (-0.35 to 0.64) |
| Maldives | 0 (0 to 0.1) | 24.8 (16 to 38.2) | 20.8 (12.4 to 33.5) | 0 (0 to 0) | 3.2 (1.8 to 5) | 1.8 (1.1 to 2.7) | -6.86 (-7.07 to -6.64) | -0.81 (-0.9 to -0.67) |
| Mali | 20.8 (13.8 to 28.9) | 238.9 (158.9 to 329) | 240 (159.3 to 332.8) | 11.9 (6.6 to 19.1) | 86.5 (41.2 to 153.3) | 54.2 (30.1 to 87.3) | -3.39 (-3.59 to -3.18) | -0.43 (-0.66 to -0.1) |
| Malta | 0 (0 to 0) | 0 (0 to 0) | 0 (0 to 0) | 0 (0 to 0) | 0 (0 to 0) | 0 (0 to 0) | -2.15 (-2.45 to -1.86) | 0.47 (-0.19 to 1.66) |
| Marshall Islands | 0 (0 to 0) | 71.1 (35.8 to 121.9) | 25.2 (13.5 to 40.7) | 0 (0 to 0) | 18 (8.1 to 33.9) | 7.1 (3.4 to 13.2) | -4.64 (-4.8 to -4.48) | -0.65 (-0.78 to -0.45) |
| Mauritania | 2.8 (1.6 to 4.3) | 137.5 (80.6 to 203.7) | 135 (75.5 to 206.1) | 1.3 (0.6 to 2.1) | 42.4 (21.4 to 72.5) | 31.9 (15.6 to 53.4) | -3.94 (-4.1 to -3.78) | -0.54 (-0.72 to -0.26) |
| Mauritius | 0 (0 to 0) | 3.9 (1.9 to 6) | 2.7 (1.3 to 4.2) | 0 (0 to 0) | 0.5 (0.2 to 0.8) | 0.5 (0.2 to 0.9) | -7.25 (-8.46 to -6.02) | -0.8 (-0.88 to -0.71) |
| Mexico | 17.3 (11 to 22.6) | 22.7 (14.6 to 29.2) | 20.3 (12.9 to 26.4) | 2.4 (1.3 to 3.5) | 2.2 (1.2 to 3.2) | 1.9 (1 to 2.8) | -7.77 (-8.03 to -7.51) | -0.86 (-0.9 to -0.82) |
| Micronesia (Federated States of) | 0 (0 to 0) | 63.5 (29.4 to 113.4) | 26.6 (14.5 to 43.7) | 0 (0 to 0) | 14.2 (6.6 to 26.6) | 6.3 (3 to 11.6) | -5.1 (-5.91 to -4.29) | -0.77 (-0.85 to -0.64) |
| Monaco | 0 (0 to 0) | 0 (0 to 0) | 0 (0 to 0.1) | 0 (0 to 0) | 0 (0 to 0) | 0 (0 to 0) | -1.69 (-1.92 to -1.46) | -0.06 (-0.55 to 0.97) |
| Mongolia | 0.6 (0.2 to 1.1) | 17.2 (7.2 to 33.5) | 27.4 (11.3 to 53.3) | 0 (0 to 0.1) | 0.9 (0.4 to 1.6) | 1.1 (0.5 to 1.9) | -9.6 (-10.05 to -9.14) | -0.94 (-0.97 to -0.85) |
| Montenegro | 0 (0 to 0) | 0.1 (0.1 to 0.2) | 0.1 (0 to 0.1) | 0 (0 to 0) | 0 (0 to 0.1) | 0 (0 to 0.1) | -2.99 (-3.38 to -2.6) | -0.49 (-0.72 to -0.19) |
| Morocco | 10.1 (6.5 to 14.5) | 31.7 (21.3 to 44.3) | 40.1 (25.9 to 57.4) | 1 (0.5 to 1.6) | 3.5 (1.9 to 5.9) | 2.7 (1.4 to 4.5) | -7.15 (-7.26 to -7.03) | -0.91 (-0.95 to -0.82) |
| Mozambique | 24.2 (11 to 44.5) | 165.7 (83.2 to 268) | 185.3 (84 to 340.8) | 7.6 (3.7 to 12.7) | 40.8 (18.1 to 68) | 25.9 (12.6 to 43) | -4.69 (-4.91 to -4.46) | -0.68 (-0.9 to -0.03) |
| Myanmar | 44.8 (16.4 to 83.3) | 108 (46.5 to 187) | 109 (39.9 to 202.6) | 5.5 (3.4 to 8.8) | 13.8 (8.3 to 22.3) | 10.1 (6.2 to 16.1) | -6.87 (-6.98 to -6.76) | -0.88 (-0.94 to -0.65) |
| Namibia | 0.9 (0.5 to 1.4) | 97.9 (55.2 to 161.5) | 61.2 (34.7 to 96.3) | 0.6 (0.3 to 1) | 33.4 (15.6 to 60.8) | 22.9 (10.8 to 39.9) | -3.64 (-3.94 to -3.35) | -0.36 (-0.6 to 0) |
| Nauru | 0 (0 to 0) | 21.3 (11 to 39) | 7 (4 to 11.4) | 0 (0 to 0) | 8.3 (4.2 to 15.4) | 2.6 (1.4 to 4.4) | -3.14 (-3.48 to -2.79) | -0.62 (-0.73 to -0.47) |
| Nepal | 19.9 (13 to 28.5) | 145.5 (76.7 to 231.4) | 102 (66.5 to 145.8) | 4.7 (2.5 to 8.3) | 27.2 (13.6 to 50.2) | 15.5 (8.3 to 27.4) | -5.65 (-5.9 to -5.41) | -0.76 (-0.87 to -0.57) |
| Netherlands | 0 (0 to 0) | 0 (0 to 0) | 0 (0 to 0) | 0 (0 to 0) | 0 (0 to 0.1) | 0.1 (0 to 0.1) | 2.78 (2.21 to 3.35) | 3.41 (1.55 to 6.65) |
| New Zealand | 0 (0 to 0) | 0.1 (0 to 0.1) | 0.1 (0 to 0.1) | 0 (0 to 0) | 0.2 (0.1 to 0.3) | 0.3 (0.1 to 0.6) | 2.84 (1.75 to 3.94) | 4.24 (2 to 7.8) |
| Nicaragua | 1.9 (1.4 to 2.6) | 34.3 (24.6 to 44.7) | 49.9 (35.8 to 65.6) | 0.1 (0.1 to 0.2) | 2.9 (1.7 to 4.7) | 2.1 (1.2 to 3.3) | -8.17 (-8.55 to -7.79) | -0.93 (-0.96 to -0.89) |
| Niger | 31.6 (19.6 to 46) | 309.6 (186.8 to 454.2) | 393.4 (243.9 to 573.3) | 24.7 (14.4 to 38.1) | 103.7 (64.2 to 158.1) | 106.1 (62 to 163.3) | -3.66 (-3.97 to -3.34) | -0.22 (-0.59 to 0.56) |
| Nigeria | 249.8 (146.8 to 361) | 241.8 (137.1 to 348.7) | 277 (162.8 to 400.3) | 157.3 (112.1 to 205.5) | 80.1 (54.5 to 117.8) | 73.2 (52.2 to 95.6) | -3.71 (-3.95 to -3.47) | -0.37 (-0.57 to 0.03) |
| Niue | 0 (0 to 0) | 13.7 (6.6 to 26.2) | 14.7 (7.1 to 28.2) | 0 (0 to 0) | 4.5 (1.9 to 9.4) | 5.4 (2.3 to 11.4) | -3.73 (-3.85 to -3.61) | -0.73 (-0.82 to -0.62) |
| North Macedonia | 0.1 (0 to 0.1) | 5 (2.4 to 7.8) | 4.2 (2 to 6.6) | 0 (0 to 0) | 0.1 (0 to 0.2) | 0.1 (0 to 0.2) | -12.04 (-13.45 to -10.59) | -0.97 (-0.99 to -0.95) |
| Northern Mariana Islands | 0 (0 to 0) | 6.1 (2.9 to 11.2) | 1.6 (0.8 to 2.9) | 0 (0 to 0) | 4.3 (2 to 7.5) | 3.1 (1.4 to 5.4) | -1.17 (-1.32 to -1.01) | 0.8 (0.25 to 1.51) |
| Norway | 0 (0 to 0) | 0 (0 to 0.1) | 0.1 (0 to 0.1) | 0 (0 to 0) | 0.1 (0 to 0.1) | 0.1 (0 to 0.3) | 2.19 (1.49 to 2.9) | 1.89 (0.78 to 3.81) |
| Oman | 0.1 (0.1 to 0.1) | 8.7 (4.9 to 14.2) | 4.7 (2.8 to 7.4) | 0 (0 to 0) | 1.7 (0.7 to 2.8) | 0.4 (0.2 to 0.7) | -5.48 (-5.72 to -5.23) | -0.77 (-0.89 to -0.58) |
| Pakistan | 114.6 (79.2 to 155.1) | 137.6 (81.6 to 206.9) | 101.6 (70.2 to 137.4) | 65.5 (40.4 to 97.7) | 54.7 (28.5 to 91.7) | 29.2 (18 to 43.6) | -3.05 (-3.26 to -2.84) | -0.43 (-0.6 to -0.23) |
| Palau | 0 (0 to 0) | 16.9 (8 to 32.1) | 8 (4 to 14.6) | 0 (0 to 0) | 7.4 (3.3 to 14.2) | 5.3 (2.3 to 10) | -2.73 (-2.92 to -2.55) | -0.23 (-0.47 to 0.13) |
| Palestine | 0.1 (0 to 0.1) | 3.4 (1.9 to 5.5) | 2.7 (1.7 to 4) | 0 (0 to 0) | 0.8 (0.5 to 1.5) | 0.4 (0.2 to 0.7) | -4.74 (-4.9 to -4.58) | -0.62 (-0.76 to -0.42) |
| Panama | 0.2 (0.1 to 0.2) | 6.5 (4.4 to 8.6) | 6.3 (4.3 to 8.3) | 0.1 (0.1 to 0.2) | 2.7 (1.5 to 4.1) | 2.7 (1.5 to 4) | -2.96 (-3.33 to -2.58) | -0.26 (-0.49 to 0.03) |
| Papua New Guinea | 2 (1.4 to 2.7) | 92.8 (60.3 to 137.4) | 48.1 (33.2 to 66) | 2.6 (1.7 to 3.8) | 52.2 (31 to 83.1) | 26.6 (16.8 to 38.9) | -1.96 (-2.04 to -1.89) | 0.33 (-0.07 to 0.84) |
| Paraguay | 0.6 (0.4 to 0.8) | 14.8 (10 to 21.3) | 14.9 (10.6 to 19.5) | 0.1 (0.1 to 0.2) | 2 (1 to 3.4) | 1.6 (0.8 to 2.7) | -6.87 (-7.22 to -6.52) | -0.81 (-0.89 to -0.71) |
| Peru | 4.6 (2.8 to 6.9) | 18.9 (11.4 to 27.5) | 21.3 (12.8 to 31.6) | 0.7 (0.3 to 1.3) | 2.3 (1 to 3.9) | 2.2 (1 to 3.8) | -7.73 (-8.19 to -7.26) | -0.84 (-0.92 to -0.73) |
| Philippines | 19.3 (13.1 to 25.9) | 36.2 (24.1 to 53.7) | 30.5 (20.7 to 40.9) | 6.7 (4.3 to 10.1) | 8.8 (5.3 to 14.6) | 5.9 (3.8 to 9) | -4.73 (-5.1 to -4.36) | -0.66 (-0.76 to -0.49) |
| Poland | 0.1 (0 to 0.1) | 0.2 (0.1 to 0.3) | 0.1 (0.1 to 0.2) | 0.1 (0 to 0.1) | 0.1 (0 to 0.2) | 0.2 (0.1 to 0.4) | -1.79 (-2.54 to -1.05) | 0.35 (-0.2 to 0.96) |
| Portugal | 0 (0 to 0) | 0.3 (0.1 to 0.4) | 0.2 (0.1 to 0.3) | 0 (0 to 0) | 0.1 (0 to 0.2) | 0.2 (0.1 to 0.5) | -3.71 (-4.68 to -2.74) | 0.17 (-0.4 to 0.82) |
| Puerto Rico | 0 (0 to 0) | 0.4 (0.3 to 0.5) | 0.4 (0.3 to 0.5) | 0.1 (0 to 0.1) | 1 (0.6 to 1.3) | 1.9 (1.2 to 2.6) | 2.83 (2.15 to 3.51) | 3.8 (2.67 to 5.15) |
| Qatar | 0 (0 to 0) | 1.2 (0.5 to 2.3) | 0.5 (0.2 to 0.8) | 0 (0 to 0) | 0.4 (0.2 to 0.8) | 0.1 (0 to 0.1) | -3.36 (-4.31 to -2.4) | 0.15 (-0.39 to 1.1) |
| Republic of Korea | 0.2 (0.1 to 0.4) | 1 (0.3 to 2.1) | 0.4 (0.1 to 0.9) | 0.1 (0 to 0.3) | 0.2 (0 to 0.4) | 0.3 (0.1 to 0.6) | -5.64 (-5.87 to -5.4) | -0.3 (-0.63 to 0.29) |
| Republic of Moldova | 0.1 (0.1 to 0.1) | 2 (1.3 to 2.6) | 1.7 (1.1 to 2.3) | 0 (0 to 0) | 0.2 (0.1 to 0.3) | 0.1 (0.1 to 0.2) | -7.88 (-8.61 to -7.15) | -0.94 (-0.96 to -0.91) |
| Romania | 0.2 (0.2 to 0.3) | 1.5 (0.9 to 2) | 1 (0.7 to 1.4) | 0 (0 to 0.1) | 0.1 (0.1 to 0.2) | 0.2 (0.1 to 0.3) | -7.29 (-8.26 to -6.3) | -0.84 (-0.91 to -0.76) |
| Russian Federation | 0.9 (0.5 to 1.2) | 0.8 (0.5 to 1) | 0.6 (0.3 to 0.8) | 0.2 (0.1 to 0.3) | 0.1 (0.1 to 0.2) | 0.1 (0.1 to 0.2) | -6.44 (-7.26 to -5.62) | -0.82 (-0.88 to -0.76) |
| Rwanda | 13.8 (8.9 to 19.5) | 195.3 (105.8 to 314.4) | 192.4 (124.3 to 271.7) | 3.1 (1.6 to 4.9) | 38.9 (18.7 to 66.8) | 24.1 (13 to 38.7) | -4.99 (-5.41 to -4.57) | -0.78 (-0.87 to -0.63) |
| Saint Kitts and Nevis | 0 (0 to 0) | 14.8 (10.8 to 18) | 13.3 (9.6 to 16.3) | 0 (0 to 0) | 3.8 (2.5 to 5) | 3.2 (2.1 to 4.2) | -4.41 (-4.81 to -4.01) | -0.66 (-0.74 to -0.57) |
| Saint Lucia | 0 (0 to 0) | 7.2 (5.5 to 8.7) | 5.9 (4.4 to 7.2) | 0 (0 to 0) | 1.9 (1.3 to 2.5) | 1.9 (1.3 to 2.5) | -4.4 (-4.67 to -4.13) | -0.59 (-0.69 to -0.48) |
| Saint Vincent and the Grenadines | 0 (0 to 0) | 9.9 (7.5 to 12.1) | 8.5 (6.4 to 10.7) | 0 (0 to 0) | 2.8 (2 to 3.6) | 2.7 (2 to 3.5) | -4.09 (-4.54 to -3.65) | -0.67 (-0.75 to -0.57) |
| Samoa | 0 (0 to 0) | 13.2 (5.9 to 24.7) | 6.3 (3.1 to 11.1) | 0 (0 to 0) | 5.2 (2.3 to 10.1) | 3.1 (1.4 to 6.1) | -3.06 (-3.37 to -2.76) | -0.36 (-0.56 to -0.09) |
| San Marino | 0 (0 to 0) | 0 (0 to 0) | 0 (0 to 0) | 0 (0 to 0) | 0 (0 to 0) | 0 (0 to 0) | -2.35 (-2.5 to -2.2) | 0.4 (-0.28 to 1.73) |
| Sao Tome and Principe | 0.1 (0.1 to 0.1) | 67.4 (46.7 to 94.6) | 79.4 (53.2 to 111) | 0 (0 to 0) | 7.9 (3.9 to 14.1) | 4.6 (2.3 to 7.8) | -7.17 (-8.31 to -6.02) | -0.9 (-0.95 to -0.84) |
| Saudi Arabia | 1.9 (1 to 3.1) | 17.7 (9.3 to 27.6) | 11.8 (6.3 to 19.1) | 0.2 (0.1 to 0.3) | 1.3 (0.5 to 2.3) | 0.4 (0.2 to 0.7) | -8.59 (-8.79 to -8.4) | -0.92 (-0.96 to -0.86) |
| Senegal | 12.1 (8.4 to 15.9) | 165.5 (110.7 to 230.1) | 159 (109.6 to 208.8) | 5.3 (3 to 8.2) | 50.8 (28 to 80) | 35.3 (19.5 to 54.3) | -3.98 (-4.78 to -3.17) | -0.56 (-0.74 to -0.31) |
| Serbia | 0 (0 to 0) | 0.2 (0.1 to 0.3) | 0.2 (0.1 to 0.3) | 0 (0 to 0) | 0.1 (0 to 0.2) | 0.2 (0 to 0.4) | -1.68 (-2.39 to -0.96) | -0.03 (-0.7 to 1.18) |
| Seychelles | 0 (0 to 0) | 7.8 (3.7 to 13.7) | 6.1 (3 to 10.5) | 0 (0 to 0) | 3.8 (1.9 to 6.7) | 3.4 (1.7 to 6) | -2.5 (-3.08 to -1.92) | -0.21 (-0.43 to 0.1) |
| Sierra Leone | 6.8 (4.5 to 9.4) | 180.9 (117.9 to 256.6) | 187.5 (124.3 to 256) | 3.2 (1.3 to 5.9) | 68 (30.7 to 115.7) | 39.2 (15.1 to 71) | -3.28 (-3.69 to -2.86) | -0.53 (-0.81 to -0.13) |
| Singapore | 0 (0 to 0) | 0.5 (0.2 to 0.9) | 0.3 (0.1 to 0.6) | 0 (0 to 0) | 0.1 (0 to 0.2) | 0.1 (0 to 0.2) | -6.24 (-6.78 to -5.7) | -0.43 (-0.66 to -0.07) |
| Slovakia | 0 (0 to 0) | 0.1 (0.1 to 0.3) | 0.1 (0.1 to 0.3) | 0 (0 to 0) | 0.1 (0 to 0.1) | 0.1 (0 to 0.2) | -3.23 (-3.6 to -2.86) | -0.46 (-0.73 to -0.02) |
| Slovenia | 0 (0 to 0) | 0.1 (0 to 0.1) | 0.1 (0 to 0.1) | 0 (0 to 0) | 0 (0 to 0) | 0 (0 to 0.1) | -3.67 (-5.34 to -1.98) | -0.17 (-0.55 to 0.39) |
| Solomon Islands | 0.3 (0.2 to 0.4) | 193.7 (106 to 298.1) | 76.2 (45.4 to 116.2) | 0.2 (0.1 to 0.3) | 73.6 (39.9 to 123.3) | 27.8 (16.1 to 44.7) | -3.33 (-3.46 to -3.2) | -0.3 (-0.52 to 0.05) |
| Somalia | 10.1 (5.6 to 15.4) | 214.9 (89.8 to 370.3) | 141.7 (78.9 to 216) | 14.4 (8.5 to 22.2) | 111.7 (57.2 to 190.2) | 71 (41.6 to 109) | -2.22 (-2.92 to -1.52) | 0.43 (-0.09 to 1.27) |
| South Africa | 17.5 (12.2 to 24) | 54.1 (35.6 to 78) | 47.5 (33.1 to 65.2) | 9.5 (5.5 to 16) | 22.7 (12.7 to 39.3) | 17.1 (10 to 28.8) | -2.97 (-3.23 to -2.71) | -0.46 (-0.6 to -0.27) |
| South Sudan | 5.7 (3.6 to 9.3) | 134 (81 to 204.6) | 98.1 (61.4 to 159.2) | 6 (3.7 to 8.9) | 98.6 (57.4 to 150) | 64.6 (39.8 to 95.6) | -0.88 (-1.43 to -0.34) | 0.04 (-0.35 to 0.67) |
| Spain | 0 (0 to 0) | 0 (0 to 0.1) | 0 (0 to 0.1) | 0.1 (0 to 0.1) | 0 (0 to 0.1) | 0.1 (0 to 0.2) | 0.57 (0.2 to 0.93) | 2.26 (0.95 to 4.54) |
| Sri Lanka | 2.4 (1.3 to 3.5) | 27.2 (14.3 to 42) | 13.8 (7.8 to 20.6) | 0.7 (0.4 to 1.4) | 3.8 (1.8 to 6.9) | 3.4 (1.7 to 6.3) | -6.6 (-6.81 to -6.39) | -0.69 (-0.81 to -0.52) |
| Sudan | 18.8 (5.3 to 40.4) | 54.4 (19.5 to 109.2) | 93.1 (26.5 to 200.2) | 5.5 (2.4 to 10) | 14.3 (7.2 to 23.7) | 13.4 (5.9 to 24.5) | -4.49 (-4.92 to -4.06) | -0.71 (-0.85 to -0.15) |
| Suriname | 0.1 (0.1 to 0.1) | 20.6 (14.2 to 28.2) | 18.6 (13.1 to 24.5) | 0 (0 to 0) | 5.6 (3.2 to 9.2) | 5.1 (2.9 to 8.4) | -4.36 (-4.54 to -4.17) | -0.59 (-0.73 to -0.41) |
| Sweden | 0 (0 to 0) | 0 (0 to 0) | 0 (0 to 0.1) | 0 (0 to 0) | 0.1 (0 to 0.1) | 0.2 (0.1 to 0.4) | 4.82 (4.3 to 5.34) | 5.57 (2.98 to 10.04) |
| Switzerland | 0 (0 to 0) | 0 (0 to 0.1) | 0 (0 to 0.1) | 0 (0 to 0) | 0 (0 to 0.1) | 0.1 (0 to 0.1) | -0.43 (-1.18 to 0.33) | 0.9 (0.18 to 2.13) |
| Syrian Arab Republic | 0.9 (0.4 to 1.4) | 4.8 (2.7 to 7.5) | 6.8 (3.4 to 11.1) | 0.1 (0 to 0.1) | 0.7 (0.3 to 1.1) | 0.4 (0.2 to 0.8) | -6.57 (-6.94 to -6.2) | -0.93 (-0.96 to -0.85) |
| Taiwan (Province of China) | 0.1 (0 to 0.1) | 0.6 (0.4 to 0.8) | 0.4 (0.2 to 0.5) | 0 (0 to 0.1) | 0.1 (0 to 0.2) | 0.2 (0.1 to 0.3) | -5.95 (-6.89 to -5.01) | -0.56 (-0.77 to -0.32) |
| Tajikistan | 2.8 (1.9 to 3.8) | 31.1 (20.4 to 41.6) | 52.8 (34.8 to 70.8) | 0.5 (0.3 to 0.9) | 5.3 (2.7 to 8.2) | 5.8 (2.9 to 9.2) | -6.16 (-6.72 to -5.6) | -0.81 (-0.89 to -0.69) |
| Thailand | 6.3 (3.8 to 10) | 19.4 (11.2 to 33) | 11 (6.7 to 17.5) | 5 (2.6 to 8.7) | 5.4 (2.9 to 9.3) | 7.1 (3.7 to 12.4) | -4.26 (-4.41 to -4.11) | -0.2 (-0.49 to 0.21) |
| Timor-Leste | 0.5 (0.3 to 0.7) | 85.5 (43.9 to 143) | 61.7 (36.2 to 94.9) | 0.2 (0.1 to 0.3) | 22.9 (10.8 to 41.3) | 13.9 (6.4 to 23.7) | -4.52 (-4.83 to -4.2) | -0.62 (-0.79 to -0.28) |
| Togo | 6.8 (3.7 to 9.5) | 160.7 (100.9 to 225.4) | 185.7 (99.9 to 259.5) | 5 (2.5 to 7.5) | 73.4 (44.6 to 110.9) | 63.6 (31.9 to 95.1) | -2.59 (-2.81 to -2.38) | -0.26 (-0.51 to 0.11) |
| Tokelau | 0 (0 to 0) | 29.3 (14.4 to 54.2) | 23.1 (11.7 to 41.2) | 0 (0 to 0) | 6.7 (3.1 to 12.9) | 5.6 (2.7 to 10.8) | -4.97 (-5.04 to -4.9) | -0.8 (-0.85 to -0.71) |
| Tonga | 0 (0 to 0) | 8.1 (4.4 to 14.1) | 4.3 (2.6 to 7) | 0 (0 to 0) | 2.6 (1.2 to 4.9) | 2 (1 to 3.7) | -3.76 (-3.92 to -3.6) | -0.5 (-0.66 to -0.33) |
| Trinidad and Tobago | 0 (0 to 0) | 4.3 (3.1 to 5.2) | 3.2 (2.3 to 3.9) | 0 (0 to 0) | 1.2 (0.7 to 1.7) | 1.2 (0.7 to 1.7) | -4.23 (-4.72 to -3.75) | -0.56 (-0.69 to -0.41) |
| Tunisia | 0.2 (0.1 to 0.4) | 2.7 (1.4 to 4.6) | 2 (1 to 4.3) | 0.1 (0 to 0.1) | 0.7 (0.3 to 1.3) | 0.7 (0.3 to 1.2) | -4.31 (-4.45 to -4.17) | -0.56 (-0.83 to -0.2) |
| Turkey | 2.6 (1.4 to 4.3) | 4.4 (2.4 to 7.2) | 4.3 (2.3 to 7.2) | 0.5 (0.3 to 0.8) | 0.7 (0.4 to 1.1) | 0.6 (0.3 to 0.9) | -6.16 (-6.69 to -5.62) | -0.82 (-0.9 to -0.68) |
| Turkmenistan | 1 (0.7 to 1.3) | 17.3 (11.8 to 22.2) | 27.2 (18.4 to 35) | 0 (0 to 0.1) | 0.9 (0.5 to 1.4) | 0.9 (0.5 to 1.4) | -9.64 (-10.52 to -8.74) | -0.95 (-0.97 to -0.93) |
| Tuvalu | 0 (0 to 0) | 49.3 (22 to 90.6) | 27 (13.8 to 47.6) | 0 (0 to 0) | 11.2 (5.2 to 21.2) | 7.5 (3.5 to 14) | -4.96 (-5.19 to -4.73) | -0.65 (-0.75 to -0.5) |
| Uganda | 18.8 (10.7 to 29.6) | 106.6 (64.7 to 156.4) | 108.9 (61.8 to 170.9) | 9.2 (5.4 to 14.5) | 40.6 (21.3 to 68.6) | 22.4 (13.1 to 35.3) | -3.34 (-3.66 to -3.02) | -0.51 (-0.73 to -0.07) |
| Ukraine | 0.1 (0.1 to 0.2) | 0.3 (0.2 to 0.4) | 0.2 (0.1 to 0.3) | 0 (0 to 0.1) | 0.1 (0 to 0.1) | 0.1 (0.1 to 0.2) | -3.4 (-4.1 to -2.69) | -0.62 (-0.73 to -0.49) |
| United Arab Emirates | 0 (0 to 0) | 4.4 (1.3 to 8.5) | 0.8 (0.4 to 1.4) | 0 (0 to 0.1) | 1.5 (0.3 to 3.1) | 0.3 (0.1 to 0.6) | -3.81 (-4.7 to -2.91) | 0.72 (-0.13 to 1.95) |
| United Kingdom | 0 (0 to 0) | 0 (0 to 0) | 0 (0 to 0) | 0 (0 to 0.1) | 0 (0 to 0.1) | 0.1 (0 to 0.1) | 3.52 (3.07 to 3.98) | 3.8 (1.9 to 6.97) |
| United Republic of Tanzania | 28.9 (19.8 to 38.6) | 131.8 (86 to 186.9) | 111.5 (76.4 to 149.1) | 13.2 (7.7 to 21.1) | 44.1 (24.1 to 74.9) | 23.3 (13.7 to 37.2) | -3.67 (-3.91 to -3.42) | -0.54 (-0.72 to -0.31) |
| United States of America | 0.1 (0 to 0.2) | 0 (0 to 0.1) | 0 (0 to 0.1) | 0.4 (0.1 to 0.8) | 0.1 (0 to 0.1) | 0.1 (0 to 0.2) | 2.81 (2.04 to 3.59) | 3.55 (1.81 to 6.24) |
| United States Virgin Islands | 0 (0 to 0) | 1.3 (0.7 to 2.1) | 1 (0.6 to 1.6) | 0 (0 to 0) | 0.6 (0.3 to 1) | 0.8 (0.4 to 1.5) | -2.77 (-2.85 to -2.69) | -0.21 (-0.48 to 0.11) |
| Uruguay | 0.1 (0 to 0.1) | 2.2 (1.2 to 3) | 2.3 (1.3 to 3.2) | 0.1 (0 to 0.1) | 0.9 (0.4 to 1.6) | 1.6 (0.7 to 2.8) | -2.94 (-3.46 to -2.42) | -0.21 (-0.49 to 0.1) |
| Uzbekistan | 2.6 (1.4 to 3.5) | 7.9 (4.4 to 10.6) | 12.3 (6.9 to 16.7) | 0.1 (0 to 0.1) | 0.3 (0.1 to 0.5) | 0.3 (0.1 to 0.4) | -10.72 (-11.09 to -10.35) | -0.97 (-0.98 to -0.95) |
| Vanuatu | 0.1 (0 to 0.1) | 78.8 (42.1 to 130.6) | 34.2 (19 to 53.2) | 0.1 (0 to 0.1) | 39.9 (20.3 to 69.9) | 19 (10 to 31.6) | -2.34 (-2.63 to -2.04) | 0.08 (-0.26 to 0.56) |
| Venezuela (Bolivarian Republic of) | 2.6 (1.8 to 3.2) | 13.4 (9.2 to 16.5) | 13.8 (9.4 to 17) | 0.8 (0.4 to 1.2) | 3 (1.6 to 4.4) | 2.8 (1.5 to 4.1) | -4.95 (-5.88 to -4.02) | -0.7 (-0.8 to -0.59) |
| Viet Nam | 6.5 (3.7 to 10.2) | 11.9 (6.9 to 18.9) | 9.5 (5.5 to 15) | 1.3 (0.7 to 2.3) | 1.7 (0.9 to 3) | 1.3 (0.7 to 2.4) | -6.57 (-6.71 to -6.44) | -0.81 (-0.9 to -0.64) |
| Yemen | 16.4 (6.1 to 30.6) | 66.1 (30 to 115.7) | 119.8 (44.7 to 223.2) | 5.4 (2.1 to 10.3) | 15.4 (7.4 to 26.5) | 17 (6.7 to 32.6) | -4.6 (-5.28 to -3.92) | -0.67 (-0.78 to -0.49) |
| Zambia | 10.4 (6.9 to 14.9) | 147.4 (90.6 to 227) | 130.9 (87.5 to 187.1) | 5.9 (3.8 to 8.7) | 50.3 (29.5 to 81.9) | 32.4 (20.6 to 47.9) | -3.7 (-3.89 to -3.51) | -0.43 (-0.64 to -0.08) |
| Zimbabwe | 2.9 (1.8 to 4.1) | 46.6 (24.7 to 67.1) | 28.4 (17.7 to 39.6) | 3.9 (2.1 to 5.7) | 45.9 (23.4 to 68.9) | 26.1 (14.1 to 37.9) | -0.13 (-0.58 to 0.32) | 0.33 (-0.11 to 0.84) |

**Supplementary table 2. DALYs, age-standardised rates of DALYs per 100,000 population in 2019, and percentage change between 1990 and 2019 for diarrheal diseases resulting from unsafe water globally, by GBD regions and super-regions**

| **Regions** | **1990 Counts**  **(thousand)** | **Age-standardised rate (per 100 000 population), 1990** | **Crude rate (per 100 000 population), 1990** | **2019 Counts**  **(thousand)** | **Age-standardised rate (per 100 000 population), 2019** | **Crude rate (per 100 000 population), 2019** | **Average annual percent change** | **% change in number between 1990 and 2019** |
| --- | --- | --- | --- | --- | --- | --- | --- | --- |
| Global | 159959.8 (120917.3 to 197264.8) | 2707.9 (2032.9 to 3376.1) | 2990 (2260.2 to 3687.3) | 65096.5 (47674.9 to 83747.3) | 922 (675.8 to 1178.5) | 841.3 (616.2 to 1082.4) | -3.66 (-3.8 to -3.52) | -0.59 (-0.68 to -0.47) |
| High SDI | 271.4 (149.5 to 422.8) | 40.5 (22.6 to 64.7) | 33 (18.2 to 51.4) | 119.3 (51.3 to 209.1) | 11.4 (4.9 to 19.8) | 11.8 (5.1 to 20.6) | -4.26 (-4.35 to -4.18) | -0.56 (-0.73 to -0.38) |
| High-middle SDI | 4210.1 (2938.1 to 5271.9) | 401.1 (279.6 to 501.9) | 366 (255.4 to 458.3) | 1245.8 (713.7 to 1801) | 97.5 (56.6 to 139.5) | 87.1 (49.9 to 125.9) | -4.84 (-4.98 to -4.7) | -0.7 (-0.78 to -0.63) |
| Middle SDI | 26333 (19364.5 to 31826.5) | 1455.5 (1067.3 to 1784) | 1533.9 (1128 to 1853.9) | 6565.4 (4146.5 to 8982.2) | 314.5 (199.5 to 431.2) | 273.9 (173 to 374.8) | -5.17 (-5.37 to -4.97) | -0.75 (-0.8 to -0.7) |
| Low-middle SDI | 68407.5 (51080.6 to 85728) | 5408.7 (3930 to 7025.6) | 6055.6 (4521.8 to 7588.9) | 20790 (14531.6 to 28345.7) | 1342.5 (930.1 to 1866.5) | 1178.6 (823.8 to 1606.9) | -4.73 (-4.95 to -4.5) | -0.7 (-0.77 to -0.59) |
| Low SDI | 60656.8 (45434.6 to 75697.9) | 8425 (6026.4 to 10942) | 11485 (8602.7 to 14332.9) | 36339.8 (27046.5 to 46641.5) | 2945.4 (2138.4 to 3867.4) | 3219.7 (2396.3 to 4132.4) | -3.55 (-3.77 to -3.33) | -0.4 (-0.54 to -0.19) |
| Andean Latin America | 714.1 (465.5 to 935.5) | 1398.5 (920.8 to 1822.9) | 1870.6 (1219.4 to 2450.4) | 131.4 (72.3 to 193) | 212.2 (116.6 to 311.4) | 206.7 (113.7 to 303.4) | -6.67 (-7.24 to -6.09) | -0.82 (-0.87 to -0.75) |
| Australasia | 2.4 (0.9 to 4.5) | 12.3 (4.5 to 23) | 11.9 (4.4 to 22.2) | 3.6 (1.3 to 7.1) | 11.5 (4.1 to 23.1) | 12.3 (4.4 to 24.4) | -0.13 (-0.43 to 0.17) | 0.49 (0.02 to 1.12) |
| Caribbean | 1015.3 (751.2 to 1293.3) | 2466 (1825.3 to 3125.2) | 2878.4 (2129.7 to 3666.4) | 377.5 (216.8 to 587.6) | 909.1 (510.7 to 1433.5) | 800.3 (459.7 to 1245.9) | -4.13 (-5.52 to -2.72) | -0.63 (-0.75 to -0.45) |
| Central Asia | 910.4 (569.8 to 1183.8) | 985.8 (618.6 to 1280.9) | 1314.3 (822.6 to 1709) | 143.1 (77.8 to 211.5) | 152.2 (82.7 to 225) | 153 (83.2 to 226.1) | -6.37 (-6.72 to -6.02) | -0.84 (-0.88 to -0.8) |
| Central Europe | 151.8 (85.3 to 222.2) | 141 (80.3 to 202.9) | 123.5 (69.3 to 180.7) | 52 (20.8 to 88.8) | 50.9 (20.5 to 88.2) | 45.5 (18.2 to 77.7) | -3.36 (-3.57 to -3.16) | -0.66 (-0.76 to -0.58) |
| Central Latin America | 3001.1 (2096.8 to 3688.7) | 1470.5 (1030.5 to 1798.1) | 1828.6 (1277.6 to 2247.5) | 449.9 (264.7 to 626.2) | 195.6 (115 to 270.9) | 179.9 (105.9 to 250.4) | -6.78 (-6.91 to -6.65) | -0.85 (-0.88 to -0.81) |
| Central Sub-Saharan Africa | 5637.1 (3401.8 to 8288.7) | 6521.3 (4217.2 to 9330.2) | 10153.3 (6127.2 to 14929.2) | 3458.1 (1975.9 to 5422) | 2343.6 (1473.8 to 3428.9) | 2628.8 (1502.1 to 4121.8) | -3.39 (-3.56 to -3.22) | -0.39 (-0.58 to -0.14) |
| East Asia | 6754.3 (5045.4 to 8405.3) | 570.9 (425.7 to 708.4) | 551.3 (411.8 to 686.1) | 821 (449.9 to 1239.8) | 62.2 (33.9 to 93.1) | 55.8 (30.6 to 84.2) | -7.3 (-7.7 to -6.91) | -0.88 (-0.92 to -0.83) |
| Eastern Europe | 272.2 (148.9 to 404.4) | 137.5 (75.5 to 201.1) | 120.2 (65.7 to 178.6) | 148.4 (68.5 to 244.7) | 75.7 (34.6 to 125.7) | 70.7 (32.6 to 116.5) | -2.01 (-2.21 to -1.82) | -0.46 (-0.59 to -0.33) |
| Eastern Sub-Saharan Africa | 20174.7 (15464.6 to 25027.6) | 7510.9 (5361.9 to 9977.5) | 10609 (8132.1 to 13160.9) | 10047.8 (7245.9 to 13135) | 2302.7 (1580.1 to 3107.6) | 2440.1 (1759.7 to 3189.8) | -3.96 (-4.2 to -3.72) | -0.5 (-0.64 to -0.31) |
| High-income Asia Pacific | 19.6 (7.4 to 35.4) | 12.2 (4.8 to 21.8) | 11.3 (4.3 to 20.4) | 16.2 (5.8 to 31.5) | 5.5 (1.9 to 10.7) | 8.7 (3.1 to 16.8) | -2.69 (-2.89 to -2.49) | -0.17 (-0.43 to 0.15) |
| High-income North America | 29.2 (9.9 to 61) | 10.8 (3.6 to 22.5) | 10.4 (3.5 to 21.7) | 16.6 (5.5 to 34.7) | 4 (1.3 to 8.4) | 4.6 (1.5 to 9.5) | -3.49 (-3.87 to -3.11) | -0.43 (-0.63 to -0.11) |
| North Africa and Middle East | 7800.9 (5366.6 to 11095.8) | 1493.6 (1035.2 to 2103.2) | 2260.9 (1555.4 to 3215.9) | 2289.8 (1393.7 to 3368.3) | 395 (240.7 to 576.3) | 376.2 (229 to 553.3) | -4.39 (-4.65 to -4.14) | -0.71 (-0.78 to -0.62) |
| Oceania | 162.6 (116.6 to 214.8) | 2517.5 (1797.1 to 3347.9) | 2513.6 (1801.8 to 3319.6) | 193.6 (129.9 to 268.9) | 1501.7 (1011 to 2090.6) | 1458.5 (978.7 to 2025.4) | -1.8 (-1.95 to -1.66) | 0.19 (-0.17 to 0.69) |
| South Asia | 62094.9 (46474.6 to 77914.5) | 5856.3 (4148.5 to 7721) | 5657.2 (4234.1 to 7098.5) | 21267.4 (14023.4 to 30620.7) | 1432 (928.6 to 2096.7) | 1178.1 (776.8 to 1696.2) | -4.8 (-5.1 to -4.5) | -0.66 (-0.74 to -0.54) |
| Southeast Asia | 12804.1 (8662.8 to 17728.7) | 2569 (1756.1 to 3527.5) | 2743 (1855.8 to 3798) | 2806.6 (1870.8 to 3714.7) | 495.3 (330.7 to 656.9) | 416.5 (277.7 to 551.3) | -5.52 (-5.67 to -5.37) | -0.78 (-0.85 to -0.68) |
| Southern Latin America | 92 (64.1 to 114.6) | 184.7 (128.9 to 229.6) | 185.7 (129.5 to 231.2) | 32.3 (14.4 to 51.8) | 50.2 (22.4 to 80.8) | 48.4 (21.6 to 77.6) | -4.44 (-4.72 to -4.15) | -0.65 (-0.78 to -0.53) |
| Southern Sub-Saharan Africa | 1682.3 (1245.5 to 2198.6) | 2816.6 (2058.1 to 3699.6) | 3204.8 (2372.7 to 4188.4) | 844.4 (564.5 to 1177.2) | 1153.9 (757.6 to 1624.8) | 1074.6 (718.5 to 1498.2) | -3.08 (-3.58 to -2.58) | -0.5 (-0.61 to -0.37) |
| Tropical Latin America | 2868.2 (2051.3 to 3731.3) | 1736.7 (1241.5 to 2241.7) | 1876.1 (1341.7 to 2440.6) | 337.4 (189.4 to 474.1) | 173.1 (97.4 to 242) | 150.9 (84.7 to 212) | -7.78 (-7.99 to -7.57) | -0.88 (-0.92 to -0.84) |
| Western Europe | 23 (8.1 to 44.8) | 6.6 (2.3 to 12.9) | 6 (2.1 to 11.7) | 15.7 (5.1 to 32.3) | 2.9 (0.9 to 6.3) | 3.6 (1.2 to 7.4) | -2.73 (-2.86 to -2.6) | -0.32 (-0.46 to -0.18) |
| Western Sub-Saharan Africa | 33749.5 (22354.6 to 45726) | 10938.6 (7353.8 to 14742.7) | 17524.9 (11607.9 to 23743.9) | 21643.8 (15243.1 to 28796.5) | 3660 (2618.2 to 4784.9) | 4743.2 (3340.5 to 6310.7) | -3.75 (-4.05 to -3.44) | -0.36 (-0.57 to -0.01) |

DALYs: disability-adjusted life years

**Supplementary table 3. DALYs, age-standardised rates of DALYs per 100,000 population in 2019, and percentage change between 1990 and 2019 for diarrheal diseases resulting from unsafe water by nations**

| **location** | **1990 Counts**  **(thousand)** | **Age-standardised rate (per 100 000 population), 1990** | **Crude rate (per 100 000 population), 1990** | **2019 Counts**  **(thousand)** | **Age-standardised rate (per 100 000 population), 2019** | **Crude rate (per 100 000 population), 2019** | **Average annual percent change** | **% change in number between 1990 and 2019** |
| --- | --- | --- | --- | --- | --- | --- | --- | --- |
| Afghanistan | 329.4 (72.1 to 695.7) | 1569.9 (422.2 to 3176) | 2884.8 (631.7 to 6092.4) | 402.9 (248.2 to 663.7) | 674.5 (434.1 to 1060.9) | 1052.4 (648.5 to 1734) | -2.64 (-3.23 to -2.04) | 0.22 (-0.44 to 3.83) |
| Albania | 9.9 (6.2 to 13.6) | 266.2 (165.3 to 365.9) | 299.1 (187.1 to 409.4) | 2.9 (1.5 to 4.5) | 116.1 (59.3 to 181.6) | 106.3 (54 to 166.5) | -2.77 (-3.03 to -2.52) | -0.71 (-0.79 to -0.63) |
| Algeria | 179 (74.5 to 350.3) | 520.1 (227 to 990.8) | 708.2 (294.8 to 1385.6) | 50.7 (24.6 to 78.9) | 127.6 (62.2 to 197.9) | 121.1 (58.9 to 188.6) | -4.74 (-5.07 to -4.41) | -0.72 (-0.86 to -0.36) |
| American Samoa | 0.1 (0.1 to 0.2) | 345.8 (220.8 to 494.6) | 263 (173.8 to 364.5) | 0.1 (0.1 to 0.2) | 249.8 (138.7 to 363.8) | 224.7 (126 to 323.9) | -1.15 (-1.37 to -0.94) | -0.02 (-0.27 to 0.24) |
| Andorra | 0 (0 to 0) | 3.2 (0.9 to 7) | 2.6 (0.8 to 5.6) | 0 (0 to 0) | 1.7 (0.5 to 3.7) | 1.5 (0.4 to 3.2) | -2.23 (-2.3 to -2.16) | -0.12 (-0.45 to 0.5) |
| Angola | 2448.9 (886 to 4325.1) | 14688.6 (6041.9 to 25506.2) | 23733.7 (8586.3 to 41917.2) | 779.3 (471.7 to 1171) | 2329.2 (1446.3 to 3324.8) | 2585.8 (1565.2 to 3885.4) | -6.19 (-6.51 to -5.87) | -0.68 (-0.87 to 0.08) |
| Antigua and Barbuda | 0.1 (0.1 to 0.2) | 225.1 (162.5 to 285.8) | 225.5 (161.8 to 285.5) | 0.1 (0.1 to 0.2) | 174 (110 to 242.8) | 158.6 (100 to 221.1) | -0.83 (-0.95 to -0.71) | 0.02 (-0.18 to 0.22) |
| Argentina | 72.5 (53 to 89.4) | 217.9 (159.2 to 268.5) | 219 (159.9 to 269.9) | 24.5 (11 to 38.6) | 56.8 (25.3 to 89.4) | 54.4 (24.3 to 85.6) | -4.55 (-4.89 to -4.22) | -0.66 (-0.81 to -0.53) |
| Armenia | 17.8 (7.7 to 27.8) | 482.5 (209.7 to 752.5) | 522 (226.7 to 815.3) | 1.3 (0.4 to 2.6) | 47.9 (14.9 to 93.3) | 43.2 (13.5 to 84.6) | -7.8 (-8.2 to -7.4) | -0.93 (-0.96 to -0.89) |
| Australia | 1.8 (0.7 to 3.5) | 11.2 (4.1 to 21.3) | 10.8 (4 to 20.6) | 2.7 (1 to 5.7) | 10.4 (3.6 to 21.5) | 11.1 (3.9 to 23.1) | -0.15 (-0.49 to 0.19) | 0.5 (-0.05 to 1.29) |
| Austria | 0.7 (0.2 to 1.5) | 10.3 (3.4 to 21.5) | 9.4 (3.1 to 19.6) | 0.2 (0.1 to 0.4) | 1.9 (0.5 to 4.2) | 2 (0.6 to 4.5) | -5.66 (-6.02 to -5.31) | -0.75 (-0.85 to -0.62) |
| Azerbaijan | 101.5 (59.1 to 142.7) | 1136.8 (662.5 to 1593.8) | 1384.8 (805.9 to 1946.5) | 14.1 (6.6 to 23.2) | 156.5 (71.4 to 262.4) | 136.9 (63.9 to 225.7) | -6.57 (-6.98 to -6.16) | -0.86 (-0.92 to -0.79) |
| Bahamas | 0.5 (0.3 to 0.6) | 201.2 (132 to 267.2) | 182.5 (118.8 to 244.9) | 0.5 (0.3 to 0.7) | 141.1 (87.8 to 197.7) | 126.3 (77.7 to 178.2) | -1.22 (-1.37 to -1.06) | 0.02 (-0.24 to 0.26) |
| Bahrain | 0.6 (0.3 to 0.8) | 136.3 (78.1 to 202) | 112.4 (63.6 to 167) | 1.2 (0.6 to 2) | 118.7 (61.1 to 186.8) | 86.2 (44 to 137.2) | -0.45 (-0.7 to -0.2) | 1.18 (0.64 to 1.83) |
| Bangladesh | 6307.1 (4715.1 to 7825.2) | 4887.8 (3595.1 to 6226) | 5783.5 (4323.6 to 7175.5) | 984.4 (602.3 to 1720) | 725 (434 to 1299) | 618.1 (378.2 to 1080) | -6.37 (-6.52 to -6.21) | -0.84 (-0.9 to -0.73) |
| Barbados | 0.4 (0.2 to 0.5) | 158.6 (96.7 to 217.8) | 153.7 (93.2 to 209.9) | 0.4 (0.2 to 0.7) | 141.9 (77.1 to 210.6) | 148.5 (80.8 to 221.7) | -0.36 (-0.49 to -0.23) | 0.13 (-0.11 to 0.36) |
| Belarus | 11.5 (6.6 to 16.7) | 122 (71 to 177.2) | 109.9 (62.8 to 159.8) | 4.7 (1.8 to 8.4) | 51.5 (19.4 to 93.1) | 49 (18.7 to 88.2) | -2.92 (-3.1 to -2.73) | -0.6 (-0.76 to -0.45) |
| Belgium | 0.7 (0.2 to 1.5) | 7.1 (2.3 to 15) | 6.9 (2.2 to 14.6) | 0.7 (0.2 to 1.4) | 4.1 (1.2 to 9.1) | 5.9 (1.8 to 12.7) | -1.82 (-2.11 to -1.53) | -0.02 (-0.43 to 0.61) |
| Belize | 1.9 (1.5 to 2.4) | 767.1 (595.8 to 939.7) | 1019.6 (781.9 to 1274.3) | 1 (0.7 to 1.3) | 268.2 (200.4 to 344.4) | 238.3 (177.9 to 308) | -3.73 (-4.25 to -3.2) | -0.48 (-0.61 to -0.32) |
| Benin | 474.4 (309.4 to 669.8) | 6257.2 (4331.7 to 8459.3) | 9775.9 (6375.2 to 13803.1) | 304.9 (128.9 to 620.1) | 2049.1 (1056.9 to 3687) | 2407 (1017.8 to 4896.2) | -3.86 (-4.22 to -3.49) | -0.36 (-0.7 to 0.26) |
| Bermuda | 0.1 (0 to 0.1) | 129.8 (85.8 to 177.7) | 118.2 (77.7 to 161.9) | 0.1 (0.1 to 0.1) | 129.2 (76.3 to 190) | 152.5 (90.7 to 222.3) | 0 (-0.07 to 0.07) | 0.39 (0.18 to 0.59) |
| Bhutan | 17 (7.2 to 33.1) | 3303.5 (1721.8 to 5113.2) | 2779.8 (1172.6 to 5400) | 3.1 (1.6 to 5) | 504.8 (246.9 to 825.1) | 414.6 (207.3 to 669.3) | -6.43 (-6.85 to -6.02) | -0.82 (-0.91 to -0.6) |
| Bolivia (Plurinational State of) | 169.1 (73.6 to 307.5) | 1800 (863.8 to 3103.3) | 2632.9 (1145.7 to 4789) | 37.2 (19.6 to 60.1) | 293.7 (159.6 to 462.1) | 309.7 (163.1 to 500.2) | -6.11 (-6.49 to -5.73) | -0.78 (-0.86 to -0.58) |
| Bosnia and Herzegovina | 7.5 (3.9 to 11.3) | 171.6 (90.5 to 257.3) | 164.7 (86.2 to 248.2) | 1.9 (0.7 to 3.4) | 67.5 (26 to 120.5) | 57.7 (22.4 to 103.3) | -3.07 (-3.26 to -2.88) | -0.75 (-0.84 to -0.66) |
| Botswana | 53.6 (36.5 to 73.8) | 4035.8 (2594.3 to 6040.2) | 4115.8 (2801.9 to 5670.2) | 25.9 (14 to 41.9) | 1220 (665.5 to 1920.5) | 1109.5 (598.6 to 1789.6) | -4.31 (-4.53 to -4.09) | -0.52 (-0.7 to -0.21) |
| Brazil | 2821.9 (2012.2 to 3672.4) | 1769.3 (1260.9 to 2291.7) | 1896 (1352 to 2467.4) | 326.3 (183.1 to 457) | 173.2 (97.4 to 241.6) | 150.6 (84.5 to 210.9) | -7.7 (-8.04 to -7.36) | -0.88 (-0.92 to -0.84) |
| Brunei Darussalam | 0 (0 to 0.1) | 19.5 (7.9 to 36) | 13.4 (5.3 to 23.7) | 0 (0 to 0.1) | 11.5 (3.8 to 22.7) | 9.1 (3 to 17.9) | -1.81 (-2.08 to -1.54) | 0.15 (-0.33 to 0.81) |
| Bulgaria | 9.6 (4.8 to 15) | 128.7 (64.4 to 201.2) | 110.8 (54.8 to 173.3) | 3.6 (1.4 to 6.4) | 64 (24.2 to 115.6) | 52.2 (19.7 to 92.7) | -2.36 (-2.92 to -1.81) | -0.62 (-0.75 to -0.5) |
| Burkina Faso | 1579.2 (990.8 to 2212.7) | 10073.7 (6720.7 to 13713.9) | 16522.7 (10366.1 to 23150.8) | 917.8 (557 to 1396.6) | 3197.3 (2036.2 to 4705.7) | 4044.6 (2454.7 to 6154.7) | -3.83 (-4.04 to -3.62) | -0.42 (-0.68 to -0.02) |
| Burundi | 536.3 (333.7 to 777) | 7076.2 (4489.1 to 10503.2) | 9626.7 (5990.2 to 13948) | 586.1 (250.7 to 1033.9) | 3906.2 (2046.3 to 6307.6) | 4910.8 (2100.6 to 8662.9) | -2.05 (-2.25 to -1.85) | 0.09 (-0.56 to 1.22) |
| Cabo Verde | 18.8 (12.8 to 25.3) | 3748.7 (2604.1 to 4952.8) | 5340.5 (3633.6 to 7207.1) | 2 (1.4 to 2.8) | 399.9 (270.2 to 557.3) | 358.8 (242.5 to 495.4) | -7.02 (-7.7 to -6.33) | -0.89 (-0.93 to -0.83) |
| Cambodia | 437 (274.5 to 674) | 3105.7 (2009.1 to 4605.9) | 4211.5 (2645.9 to 6496.2) | 62 (38.4 to 89.6) | 438.7 (270 to 650.2) | 373.4 (231.3 to 539.9) | -6.53 (-6.77 to -6.3) | -0.86 (-0.92 to -0.76) |
| Cameroon | 1406 (679.8 to 2055.1) | 8205.3 (4451.4 to 11631.8) | 13531.5 (6542.2 to 19778.1) | 1190.8 (567.7 to 1909.8) | 3306.8 (1704.2 to 5188.2) | 4091.7 (1950.8 to 6562.4) | -3.11 (-3.65 to -2.57) | -0.15 (-0.5 to 0.4) |
| Canada | 2.7 (0.8 to 5.8) | 10.2 (3.2 to 21.6) | 9.9 (3.1 to 21.3) | 2.1 (0.6 to 4.7) | 4.9 (1.4 to 10.9) | 5.7 (1.7 to 12.8) | -2.62 (-3.19 to -2.05) | -0.23 (-0.53 to 0.31) |
| Central African Republic | 472.6 (223.5 to 816.7) | 11597.1 (6106.6 to 19409) | 17223.4 (8143.4 to 29762) | 520.9 (301.1 to 752.6) | 8139 (4781.7 to 11786) | 9828.8 (5681.5 to 14201.2) | -1.33 (-1.67 to -0.99) | 0.1 (-0.33 to 1.16) |
| Chad | 1154.2 (729 to 1661) | 11119.8 (7218.7 to 15290.2) | 19166.6 (12105.5 to 27582.1) | 2125.6 (1319.5 to 3165.3) | 7667.6 (4987.3 to 10862.2) | 12962 (8046.5 to 19301.7) | -1.3 (-1.63 to -0.97) | 0.84 (0.22 to 1.81) |
| Chile | 15.5 (8.7 to 21.9) | 121.7 (68.7 to 171.9) | 116.8 (65.4 to 165) | 5.9 (2.3 to 11.1) | 32 (12.3 to 60.3) | 32.7 (12.8 to 61.1) | -4.61 (-4.8 to -4.41) | -0.62 (-0.77 to -0.46) |
| China | 6672.4 (4974.3 to 8308.6) | 586.9 (437.4 to 729) | 563.7 (420.2 to 701.9) | 790.9 (431.5 to 1196.2) | 61.8 (33.8 to 92.7) | 55.6 (30.3 to 84.1) | -7.41 (-7.82 to -7) | -0.88 (-0.92 to -0.83) |
| Colombia | 288.6 (195.1 to 375.2) | 724.2 (489.7 to 933.5) | 886.8 (599.5 to 1152.7) | 52.4 (29 to 76) | 118.5 (65.8 to 171.7) | 109.8 (60.7 to 159) | -6.12 (-6.61 to -5.63) | -0.82 (-0.87 to -0.76) |
| Comoros | 35.2 (16.3 to 57) | 5444.6 (2571.4 to 8572.9) | 7561.2 (3503.3 to 12234.3) | 12 (7.2 to 17.9) | 1779.9 (1103.4 to 2660.6) | 1686.4 (1008.3 to 2508.8) | -3.55 (-4.23 to -2.86) | -0.66 (-0.79 to -0.28) |
| Congo | 225.2 (94.6 to 377.3) | 6461.2 (3186.6 to 10244.1) | 9212.3 (3868.3 to 15432.3) | 100 (46.5 to 149.9) | 1912.2 (970 to 2901.7) | 1899.4 (882.2 to 2846.8) | -4.06 (-4.22 to -3.89) | -0.56 (-0.72 to -0.23) |
| Cook Islands | 0 (0 to 0) | 200.3 (125.2 to 284.4) | 168.6 (104.8 to 236.4) | 0 (0 to 0.1) | 173.6 (93.4 to 260.3) | 191.5 (103.1 to 286) | -0.46 (-0.58 to -0.34) | 0.07 (-0.21 to 0.31) |
| Costa Rica | 7.7 (4.4 to 11) | 238.4 (135.7 to 334.6) | 254 (144.2 to 362.5) | 3.5 (1.6 to 5.6) | 78.7 (35.3 to 128.3) | 73.7 (33.1 to 119.4) | -3.73 (-4.09 to -3.36) | -0.55 (-0.7 to -0.4) |
| Croatia | 3.7 (1.5 to 6.3) | 83.2 (33.4 to 139.8) | 76 (29.9 to 128.2) | 1.6 (0.6 to 3) | 42.2 (14.6 to 79.6) | 37.8 (13.4 to 70.9) | -2.31 (-2.46 to -2.16) | -0.57 (-0.73 to -0.39) |
| Cuba | 25.2 (18.4 to 31.3) | 256.5 (185.8 to 318.7) | 233 (169.7 to 289.2) | 21.4 (13.5 to 30) | 162.8 (103.1 to 228.9) | 188.7 (118.8 to 263.8) | -1.51 (-1.67 to -1.36) | -0.15 (-0.29 to -0.01) |
| Cyprus | 0.1 (0 to 0.2) | 12.3 (3.9 to 26.5) | 11.5 (3.6 to 24.7) | 0.1 (0 to 0.1) | 4.6 (1.3 to 10.2) | 4.5 (1.3 to 10.1) | -3.29 (-3.87 to -2.72) | -0.34 (-0.61 to 0.04) |
| Czechia | 6.3 (2.5 to 11.5) | 64.8 (25.1 to 118.4) | 61.6 (24.1 to 111.3) | 4.1 (1.3 to 7.9) | 36.5 (11.3 to 71.4) | 38.8 (12.3 to 74.1) | -1.97 (-2.33 to -1.61) | -0.35 (-0.61 to -0.01) |
| C么te d'Ivoire | 819.4 (550.5 to 1170.6) | 4458.2 (3061.5 to 6095.3) | 6701.6 (4502.9 to 9574.7) | 444.8 (216.9 to 835.2) | 1571.7 (866.5 to 2597.2) | 1699.7 (828.9 to 3191.1) | -3.52 (-3.97 to -3.06) | -0.46 (-0.7 to -0.06) |
| Democratic People's Republic of Korea | 69.7 (35.9 to 118.8) | 242.1 (131.3 to 396.1) | 331.1 (170.5 to 564.1) | 22.4 (9.6 to 38.5) | 101.1 (42.2 to 181) | 85.4 (36.7 to 146.9) | -3 (-3.32 to -2.69) | -0.68 (-0.86 to -0.39) |
| Democratic Republic of the Congo | 2368.2 (1593.4 to 3382.2) | 4062.8 (2839.8 to 5509.3) | 6136.8 (4129 to 8764.3) | 2039.9 (895.5 to 3775) | 2090.1 (1119.5 to 3486) | 2326.8 (1021.4 to 4305.9) | -2.21 (-2.69 to -1.73) | -0.14 (-0.64 to 0.74) |
| Denmark | 0.3 (0.1 to 0.6) | 5.3 (1.6 to 12.1) | 5.4 (1.6 to 12) | 0.3 (0.1 to 0.6) | 3.3 (1 to 7.4) | 4.5 (1.4 to 9.9) | -1.66 (-1.82 to -1.49) | -0.06 (-0.41 to 0.55) |
| Djibouti | 43.9 (20.5 to 69.6) | 5900.6 (3265.8 to 8852.7) | 9026.1 (4213.4 to 14324.5) | 16.6 (8.7 to 27.3) | 1434.8 (776.8 to 2258.7) | 1381.6 (721 to 2267.8) | -4.72 (-5.14 to -4.31) | -0.62 (-0.83 to -0.17) |
| Dominica | 0.2 (0.2 to 0.3) | 287.7 (205.6 to 375.4) | 303.7 (218.2 to 394.1) | 0.1 (0.1 to 0.2) | 198.5 (130 to 271.5) | 184.6 (119.9 to 253.2) | -1.27 (-1.38 to -1.16) | -0.44 (-0.56 to -0.31) |
| Dominican Republic | 222.6 (158.4 to 297.1) | 2142.8 (1548.2 to 2810.6) | 3089.7 (2199.6 to 4124.6) | 40 (25 to 60.3) | 378.5 (238.8 to 563.5) | 367.3 (229.9 to 553.9) | -5.96 (-6.46 to -5.45) | -0.82 (-0.88 to -0.74) |
| Ecuador | 162.3 (104.4 to 213.2) | 1266.3 (807.1 to 1674.7) | 1619 (1040.8 to 2126.6) | 26 (12.8 to 39.1) | 154.4 (76.1 to 230.9) | 147.7 (72.7 to 222.6) | -7.22 (-7.74 to -6.68) | -0.84 (-0.89 to -0.78) |
| Egypt | 2264.2 (1437.9 to 3144.5) | 2629.3 (1662.5 to 3628.6) | 4065.2 (2581.6 to 5645.7) | 341.8 (148.1 to 597.4) | 336.9 (146.3 to 584.8) | 345 (149.5 to 603) | -6.89 (-7.15 to -6.63) | -0.85 (-0.92 to -0.75) |
| El Salvador | 160.9 (115.9 to 203.3) | 2307.9 (1675.8 to 2893.6) | 3054.1 (2201.1 to 3860.1) | 14.6 (9.2 to 20.5) | 245 (155.9 to 345.7) | 232.6 (147.8 to 328.2) | -7.6 (-7.96 to -7.23) | -0.91 (-0.94 to -0.87) |
| Equatorial Guinea | 83.3 (29.3 to 154.6) | 11978.3 (4752.3 to 21733) | 19363.5 (6799.8 to 35934.7) | 6.5 (3.5 to 10.9) | 587.2 (315.4 to 966.8) | 457.2 (249.2 to 764.3) | -9.97 (-10.46 to -9.47) | -0.92 (-0.97 to -0.75) |
| Eritrea | 441.7 (208.9 to 709.5) | 12647 (4973.8 to 20662.8) | 14716.3 (6961.3 to 23638.2) | 236.2 (144.1 to 398.9) | 3602.8 (2108.6 to 6683.5) | 3519.5 (2147.7 to 5943.9) | -4.23 (-4.35 to -4.11) | -0.47 (-0.73 to 0.18) |
| Estonia | 1.3 (0.6 to 2.1) | 89.4 (41.7 to 142.9) | 84.2 (39.3 to 134.8) | 0.7 (0.3 to 1.4) | 58.3 (20.8 to 109.8) | 55.6 (19.9 to 104) | -1.44 (-1.55 to -1.33) | -0.45 (-0.66 to -0.24) |
| Eswatini | 34.3 (18.8 to 56.5) | 3692.2 (2241 to 5594.3) | 4247.5 (2335.4 to 7008.1) | 25.2 (14.3 to 39.3) | 2281.2 (1305.4 to 3595.4) | 2209.9 (1252.2 to 3442.2) | -1.72 (-2.18 to -1.26) | -0.26 (-0.52 to 0.17) |
| Ethiopia | 6598.9 (4636.8 to 8603.2) | 10131.1 (6167.5 to 14619.2) | 12840.5 (9022.5 to 16740.5) | 2809.9 (1864.9 to 4018.1) | 2461.5 (1602.5 to 3452.4) | 2611.7 (1733.4 to 3734.6) | -4.69 (-4.92 to -4.47) | -0.57 (-0.72 to -0.29) |
| Fiji | 5.4 (3.6 to 7.8) | 941.5 (611 to 1393.4) | 716.4 (469.3 to 1025) | 3.9 (2.1 to 5.7) | 487.5 (266.1 to 711.4) | 426 (235.2 to 621.8) | -2.22 (-2.35 to -2.08) | -0.29 (-0.52 to 0.06) |
| Finland | 0.3 (0.1 to 0.6) | 5.4 (1.6 to 11.6) | 5.3 (1.6 to 11.2) | 0.1 (0 to 0.2) | 1.8 (0.5 to 4.3) | 1.9 (0.6 to 4.3) | -3.81 (-4.04 to -3.58) | -0.61 (-0.76 to -0.37) |
| France | 6 (1.9 to 12.8) | 10.5 (3.4 to 22.6) | 10.4 (3.3 to 22.2) | 3.1 (1 to 7.1) | 3.9 (1.2 to 9) | 4.7 (1.4 to 10.7) | -3.47 (-3.71 to -3.23) | -0.48 (-0.7 to -0.18) |
| Gabon | 38.8 (20.2 to 63) | 2955.3 (1649.2 to 4542.5) | 3911.5 (2032.7 to 6357.4) | 11.4 (5.2 to 20.2) | 708 (334.4 to 1239.4) | 653 (299.2 to 1153.8) | -4.78 (-4.94 to -4.62) | -0.71 (-0.83 to -0.51) |
| Gambia | 43.4 (27.8 to 60.8) | 3319.3 (2229.6 to 4626.7) | 4374.3 (2806.9 to 6132.3) | 28.5 (17.6 to 41.4) | 1347.7 (822.4 to 2010.1) | 1267.3 (784.8 to 1842.1) | -2.98 (-3.75 to -2.2) | -0.34 (-0.59 to 0.04) |
| Georgia | 11.4 (5.8 to 16.7) | 250.8 (127.5 to 364.9) | 207.8 (105 to 302.3) | 3.2 (1.4 to 5.4) | 99.5 (42.7 to 165.1) | 88.5 (38.4 to 146.1) | -2.82 (-3.25 to -2.39) | -0.72 (-0.8 to -0.62) |
| Germany | 3 (0.9 to 6.7) | 4 (1.2 to 8.9) | 3.8 (1.1 to 8.3) | 3.3 (1 to 7.3) | 2.7 (0.8 to 5.9) | 3.9 (1.1 to 8.6) | -1.31 (-1.5 to -1.13) | 0.09 (-0.35 to 0.78) |
| Ghana | 1820 (882.5 to 2930.8) | 7681.3 (4099.2 to 11746.3) | 12120.2 (5876.6 to 19517) | 367.6 (218.6 to 560.8) | 1204.2 (733.5 to 1765.4) | 1165.7 (693.1 to 1778.1) | -5.95 (-6.57 to -5.33) | -0.8 (-0.9 to -0.53) |
| Greece | 0.2 (0.1 to 0.5) | 2.2 (0.6 to 4.9) | 1.9 (0.6 to 4.4) | 0.2 (0.1 to 0.4) | 1.7 (0.5 to 3.6) | 1.6 (0.5 to 3.5) | -0.78 (-0.95 to -0.61) | -0.17 (-0.46 to 0.3) |
| Greenland | 0 (0 to 0) | 31.6 (11.4 to 60.6) | 27.7 (10.2 to 52.5) | 0 (0 to 0) | 8.4 (2.6 to 19.3) | 8.3 (2.6 to 18.9) | -4.45 (-4.89 to -4.02) | -0.7 (-0.83 to -0.48) |
| Grenada | 0.3 (0.2 to 0.4) | 304.4 (224 to 388) | 323.4 (237.7 to 414.8) | 0.2 (0.1 to 0.3) | 185.4 (117.5 to 259.4) | 172.7 (109.5 to 242.9) | -1.61 (-1.77 to -1.45) | -0.36 (-0.5 to -0.19) |
| Guam | 0.2 (0.1 to 0.3) | 213.9 (127 to 300.3) | 170.5 (100.6 to 240.1) | 0.4 (0.2 to 0.5) | 207.7 (107.3 to 313.1) | 212.2 (109.6 to 320.1) | -0.07 (-0.22 to 0.09) | 0.55 (0.23 to 0.81) |
| Guatemala | 624.7 (488 to 733.9) | 5880.3 (4592.5 to 6858.3) | 7841.1 (6125.9 to 9212.3) | 121.3 (79.6 to 167.5) | 708.4 (465 to 974.8) | 682.1 (448 to 942.5) | -7.08 (-7.44 to -6.72) | -0.81 (-0.86 to -0.74) |
| Guinea | 681.8 (466.2 to 925.1) | 7436.8 (5059 to 10101.2) | 11021.6 (7535.9 to 14954.4) | 344.3 (206 to 506.4) | 2508.8 (1567.1 to 3641.9) | 2723.4 (1629.7 to 4005) | -3.66 (-4.06 to -3.25) | -0.5 (-0.7 to -0.18) |
| Guinea-Bissau | 129.6 (83 to 184.4) | 9435.6 (5984.4 to 13681) | 12869.9 (8239.6 to 18311.1) | 71.1 (38.3 to 125.7) | 3546.8 (2067.7 to 5618.6) | 3742.4 (2015.3 to 6612.7) | -2.93 (-4.09 to -1.76) | -0.45 (-0.7 to 0.04) |
| Guyana | 14.1 (10.4 to 17.6) | 1546.7 (1161.7 to 1896.2) | 1831.3 (1355.5 to 2282.1) | 3.2 (2.3 to 4.3) | 464.4 (330.5 to 613.1) | 418.8 (298 to 553.2) | -4.04 (-4.41 to -3.68) | -0.77 (-0.83 to -0.69) |
| Haiti | 688.2 (480.1 to 921.9) | 6946 (4960 to 9281.4) | 10827.5 (7553.8 to 14505.4) | 282.8 (146.5 to 464.7) | 2003.4 (1087 to 3172.6) | 2280.2 (1181.5 to 3746.6) | -4.23 (-5.86 to -2.58) | -0.59 (-0.76 to -0.33) |
| Honduras | 206.6 (142.4 to 273.7) | 2878.1 (1969.1 to 3773.7) | 4388.3 (3024.2 to 5813.5) | 50.1 (28.8 to 77.1) | 529 (298 to 826.4) | 510.3 (293.1 to 785.1) | -5.67 (-5.76 to -5.58) | -0.76 (-0.84 to -0.64) |
| Hungary | 10.1 (4.4 to 16.9) | 103 (45.3 to 171.8) | 97.2 (42.2 to 162.8) | 5.2 (1.7 to 10.1) | 52 (17.3 to 102) | 53.8 (18 to 104.4) | -2.27 (-2.46 to -2.08) | -0.48 (-0.69 to -0.26) |
| Iceland | 0 (0 to 0) | 5.3 (1.5 to 11.9) | 5.2 (1.5 to 11.8) | 0 (0 to 0) | 2.2 (0.7 to 4.7) | 2.3 (0.7 to 5) | -2.94 (-3.13 to -2.75) | -0.39 (-0.63 to -0.01) |
| India | 47454.4 (34455.3 to 61786.1) | 6203.1 (4268.1 to 8387.4) | 5546.4 (4027.1 to 7221.5) | 16155.4 (10351.5 to 24036) | 1425.4 (907.6 to 2124.1) | 1161.7 (744.3 to 1728.3) | -5.01 (-5.41 to -4.62) | -0.66 (-0.75 to -0.52) |
| Indonesia | 6193.9 (4458.4 to 7940.8) | 3487.2 (2438.2 to 4679.7) | 3341.2 (2405 to 4283.6) | 1650.9 (1051.2 to 2279.2) | 848.5 (539.8 to 1157.3) | 636.3 (405.1 to 878.4) | -4.75 (-4.84 to -4.65) | -0.73 (-0.8 to -0.66) |
| Iran (Islamic Republic of) | 310 (186 to 453.4) | 404.6 (243.2 to 580) | 529.5 (317.8 to 774.6) | 99.2 (51.3 to 151.5) | 130.5 (67.9 to 198.7) | 117.7 (60.9 to 179.8) | -3.71 (-4.23 to -3.19) | -0.68 (-0.8 to -0.53) |
| Iraq | 179.8 (56.4 to 361.6) | 617 (229.5 to 1177.1) | 1021.8 (320.4 to 2054.9) | 66.8 (35.5 to 98.1) | 164.3 (86.6 to 239.2) | 158.5 (84.2 to 232.8) | -4.31 (-5.09 to -3.52) | -0.63 (-0.83 to 0.12) |
| Ireland | 0.2 (0.1 to 0.3) | 4.6 (1.4 to 10) | 4.4 (1.4 to 9.6) | 0.1 (0 to 0.2) | 1.8 (0.5 to 4.1) | 1.8 (0.5 to 4.1) | -3.19 (-3.49 to -2.89) | -0.44 (-0.66 to -0.1) |
| Israel | 0.3 (0.1 to 0.8) | 6.7 (2.1 to 15.4) | 6.8 (2.2 to 15.5) | 0.4 (0.1 to 0.9) | 3.9 (1.2 to 8.8) | 4.5 (1.4 to 10.1) | -1.94 (-2.18 to -1.71) | 0.23 (-0.29 to 1.14) |
| Italy | 2.5 (0.8 to 5.2) | 5.2 (1.6 to 11) | 4.3 (1.3 to 9.2) | 1.7 (0.6 to 3.7) | 2.8 (0.9 to 6.4) | 2.8 (0.9 to 6.1) | -2.08 (-2.26 to -1.91) | -0.32 (-0.57 to 0.07) |
| Jamaica | 14.9 (10.9 to 18.7) | 578.4 (425.1 to 722.9) | 630 (461.1 to 791.1) | 3.8 (2.5 to 5.2) | 146.8 (95.9 to 201.7) | 135.7 (88.6 to 185) | -4.64 (-5 to -4.27) | -0.74 (-0.81 to -0.67) |
| Japan | 8.4 (3 to 16.3) | 6.4 (2.3 to 12.4) | 6.7 (2.4 to 13) | 9.9 (3.5 to 19.9) | 4.5 (1.6 to 9) | 7.8 (2.8 to 15.6) | -1.26 (-1.53 to -0.99) | 0.18 (-0.28 to 0.9) |
| Jordan | 8.6 (5 to 13) | 171.3 (101 to 252.1) | 228.4 (133.4 to 345.7) | 9 (4.1 to 15.3) | 84.6 (38.1 to 142.4) | 77.7 (35.3 to 131.4) | -2.36 (-2.48 to -2.24) | 0.05 (-0.37 to 0.58) |
| Kazakhstan | 103.2 (56.4 to 141.9) | 586.5 (320.7 to 807.1) | 630.6 (344.7 to 866.7) | 13.5 (6.1 to 21.9) | 74.6 (33.7 to 120.5) | 73.6 (33.2 to 119.3) | -7.04 (-7.48 to -6.6) | -0.87 (-0.92 to -0.81) |
| Kenya | 1177.6 (875.5 to 1507.5) | 3802.1 (2797.9 to 5050.5) | 5077.2 (3774.7 to 6499.5) | 960.7 (636.2 to 1344.7) | 2033.9 (1360.8 to 2966.5) | 1912.6 (1266.6 to 2677.3) | -2.22 (-2.43 to -2.01) | -0.18 (-0.4 to 0.06) |
| Kiribati | 3.4 (2.4 to 4.6) | 5071.7 (3277.4 to 7306.4) | 4553.4 (3187 to 6243.9) | 1.5 (1 to 2.2) | 1760.7 (1039.8 to 2661.5) | 1295.9 (819.5 to 1838.5) | -3.58 (-3.69 to -3.46) | -0.54 (-0.68 to -0.36) |
| Kuwait | 1.4 (0.6 to 2.2) | 74.8 (35.3 to 119.1) | 77.3 (36.2 to 123.3) | 2.4 (1.1 to 4.2) | 67.9 (30.9 to 118.6) | 55.1 (24.9 to 95.5) | -0.26 (-0.61 to 0.1) | 0.79 (0.29 to 1.41) |
| Kyrgyzstan | 40 (24.7 to 53.4) | 670.3 (415.2 to 894.5) | 897.4 (554 to 1197.1) | 10.2 (5.5 to 14.8) | 146.9 (79.1 to 214.3) | 155.5 (83.7 to 226.8) | -5.03 (-5.74 to -4.32) | -0.75 (-0.82 to -0.67) |
| Lao People's Democratic Republic | 298.9 (185.3 to 433.1) | 5471.7 (3273.5 to 8018.5) | 7200.1 (4464.4 to 10432.5) | 49.5 (25.8 to 87) | 769.3 (426.8 to 1263.2) | 691.5 (360.5 to 1215.7) | -6.44 (-6.8 to -6.09) | -0.83 (-0.91 to -0.66) |
| Latvia | 3 (1.4 to 4.7) | 119.1 (57.7 to 186.1) | 111.9 (54.3 to 175.4) | 1.2 (0.5 to 2.1) | 65.4 (25.1 to 117.6) | 61.2 (23.5 to 110.2) | -1.93 (-2.18 to -1.68) | -0.61 (-0.75 to -0.48) |
| Lebanon | 8.1 (5 to 12.1) | 203.3 (129.1 to 292.5) | 246.3 (152.6 to 368.1) | 7.9 (4.5 to 11.6) | 153.4 (87.6 to 225.7) | 151.8 (86.6 to 223.4) | -1.09 (-1.56 to -0.62) | -0.03 (-0.34 to 0.34) |
| Lesotho | 126 (92.6 to 166.7) | 6018.1 (4326.1 to 8125.1) | 6974.5 (5126.2 to 9222.6) | 61.8 (39.9 to 90.8) | 3383.2 (2163.3 to 5038.1) | 2955.1 (1906.7 to 4342.6) | -2.02 (-2.23 to -1.8) | -0.51 (-0.66 to -0.31) |
| Liberia | 297.7 (193.7 to 411.2) | 9740.8 (6577.5 to 13234.1) | 15157.1 (9859.6 to 20933.9) | 143.7 (75.1 to 228.1) | 2819.2 (1606.2 to 4282.4) | 3001.1 (1568.7 to 4761.5) | -4.18 (-4.52 to -3.84) | -0.52 (-0.75 to -0.15) |
| Libya | 19.5 (9.8 to 37.9) | 328.9 (173.9 to 593.8) | 461 (230.3 to 893.6) | 7.7 (4 to 12.1) | 136.4 (71.1 to 214) | 113.8 (59.2 to 179.9) | -2.88 (-3.12 to -2.65) | -0.61 (-0.79 to -0.29) |
| Lithuania | 4 (2 to 6.3) | 114.1 (56.3 to 179.2) | 108.6 (53.6 to 170.8) | 1.8 (0.7 to 3.3) | 66.9 (25.5 to 123.2) | 64.6 (24.7 to 118.4) | -1.82 (-1.9 to -1.75) | -0.55 (-0.72 to -0.39) |
| Luxembourg | 0 (0 to 0) | 4.8 (1.4 to 10.9) | 4.6 (1.3 to 10.3) | 0 (0 to 0) | 2.8 (0.9 to 6.1) | 3 (0.9 to 6.5) | -1.8 (-2.04 to -1.56) | 0.07 (-0.32 to 0.75) |
| Madagascar | 1497.1 (1134.3 to 1875.7) | 7915.2 (5974.7 to 9919.1) | 12526.7 (9490.7 to 15694.3) | 1156.2 (723.1 to 1612.4) | 3669.9 (2389 to 5260.1) | 4332 (2709.2 to 6041.3) | -2.52 (-2.79 to -2.25) | -0.23 (-0.48 to 0.08) |
| Malawi | 1497.3 (1015.8 to 1997) | 9760 (6842.1 to 12856.5) | 15669.3 (10629.9 to 20898.6) | 393.6 (252.4 to 547.7) | 2307.6 (1481.9 to 3243.4) | 2134.1 (1368.7 to 2969.8) | -4.94 (-5.22 to -4.66) | -0.74 (-0.83 to -0.59) |
| Malaysia | 37.4 (21.7 to 55.1) | 246.1 (137.5 to 371.1) | 212 (122.7 to 311.9) | 36.5 (18 to 56) | 136.9 (67.7 to 211.3) | 116.5 (57.5 to 178.9) | -1.95 (-2.34 to -1.55) | -0.03 (-0.31 to 0.3) |
| Maldives | 3.7 (2.2 to 6) | 1151.3 (735.7 to 1758.9) | 1676.2 (982 to 2711.7) | 0.7 (0.5 to 1) | 187.2 (128.6 to 244.5) | 145.4 (99.5 to 191.6) | -6.1 (-6.33 to -5.86) | -0.81 (-0.89 to -0.65) |
| Mali | 1521.5 (963.4 to 2199.6) | 10996.2 (7413.2 to 15112.7) | 17543.7 (11108.2 to 25361.6) | 754.9 (476 to 1126.8) | 3103.6 (1773.7 to 4966.5) | 3444.1 (2171.9 to 5141.2) | -4.19 (-4.45 to -3.94) | -0.5 (-0.7 to -0.21) |
| Malta | 0 (0 to 0) | 6.3 (2 to 13.9) | 5.9 (1.8 to 12.7) | 0 (0 to 0) | 1.9 (0.6 to 4.4) | 1.9 (0.6 to 4.3) | -4.05 (-4.24 to -3.86) | -0.61 (-0.77 to -0.35) |
| Marshall Islands | 0.6 (0.3 to 1) | 1754.1 (1019.3 to 2752) | 1342.8 (722.4 to 2167.1) | 0.2 (0.1 to 0.4) | 554.6 (326.6 to 869.5) | 415.1 (248.6 to 632.6) | -4.45 (-4.91 to -3.98) | -0.62 (-0.75 to -0.37) |
| Mauritania | 201.3 (103.3 to 324.6) | 6551.5 (3770.4 to 9868.7) | 9739.2 (5000.1 to 15710.6) | 87.6 (41.1 to 142.7) | 2057 (1073 to 3300.7) | 2181.3 (1022.8 to 3554) | -3.84 (-4.06 to -3.63) | -0.56 (-0.74 to -0.26) |
| Mauritius | 2.1 (1 to 3.2) | 212.3 (101.5 to 328.1) | 188.4 (89.9 to 292.6) | 0.5 (0.1 to 0.9) | 41.2 (12.8 to 78.6) | 37.2 (11.5 to 69.2) | -5.48 (-6.34 to -4.62) | -0.77 (-0.87 to -0.67) |
| Mexico | 1310.4 (824.6 to 1729.4) | 1267.2 (807.3 to 1658.3) | 1532.9 (964.6 to 2023) | 134.8 (69.2 to 196.8) | 119.4 (61.7 to 175.8) | 107.9 (55.4 to 157.5) | -7.91 (-8.16 to -7.66) | -0.9 (-0.93 to -0.87) |
| Micronesia (Federated States of) | 1.5 (0.8 to 2.4) | 1644.1 (966.6 to 2613.6) | 1407.7 (813.2 to 2261.4) | 0.4 (0.2 to 0.5) | 450.8 (270.1 to 692.8) | 344.5 (213.5 to 502.8) | -4.53 (-5.77 to -3.27) | -0.76 (-0.85 to -0.63) |
| Monaco | 0 (0 to 0) | 2.7 (0.8 to 6.2) | 2.6 (0.8 to 6) | 0 (0 to 0) | 1.4 (0.4 to 3.2) | 1.5 (0.4 to 3.3) | -2.18 (-2.29 to -2.08) | -0.31 (-0.59 to 0.11) |
| Mongolia | 54.5 (23.8 to 103.9) | 1611.4 (708.5 to 3054.1) | 2531 (1103.3 to 4824.4) | 7 (4.1 to 10.3) | 193.3 (113 to 279.9) | 207.8 (120.8 to 303.6) | -7.03 (-7.39 to -6.66) | -0.87 (-0.94 to -0.72) |
| Montenegro | 0.6 (0.3 to 1) | 104.3 (53.8 to 161) | 102.2 (52.8 to 157.5) | 0.4 (0.2 to 0.6) | 69.9 (32.9 to 112.7) | 62.9 (29.9 to 100.5) | -1.39 (-1.47 to -1.31) | -0.39 (-0.54 to -0.22) |
| Morocco | 890.1 (578 to 1275.6) | 2500.3 (1627.7 to 3553.3) | 3518.5 (2285 to 5042.7) | 104.2 (60.8 to 161.4) | 336.6 (195.8 to 525.5) | 290 (169 to 448.8) | -6.65 (-6.76 to -6.55) | -0.88 (-0.93 to -0.79) |
| Mozambique | 1895.2 (808.3 to 3654.1) | 9063.1 (4288.7 to 16431.9) | 14499 (6183.9 to 27956) | 522.7 (270.8 to 868.5) | 1634.6 (844.2 to 2617.9) | 1770.1 (917.1 to 2941.2) | -5.84 (-6.32 to -5.37) | -0.72 (-0.9 to -0.11) |
| Myanmar | 3463 (1092.3 to 6728) | 6327.4 (2291.8 to 11813.3) | 8424.9 (2657.3 to 16368.3) | 274.9 (178.7 to 391.6) | 569.2 (371.6 to 805.1) | 502.7 (326.8 to 716.2) | -8.06 (-8.26 to -7.87) | -0.92 (-0.96 to -0.73) |
| Namibia | 46.5 (26.5 to 74.1) | 3370.4 (1972.9 to 5140.1) | 3297.3 (1880.3 to 5258.9) | 30.7 (16.6 to 50.5) | 1353.3 (722.1 to 2201.9) | 1278.5 (692.5 to 2102.6) | -3.12 (-3.46 to -2.77) | -0.34 (-0.57 to 0.05) |
| Nauru | 0 (0 to 0.1) | 598.3 (376.5 to 914) | 472.4 (306.1 to 700.5) | 0 (0 to 0) | 353.5 (211.7 to 526) | 274 (168.2 to 395.3) | -1.71 (-2.01 to -1.42) | -0.4 (-0.58 to -0.19) |
| Nepal | 1354.4 (919.8 to 1926.1) | 5321 (3528 to 7518.3) | 6932.7 (4708.2 to 9858.9) | 198 (134.2 to 275.8) | 787.1 (512.9 to 1133.4) | 650.8 (441.3 to 906.8) | -6.43 (-6.84 to -6.03) | -0.85 (-0.91 to -0.77) |
| Netherlands | 0.4 (0.1 to 1) | 3.1 (0.9 to 7) | 2.8 (0.9 to 6.4) | 0.4 (0.1 to 0.8) | 1.8 (0.5 to 4.1) | 2.1 (0.7 to 4.9) | -1.89 (-2.09 to -1.68) | -0.14 (-0.46 to 0.4) |
| New Zealand | 0.6 (0.2 to 1.2) | 17.5 (5.9 to 35.9) | 17 (5.8 to 34.9) | 0.9 (0.3 to 1.7) | 17.4 (5.9 to 34.8) | 18.9 (6.4 to 38.1) | -0.04 (-0.45 to 0.38) | 0.46 (-0.13 to 1.3) |
| Nicaragua | 170.5 (122.3 to 223.7) | 2669.5 (1925.1 to 3471.4) | 4387.2 (3146.2 to 5755.9) | 13.4 (8.3 to 18.6) | 219.7 (136.2 to 302.7) | 206.4 (128 to 286.2) | -8.28 (-8.63 to -7.93) | -0.92 (-0.95 to -0.89) |
| Niger | 2568.2 (1570.7 to 3763.6) | 17426.2 (10813.4 to 25096.6) | 32010.5 (19578.1 to 46910.5) | 1966.8 (1118.1 to 3073.1) | 5213.4 (3234.4 to 7830.5) | 8442.9 (4799.5 to 13192.1) | -3.97 (-4.4 to -3.55) | -0.23 (-0.61 to 0.56) |
| Nigeria | 19089.2 (11379 to 28031.1) | 13350.9 (7912.8 to 19321.2) | 21166.3 (12617.1 to 31081.1) | 11980 (8705 to 15996.9) | 4239.8 (3078.7 to 5501.5) | 5576.7 (4052.1 to 7446.5) | -3.83 (-3.99 to -3.67) | -0.37 (-0.59 to 0.06) |
| Niue | 0 (0 to 0) | 426.6 (262.2 to 663.5) | 439.7 (274.1 to 686.3) | 0 (0 to 0) | 263.9 (142.7 to 396.5) | 283.3 (153.1 to 426.1) | -1.62 (-1.71 to -1.54) | -0.54 (-0.66 to -0.39) |
| North Macedonia | 9.3 (4.5 to 14.3) | 524.6 (256.1 to 807.6) | 460.7 (224.4 to 709.8) | 1.2 (0.5 to 2.1) | 62.4 (24.1 to 112.3) | 54.8 (21.2 to 98.6) | -7.07 (-7.64 to -6.5) | -0.87 (-0.93 to -0.8) |
| Northern Mariana Islands | 0.1 (0 to 0.1) | 234.9 (137.6 to 340.6) | 156.7 (94.7 to 222.9) | 0.1 (0.1 to 0.2) | 254.5 (137.6 to 368.6) | 256.6 (139.5 to 376.1) | 0.33 (0.19 to 0.47) | 0.53 (0.23 to 0.82) |
| Norway | 0.2 (0.1 to 0.4) | 4 (1.2 to 8.9) | 4.3 (1.3 to 9.6) | 0.2 (0.1 to 0.4) | 2.3 (0.7 to 5.1) | 3.2 (1 to 6.9) | -1.86 (-2.03 to -1.69) | -0.08 (-0.43 to 0.53) |
| Oman | 8.4 (5.5 to 12.9) | 387.4 (257.2 to 552) | 432.4 (282.2 to 662.3) | 5.5 (2.9 to 8.5) | 157.3 (82 to 241.6) | 119.7 (62.8 to 185.4) | -3.07 (-3.23 to -2.92) | -0.35 (-0.65 to 0.11) |
| Pakistan | 6962 (5023.8 to 8971.5) | 4728.5 (3349.7 to 6326.6) | 6170 (4452.3 to 7950.8) | 3926.5 (2702.4 to 5432.3) | 1801.3 (1197.8 to 2547.8) | 1752.4 (1206.1 to 2424.5) | -3.16 (-3.41 to -2.92) | -0.44 (-0.61 to -0.21) |
| Palau | 0.1 (0 to 0.1) | 476.7 (289.7 to 723.2) | 362.6 (226 to 525.8) | 0.1 (0 to 0.1) | 316.8 (177 to 471) | 311 (171.1 to 462.7) | -1.41 (-1.51 to -1.31) | 0 (-0.28 to 0.29) |
| Palestine | 6.3 (4.3 to 8.7) | 215.1 (148 to 285.5) | 303.6 (209.2 to 421.3) | 6.4 (3.9 to 9.3) | 133.5 (81.2 to 189.9) | 129.8 (78.2 to 186.8) | -1.59 (-1.73 to -1.45) | 0.02 (-0.32 to 0.4) |
| Panama | 13.1 (8.7 to 17.2) | 500.5 (331.3 to 658.7) | 548.9 (362.9 to 722) | 9.1 (5.1 to 12.9) | 227.6 (126 to 324.7) | 218.4 (122.1 to 310.1) | -2.69 (-3.02 to -2.36) | -0.31 (-0.5 to -0.08) |
| Papua New Guinea | 124.3 (85.7 to 173.3) | 2929 (2072.1 to 3938) | 3041.6 (2096.8 to 4240.9) | 165.4 (109.6 to 233.9) | 1732 (1164.9 to 2428.5) | 1675.9 (1111.1 to 2370.5) | -1.83 (-1.99 to -1.68) | 0.33 (-0.12 to 0.99) |
| Paraguay | 46.4 (33.8 to 60.5) | 865.6 (637.8 to 1116) | 1146.6 (835.4 to 1495.3) | 11.1 (6.2 to 16.7) | 173 (96.1 to 259.3) | 160 (88.8 to 240.3) | -5.55 (-5.86 to -5.24) | -0.76 (-0.85 to -0.65) |
| Peru | 382.8 (233.5 to 562) | 1332.4 (817.3 to 1937.4) | 1761.4 (1074.4 to 2586.4) | 68.3 (35.1 to 104.4) | 210.1 (107.6 to 322.4) | 200.8 (103.1 to 307) | -6.7 (-7.35 to -6.05) | -0.82 (-0.89 to -0.71) |
| Philippines | 1468.6 (1010.1 to 1937.2) | 1825.3 (1274.6 to 2391.8) | 2320.4 (1596 to 3061) | 425.4 (299.3 to 549.4) | 402.2 (279 to 526.6) | 379.3 (266.9 to 489.9) | -5.04 (-5.18 to -4.9) | -0.71 (-0.79 to -0.58) |
| Poland | 28.2 (13 to 44.8) | 79.6 (37.8 to 126.5) | 73.8 (34.1 to 117.4) | 11.6 (4.3 to 20.8) | 34.9 (12.8 to 62.6) | 30.3 (11.2 to 54.1) | -2.8 (-2.91 to -2.68) | -0.59 (-0.75 to -0.42) |
| Portugal | 3.3 (1.5 to 5.2) | 41.4 (19 to 65.8) | 32.3 (14.9 to 51.7) | 0.9 (0.3 to 1.8) | 7.3 (2.3 to 15.8) | 8.1 (2.6 to 17.3) | -5.83 (-6.13 to -5.53) | -0.74 (-0.86 to -0.58) |
| Puerto Rico | 3.4 (2.2 to 4.8) | 97.5 (61.6 to 135.3) | 94.9 (59.7 to 131.7) | 6.4 (3.9 to 8.9) | 151.4 (91 to 211.9) | 181.8 (109.5 to 253.8) | 1.52 (1.39 to 1.65) | 0.87 (0.64 to 1.11) |
| Qatar | 0.5 (0.3 to 0.7) | 111.9 (61.2 to 168.4) | 102.2 (56.3 to 155.4) | 1.3 (0.6 to 2.1) | 69.1 (32.3 to 112.6) | 44.8 (20.4 to 74.7) | -1.63 (-1.76 to -1.5) | 1.82 (0.91 to 2.8) |
| Republic of Korea | 10.6 (3.9 to 19.2) | 32 (11.5 to 58.5) | 23.9 (8.8 to 43.4) | 5.8 (1.8 to 12) | 8 (2.5 to 16.2) | 10.9 (3.5 to 22.5) | -4.62 (-4.77 to -4.47) | -0.45 (-0.68 to -0.12) |
| Republic of Moldova | 11.3 (7.6 to 14.7) | 276.2 (185.4 to 355.7) | 254.8 (171.4 to 330.2) | 3.8 (2 to 5.8) | 112 (60.3 to 168) | 102.7 (53.4 to 155.9) | -2.93 (-3.38 to -2.48) | -0.67 (-0.76 to -0.58) |
| Romania | 52.6 (32.8 to 72.5) | 269.9 (171.3 to 362.9) | 224.9 (140.3 to 309.8) | 13 (5.5 to 22.4) | 77.9 (31.8 to 136.1) | 67.6 (28.6 to 116.6) | -4.18 (-4.44 to -3.92) | -0.75 (-0.85 to -0.66) |
| Russian Federation | 196.9 (104.9 to 295.8) | 149.3 (80.6 to 221.4) | 130.4 (69.5 to 195.9) | 103.7 (44.6 to 174.9) | 75.6 (32.8 to 128.3) | 70.7 (30.4 to 119.2) | -2.29 (-2.5 to -2.08) | -0.47 (-0.64 to -0.31) |
| Rwanda | 1051.7 (691.9 to 1467.1) | 9607.4 (6233 to 13479.9) | 14662.9 (9645.6 to 20454.2) | 193.5 (113 to 305.8) | 1576.1 (916.2 to 2411.9) | 1525.4 (890.5 to 2410.3) | -5.6 (-6.53 to -4.65) | -0.82 (-0.89 to -0.7) |
| Saint Kitts and Nevis | 0.3 (0.2 to 0.4) | 715.3 (522.3 to 896.7) | 715.7 (521.5 to 902.3) | 0.1 (0.1 to 0.2) | 271.9 (179.8 to 362.9) | 245 (161.7 to 330) | -3.15 (-3.44 to -2.85) | -0.51 (-0.61 to -0.38) |
| Saint Lucia | 0.6 (0.4 to 0.8) | 408.7 (307.3 to 504.9) | 439.2 (323.8 to 553.5) | 0.4 (0.2 to 0.5) | 218.8 (142.7 to 301.8) | 204.2 (132.5 to 280.5) | -2.11 (-2.26 to -1.96) | -0.41 (-0.55 to -0.26) |
| Saint Vincent and the Grenadines | 0.7 (0.5 to 0.9) | 591.8 (440.3 to 752.2) | 617.9 (456.3 to 794) | 0.3 (0.2 to 0.3) | 239.2 (163.9 to 316.5) | 222.5 (152.6 to 293.7) | -3 (-3.15 to -2.86) | -0.63 (-0.72 to -0.52) |
| Samoa | 0.5 (0.3 to 0.8) | 409.3 (231.2 to 626) | 319.1 (187.5 to 481.7) | 0.4 (0.2 to 0.7) | 251.7 (131.3 to 379.3) | 212.1 (112.5 to 317.8) | -1.5 (-1.6 to -1.4) | -0.14 (-0.41 to 0.2) |
| San Marino | 0 (0 to 0) | 3.2 (0.9 to 7.3) | 3 (0.9 to 6.7) | 0 (0 to 0) | 1.6 (0.5 to 3.7) | 1.5 (0.5 to 3.6) | -2.4 (-2.55 to -2.25) | -0.27 (-0.56 to 0.19) |
| Sao Tome and Principe | 7.6 (5.1 to 10.9) | 4084.8 (2758.4 to 5696.2) | 6256.6 (4158.2 to 8946) | 0.7 (0.5 to 1) | 412.5 (255.8 to 593.5) | 356.2 (224.5 to 509.4) | -7.68 (-9.25 to -6.08) | -0.9 (-0.94 to -0.85) |
| Saudi Arabia | 152.5 (84.8 to 252.5) | 787.1 (469.2 to 1215.5) | 950.4 (528.7 to 1573.7) | 28.5 (13.4 to 46.6) | 110.2 (53 to 179.7) | 79.8 (37.6 to 130.3) | -6.55 (-6.67 to -6.42) | -0.81 (-0.9 to -0.66) |
| Senegal | 894.1 (616.9 to 1200.6) | 7821.4 (5389.6 to 10203.1) | 11733 (8095.7 to 15754.6) | 341.2 (191.8 to 537.6) | 2153.2 (1264.2 to 3283.7) | 2254.6 (1267.1 to 3552) | -4.35 (-5.54 to -3.14) | -0.62 (-0.78 to -0.38) |
| Serbia | 8.4 (3.9 to 13.8) | 94.7 (43.6 to 156.4) | 89.2 (41.2 to 146.7) | 4.1 (1.5 to 7.4) | 52.5 (18.8 to 94.5) | 47.4 (17.4 to 84.9) | -1.98 (-2.1 to -1.87) | -0.51 (-0.67 to -0.36) |
| Seychelles | 0.2 (0.1 to 0.2) | 240.3 (139.6 to 353.7) | 216.9 (128.1 to 316) | 0.2 (0.1 to 0.2) | 165.3 (98 to 231) | 155.5 (92.6 to 218.3) | -1.16 (-1.46 to -0.86) | 0 (-0.2 to 0.24) |
| Sierra Leone | 490.9 (319.9 to 682.1) | 8927.7 (5972.7 to 12207.4) | 13440.6 (8758.2 to 18676.2) | 187 (65 to 362.7) | 2407.3 (1042.3 to 4215.4) | 2256.9 (784.2 to 4378.5) | -4.41 (-4.76 to -4.07) | -0.62 (-0.86 to -0.24) |
| Singapore | 0.5 (0.2 to 1) | 21.2 (8.5 to 38.3) | 17.4 (6.9 to 31.8) | 0.4 (0.1 to 0.8) | 5.7 (2 to 11.2) | 7.3 (2.5 to 14.7) | -4.31 (-4.65 to -3.97) | -0.22 (-0.54 to 0.24) |
| Slovakia | 4.7 (2 to 8) | 90.6 (38.1 to 155.9) | 88.3 (37.1 to 151.9) | 1.8 (0.6 to 3.7) | 38 (12.9 to 77.3) | 33.6 (11.5 to 67.7) | -2.95 (-3.17 to -2.73) | -0.61 (-0.76 to -0.42) |
| Slovenia | 0.9 (0.3 to 1.7) | 49.6 (19 to 94.8) | 46.8 (17.7 to 88.4) | 0.4 (0.1 to 0.9) | 24.4 (7.8 to 50.5) | 21.1 (6.8 to 43.5) | -2.49 (-2.77 to -2.2) | -0.53 (-0.72 to -0.27) |
| Solomon Islands | 14.1 (8.7 to 21) | 4984.3 (3074.2 to 7408.3) | 4150.5 (2542.8 to 6178.8) | 9.1 (5.9 to 13) | 1920.2 (1187.3 to 2877.7) | 1387.9 (894.4 to 1982.9) | -3.27 (-3.35 to -3.18) | -0.36 (-0.57 to 0.01) |
| Somalia | 697.9 (410.3 to 1031.6) | 8081.1 (4427.4 to 12669.3) | 9763.8 (5740.4 to 14432.7) | 1028.5 (626.5 to 1572.7) | 4286.4 (2579.7 to 6586.1) | 5055.9 (3079.6 to 7730.8) | -2.1 (-2.57 to -1.63) | 0.47 (-0.12 to 1.45) |
| South Africa | 1232.4 (863.3 to 1665.6) | 3003.7 (2102.3 to 4053.4) | 3346.2 (2344.1 to 4522.5) | 479.5 (306 to 687.7) | 955.4 (604.1 to 1368.3) | 862.5 (550.5 to 1237.2) | -3.84 (-4.04 to -3.64) | -0.61 (-0.7 to -0.49) |
| South Sudan | 387.2 (234.3 to 679.6) | 5336.3 (3421.3 to 8345.3) | 6608 (3999.4 to 11599) | 392.2 (243.3 to 594.9) | 3763.4 (2382.6 to 5485.8) | 4224.5 (2621.1 to 6409) | -1.15 (-1.93 to -0.37) | 0.01 (-0.41 to 0.77) |
| Spain | 1.8 (0.6 to 4) | 5.3 (1.7 to 11.6) | 4.7 (1.5 to 10.3) | 2 (0.6 to 4.3) | 3.6 (1.1 to 8.1) | 4.3 (1.4 to 9.3) | -1.28 (-1.4 to -1.16) | 0.09 (-0.34 to 0.83) |
| Sri Lanka | 94 (63.4 to 126.1) | 704.6 (454.9 to 970.5) | 545.6 (368.2 to 732.1) | 33.2 (20.2 to 48.6) | 154.2 (93.9 to 225.8) | 151.9 (92.3 to 222.3) | -5.14 (-5.38 to -4.9) | -0.65 (-0.74 to -0.53) |
| Sudan | 1637.4 (458.6 to 3535.9) | 4148.6 (1301.4 to 8830.6) | 8106.6 (2270.2 to 17505.5) | 502.4 (239.4 to 899.5) | 1015.6 (520.9 to 1704.4) | 1231.1 (586.6 to 2204.2) | -4.55 (-5.04 to -4.06) | -0.69 (-0.84 to -0.06) |
| Suriname | 5.2 (3.7 to 6.6) | 1265.4 (907.5 to 1617.9) | 1333.4 (947.2 to 1714.9) | 1.9 (1.3 to 2.7) | 373 (245 to 512.7) | 337.3 (221.7 to 461.6) | -4.19 (-4.38 to -4) | -0.62 (-0.73 to -0.48) |
| Sweden | 0.4 (0.1 to 0.9) | 4.7 (1.5 to 10.6) | 4.8 (1.5 to 10.8) | 0.4 (0.1 to 1) | 2.8 (0.9 to 6.2) | 4.3 (1.3 to 9.4) | -1.74 (-2.08 to -1.4) | 0.05 (-0.35 to 0.76) |
| Switzerland | 0.2 (0.1 to 0.4) | 3 (0.9 to 6.7) | 2.8 (0.8 to 6.4) | 0.2 (0.1 to 0.4) | 1.7 (0.5 to 4) | 2.1 (0.7 to 4.6) | -1.94 (-2.35 to -1.52) | -0.07 (-0.4 to 0.47) |
| Syrian Arab Republic | 86 (45.4 to 136.3) | 408.4 (226.7 to 628.1) | 666.6 (351.9 to 1056.7) | 15.7 (7.9 to 24.6) | 119.7 (60.6 to 185.1) | 108.4 (54.7 to 170) | -4.14 (-4.48 to -3.8) | -0.82 (-0.89 to -0.68) |
| Taiwan (Province of China) | 12.2 (6.6 to 18.2) | 70.3 (38.2 to 104.4) | 59.8 (32.3 to 89.2) | 7.6 (2.6 to 14) | 31 (10.4 to 57.2) | 32.3 (10.8 to 59.2) | -2.73 (-2.92 to -2.53) | -0.37 (-0.65 to -0.12) |
| Tajikistan | 251.9 (166 to 338.5) | 2701.9 (1796 to 3620.6) | 4686.6 (3087.2 to 6297.2) | 56 (29.9 to 85.6) | 497.2 (269.6 to 750.1) | 589.6 (315.1 to 901.3) | -5.93 (-6.63 to -5.24) | -0.78 (-0.86 to -0.66) |
| Thailand | 307.2 (198.3 to 458.8) | 672.9 (435.4 to 1000.1) | 540.1 (348.6 to 806.7) | 165.9 (107.5 to 239.4) | 211.3 (143.7 to 293.4) | 236.6 (153.4 to 341.5) | -3.85 (-4.11 to -3.58) | -0.46 (-0.65 to -0.22) |
| Timor-Leste | 35.5 (19.9 to 56.2) | 3291.7 (1997.8 to 4956.1) | 4535.3 (2542.9 to 7172.2) | 9.7 (4.7 to 15.7) | 765 (391.1 to 1215) | 728.7 (351.8 to 1173.6) | -4.98 (-5.32 to -4.63) | -0.73 (-0.85 to -0.49) |
| Togo | 551 (279.2 to 784.5) | 9097.7 (5241.9 to 12586) | 15042.1 (7622.1 to 21417) | 384.2 (175.1 to 587.7) | 4038.9 (2092.6 to 5933.8) | 4849.6 (2210.3 to 7418.7) | -2.7 (-2.98 to -2.41) | -0.3 (-0.55 to 0.08) |
| Tokelau | 0 (0 to 0) | 768.1 (447.9 to 1241.6) | 777.6 (470.7 to 1215) | 0 (0 to 0) | 285.3 (169.4 to 427.9) | 279.7 (166.3 to 413.5) | -3.33 (-3.46 to -3.19) | -0.7 (-0.79 to -0.57) |
| Tonga | 0.3 (0.2 to 0.4) | 315.9 (213.3 to 441.4) | 279.6 (187.6 to 375.1) | 0.2 (0.1 to 0.3) | 209 (125.7 to 301.3) | 197 (117.1 to 285.3) | -1.36 (-1.6 to -1.11) | -0.26 (-0.45 to -0.05) |
| Trinidad and Tobago | 2.8 (2 to 3.4) | 245.3 (174 to 303.7) | 229.7 (163.3 to 285.7) | 1.7 (1.1 to 2.4) | 134.5 (83.6 to 189) | 125 (78.6 to 173.9) | -2.02 (-2.36 to -1.69) | -0.37 (-0.52 to -0.23) |
| Tunisia | 19.5 (10.7 to 36.1) | 205.4 (117.8 to 351.4) | 231.4 (127.1 to 428.3) | 13.8 (7.7 to 20.6) | 128 (72.4 to 192) | 119.2 (66.7 to 178.2) | -1.57 (-1.66 to -1.49) | -0.29 (-0.63 to 0.05) |
| Turkey | 245.6 (146.8 to 398.8) | 348.2 (213.7 to 548.6) | 410.9 (245.6 to 667.2) | 107.4 (61.2 to 158.3) | 150.6 (86.2 to 220.5) | 132 (75.2 to 194.5) | -2.71 (-3.05 to -2.37) | -0.56 (-0.76 to -0.31) |
| Turkmenistan | 90.5 (61.2 to 116.3) | 1532.9 (1035.4 to 1962.2) | 2443.4 (1652.7 to 3139.2) | 7.5 (4.1 to 10.9) | 142.2 (77.5 to 205.9) | 147.4 (80.3 to 213.7) | -7.58 (-8.41 to -6.75) | -0.92 (-0.94 to -0.88) |
| Tuvalu | 0.1 (0.1 to 0.2) | 1235.6 (720.7 to 2008.1) | 1086.5 (662.2 to 1716.4) | 0 (0 to 0.1) | 394.9 (229.4 to 587) | 346.1 (203.5 to 508.4) | -3.84 (-4.01 to -3.67) | -0.6 (-0.72 to -0.42) |
| Uganda | 1457.8 (808.1 to 2381.5) | 4985 (2944 to 7463.3) | 8419 (4666.7 to 13753.4) | 611.7 (375.2 to 955.3) | 1484.6 (887.9 to 2243.6) | 1487.8 (912.6 to 2323.4) | -4.13 (-4.51 to -3.75) | -0.58 (-0.77 to -0.19) |
| Ukraine | 44.2 (23.1 to 67.2) | 94.5 (50 to 142.8) | 84 (43.9 to 127.7) | 32.5 (15.3 to 52) | 79.7 (37.9 to 129.1) | 73.7 (34.8 to 118.1) | -0.5 (-0.63 to -0.37) | -0.27 (-0.42 to -0.1) |
| United Arab Emirates | 2.1 (1.2 to 3.2) | 162.8 (85.2 to 256.9) | 113.1 (62.8 to 168.7) | 5.9 (2.7 to 9.7) | 104.1 (45.8 to 177.9) | 63.7 (28.7 to 105.2) | -1.5 (-1.72 to -1.28) | 1.78 (0.88 to 2.69) |
| United Kingdom | 2.4 (0.7 to 5.3) | 4.4 (1.3 to 9.9) | 4.1 (1.2 to 9.2) | 1.6 (0.5 to 3.6) | 2.1 (0.6 to 4.7) | 2.4 (0.7 to 5.3) | -2.49 (-2.68 to -2.3) | -0.33 (-0.59 to 0.12) |
| United Republic of Tanzania | 2056.6 (1397.5 to 2828.7) | 5585.8 (3846.4 to 7435.1) | 7940.1 (5395.6 to 10921.2) | 704.3 (453 to 1033.2) | 1414.4 (874.5 to 2211.2) | 1241.3 (798.4 to 1821.1) | -4.59 (-5.21 to -3.96) | -0.66 (-0.78 to -0.46) |
| United States of America | 26.5 (8.8 to 56.2) | 10.9 (3.6 to 22.9) | 10.5 (3.5 to 22.1) | 14.5 (4.8 to 30.6) | 3.9 (1.3 to 8.2) | 4.4 (1.5 to 9.3) | -3.59 (-3.99 to -3.18) | -0.45 (-0.66 to -0.1) |
| United States Virgin Islands | 0.1 (0.1 to 0.2) | 145.7 (93.9 to 199.9) | 139.3 (89.2 to 191.2) | 0.2 (0.1 to 0.2) | 140.6 (81.6 to 204.4) | 158.5 (92.2 to 228.1) | -0.07 (-0.16 to 0.01) | 0.12 (-0.1 to 0.31) |
| Uruguay | 4 (2.2 to 5.6) | 133.4 (73.8 to 190.1) | 126.4 (70.5 to 179.4) | 1.8 (0.8 to 3.1) | 47.6 (20.4 to 80.4) | 53.4 (22.9 to 90.4) | -3.46 (-3.71 to -3.2) | -0.54 (-0.7 to -0.37) |
| Uzbekistan | 239.4 (133.1 to 321.8) | 732.6 (405.5 to 984.3) | 1142.7 (635.3 to 1536) | 30.2 (13.9 to 47.6) | 87.4 (40.2 to 137.4) | 89.8 (41.2 to 141.3) | -7.26 (-7.68 to -6.82) | -0.87 (-0.92 to -0.82) |
| Vanuatu | 2.8 (1.5 to 4.3) | 2048.2 (1206.4 to 3121.8) | 1844.8 (986.6 to 2807.8) | 2.6 (1.6 to 3.7) | 1111.6 (683.3 to 1662.1) | 886.1 (559.9 to 1269.5) | -2.1 (-2.31 to -1.89) | -0.07 (-0.37 to 0.42) |
| Venezuela (Bolivarian Republic of) | 218.5 (149.6 to 272.2) | 920.4 (632.5 to 1144.1) | 1160.4 (794.4 to 1445.4) | 50.8 (26.8 to 74.5) | 196.4 (104.1 to 287) | 180.8 (95.6 to 265.4) | -4.88 (-5.8 to -3.96) | -0.77 (-0.83 to -0.7) |
| Viet Nam | 445.7 (242.8 to 737.7) | 590.1 (343.2 to 932.7) | 656 (357.3 to 1085.9) | 93.7 (58.4 to 131) | 111.3 (69.6 to 155.6) | 97.2 (60.6 to 135.9) | -5.62 (-5.82 to -5.43) | -0.79 (-0.88 to -0.61) |
| Yemen | 1446.7 (542 to 2700) | 5020.2 (2040.8 to 9109.3) | 10537.3 (3947.7 to 19666.4) | 506.8 (219.8 to 958) | 1237.8 (588.3 to 2249.2) | 1608.6 (697.7 to 3041) | -4.62 (-5.11 to -4.13) | -0.65 (-0.76 to -0.43) |
| Zambia | 785.5 (517.1 to 1119.2) | 6654.9 (4560.1 to 9428.5) | 9888.9 (6509.7 to 14090.1) | 415.6 (275.2 to 604.5) | 2108.9 (1381.6 to 3075.2) | 2278.5 (1508.9 to 3314.4) | -3.85 (-4.15 to -3.55) | -0.47 (-0.67 to -0.11) |
| Zimbabwe | 189.5 (128.5 to 267.1) | 1786.9 (1148.7 to 2408.8) | 1833.3 (1242.9 to 2583.4) | 221.2 (132.7 to 319.8) | 1672.5 (986.4 to 2380.1) | 1473.6 (884 to 2130.6) | -0.4 (-2.23 to 1.46) | 0.17 (-0.25 to 0.76) |

DALYs: disability-adjusted life years

**Supplementary Figure 1. Average annual percent change (AAPC) of disability-adjusted life years (DALYs) of diarrheal diseases resulting from unsafe water by country for male and female sexes combined and all ages from 1990 to 2019.**


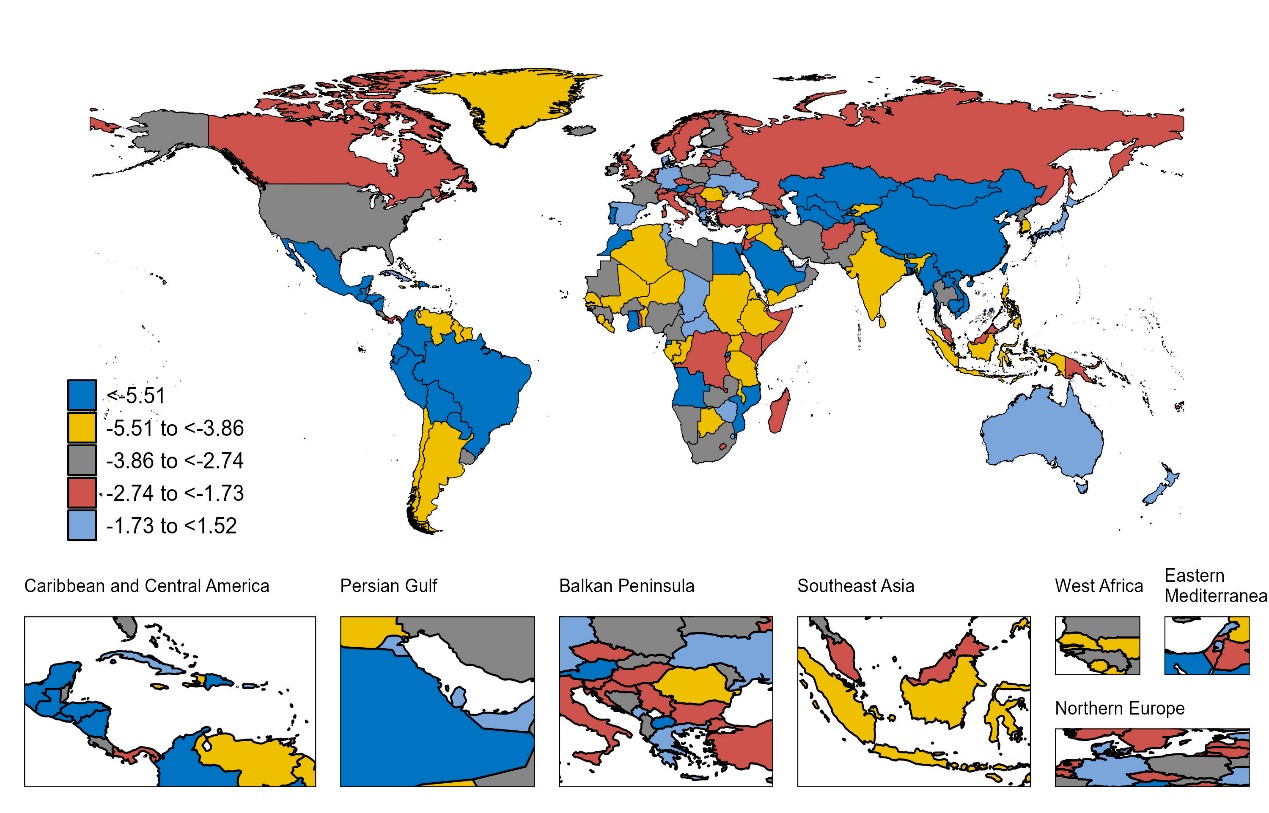


**Supplementary Figure 2. Age-standardized deaths rate of diarrheal diseases attributable to unsafe water across 21 GBD regions by socio-demographic index for both sexes, 1990–2019.**


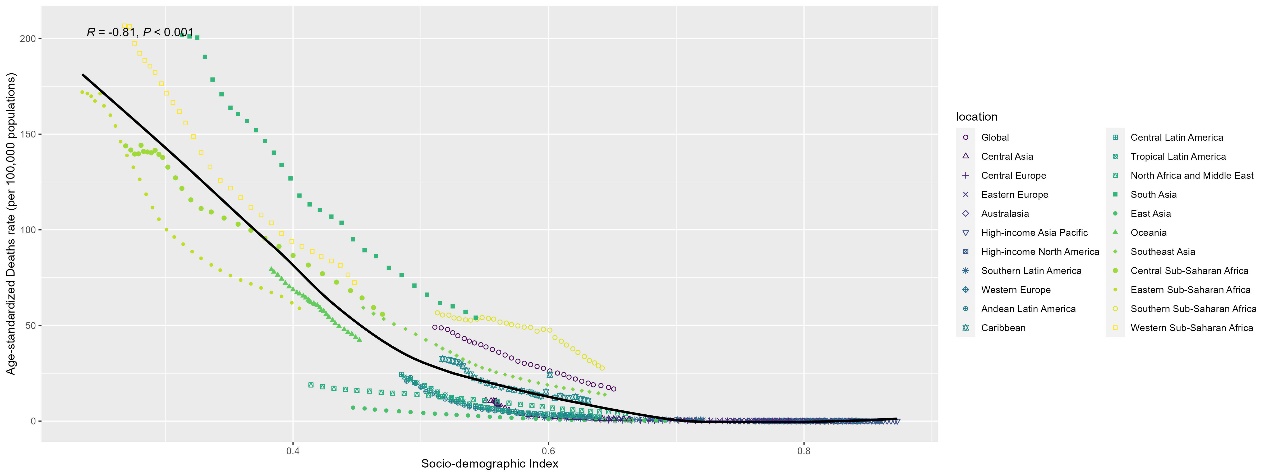


**Supplementary Figure 3. Age-standardized DALYs rate of diarrheal diseases attributable to unsafe water across 21 GBD regions by socio-demographic index for both sexes, 1990–2019.**


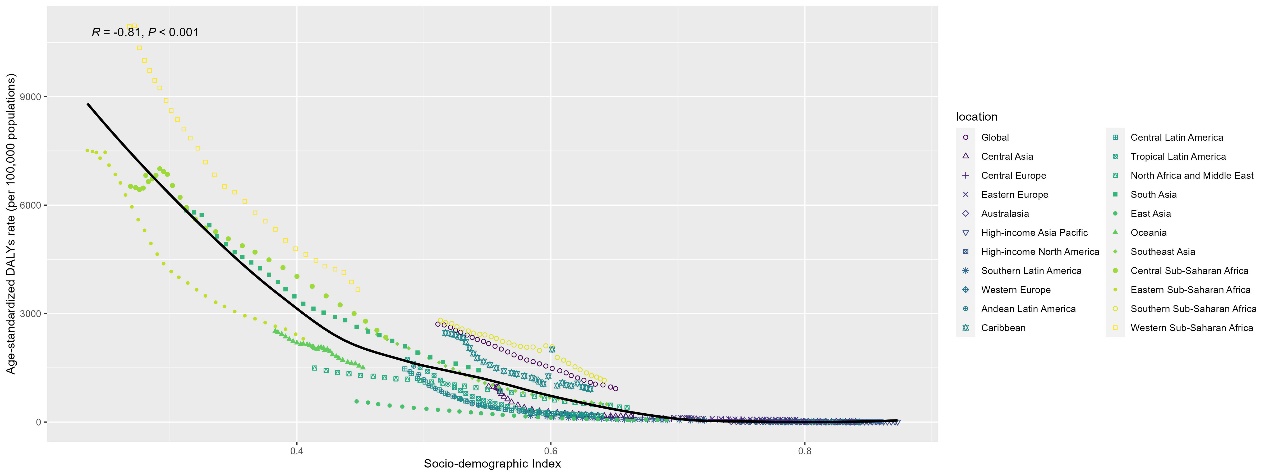


**Supplementary Figure 4. Age-standardized deaths rate of diarrheal diseases attributable to unsafe water across 204 countries by socio-demographic index for both sexes, 1990–2019.**


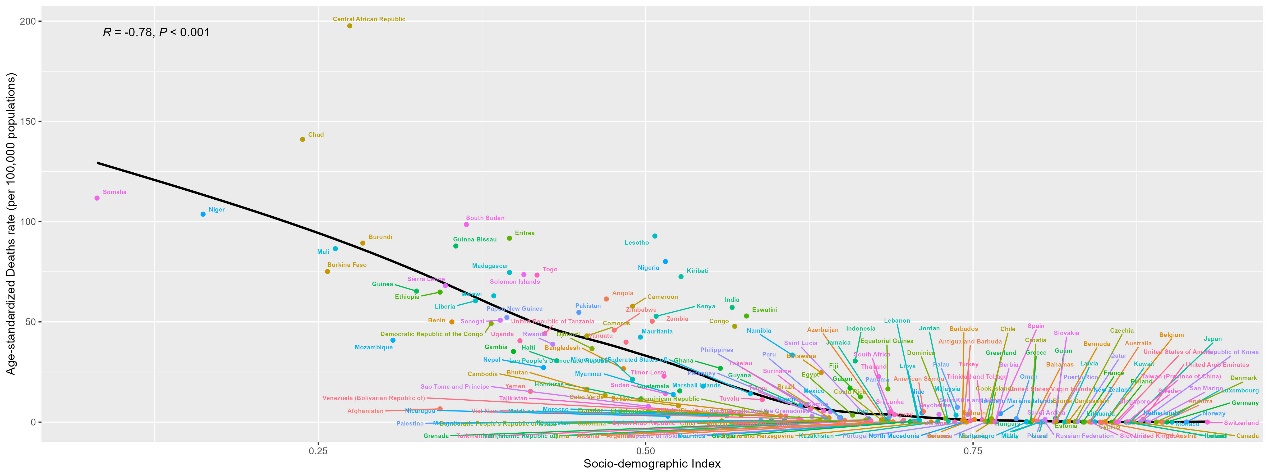


**Supplementary Figure 5. Age-standardized DALYs rate of diarrheal diseases attributable to unsafe water across 204 countries by socio-demographic index for both sexes, 1990–2019.**


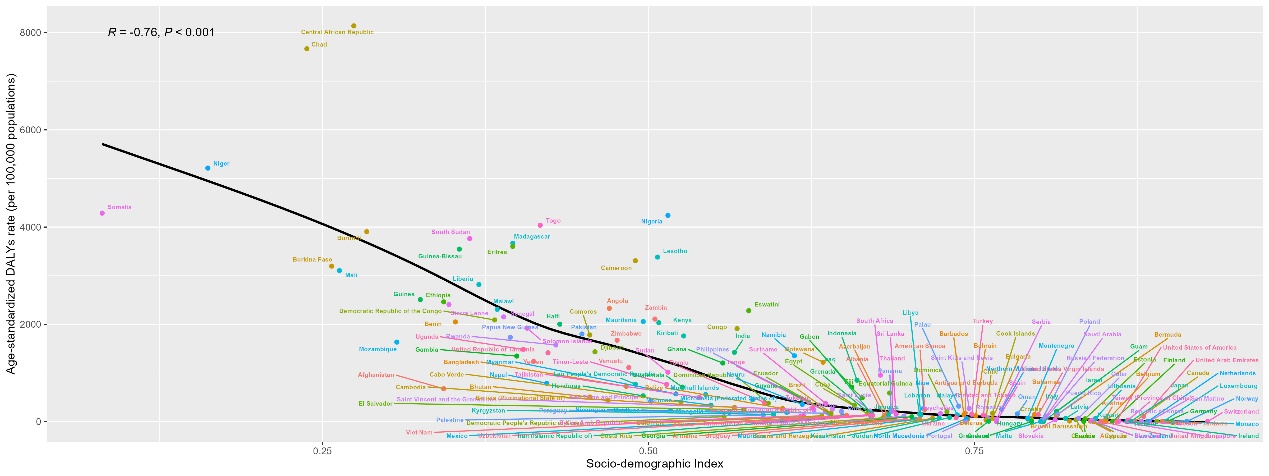

Supplement: Supplementary file 1 [file Data_Sheet_1.docx]
